# Secondary metabolites from the deep-sea derived fungus *Aspergillus terreus* MCCC M28183

**DOI:** 10.3389/fmicb.2024.1361550

**Published:** 2024-02-14

**Authors:** Xiaomei Huang, Yichao Wang, Guangyu Li, Zongze Shao, Jinmei Xia, Jiang-Jiang Qin, Weiyi Wang

**Affiliations:** ^1^Department of Marine Biology, Xiamen Key Laboratory of Intelligent Fishery, Xiamen Ocean Vocational College, Xiamen, China; ^2^Key Laboratory of Marine Biogenetic Resources, Third Institute of Oceanography, Ministry of Natural Resources, Xiamen, China; ^3^Zhejiang Cancer Hospital, Hangzhou Institute of Medicine (HIM), Chinese Academy of Sciences, Hangzhou, China; ^4^College of Pharmaceutical Sciences, Zhejiang University of Technology, Hangzhou, China

**Keywords:** *Aspergillus*, secondary metabolites, cytotoxic, antibacterial, deep-sea

## Abstract

*Aspergillus* fungi are renowned for producing a diverse range of natural products with promising biological activities. These include lovastatin, itaconic acid, terrin, and geodin, known for their cholesterol-regulating, anti-inflammatory, antitumor, and antibiotic properties. In our current study, we isolated three dimeric nitrophenyl trans-epoxyamides (**1**–**3**), along with fifteen known compounds (**4**–**18**), from the culture of *Aspergillus terreus* MCCC M28183, a deep-sea-derived fungus. The structures of compounds **1**–**3** were elucidated using a combination of NMR, MS, NMR calculation, and ECD calculation. Compound **1** exhibited moderate inhibitory activity against human gastric cancer cells MKN28, while compound **7** showed similar activity against MGC803 cells, with both showing IC_50_ values below 10 μM. Furthermore, compound **16** exhibited moderate potency against *Vibrio parahaemolyticus* ATCC 17802, with a minimum inhibitory concentration (MIC) value of 7.8 μg/mL. This promising research suggests potential avenues for developing new pharmaceuticals, particularly in targeting specific cancer cell lines and combating bacterial infections, leveraging the unique properties of these *Aspergillus*-derived compounds.

## 1 Introduction

*Aspergillus terreus*, a prolific filamentous fungus, synthesizes an array of secondary metabolites with distinct biological functions. Lovastatin, a prominent metabolite, exhibits a unique hexahydronaphthalene ring and a 2-methylbutyric acid moiety, efficaciously inhibiting HMG-CoA reductase for cholesterol regulation (Huang et al., 2021). Itaconic acid, another metabolite, serves as an industrial precursor and exhibits potential anti-inflammatory properties (Diankristanti and Ng, 2023). Terrein, with its 4H-furan-3-one framework, demonstrates multifaceted biological activities, including antitumor and anti-inflammatory effects, and inhibits melanin synthesis, suggesting therapeutic applications in hyper-pigmentation (Zhao et al., 2016). The dihydroxyphenylalanine (DOPA) pigments, geodin and *epi*-geodin, are noted for their antibiotic activity and metal ion chelation capabilities (Boruta et al., 2021). This spectrum of metabolites, each with unique structural and pharmacological properties, underscores the importance of *Aspergillus terreus* in pharmaceutical and industrial applications. Ongoing research in this domain is crucial for advancing natural product chemistry and drug discovery, underscoring the significant role of fungi in producing bioactive compounds.

**Figure 1:**
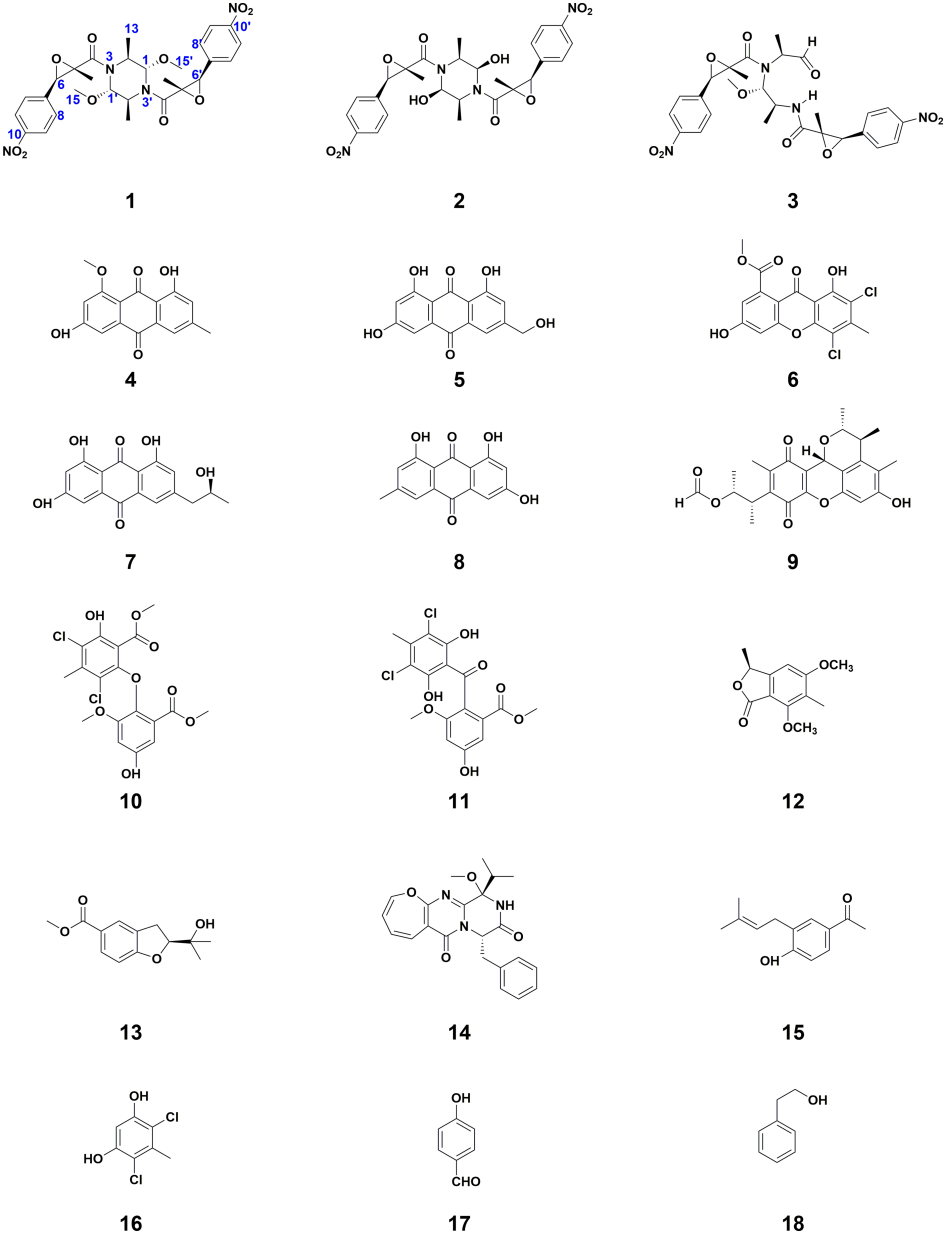
Structures of compounds **1–18**.

In exploring marine-derived bioactive compounds, a cytotoxic dihydrobenzofuran was extracted from the deep-sea fungus *Aspergillus terreus* CC-S06-18 (Wang et al., 2020). Following this, extensive large-scale fermentation enabled the identification and characterization of eighteen secondary metabolites. This included three novel dimeric nitrophenyl trans-epoxyamides (**1**–**3**) and an additional fifteen compounds (**4**–**18**) (Figure 1). Subsequently, each of these compounds underwent evaluation to determine their cytotoxic and antibacterial activities.

## 2 Materials and methods

### 2.1 Fungal material

The *Aspergillus terreus* strain, initially isolated from a seawater sample taken at a depth of 5,250 meters in the Pacific Ocean and originally labeled as CC-S06-18, has had its ITS sequence submitted to GenBank, under the sequence number MN463005. Additionally, this strain is currently preserved in the Marine Culture Collection of China (MCCC), with the preservation number M28183.

### 2.2 Fermentation and extraction

The fermentation process was conducted statically using two hundred 1-liter Erlenmeyer flasks, each filled with 30 grams of millet, 70 grams of rice, and 100 milliliters of seawater. Following the inoculation, the flasks were left to incubate at room temperature for 28 days. Subsequently, the product of fermentation was subjected to three rounds of extraction using ethyl acetate. The final step involved filtering and concentrating the extract, resulting in a total yield of 85 grams.

### 2.3 Isolation and purification

The purification process of the crude extract was systematically executed using silica gel column chromatography with a petroleum ether and ethyl acetate mixture as the eluent, progressing through gradients of 9.5:0.5 to 8.0:2.0 (v/v). This approach resulted in six fractions (Frs1-6). Subsequent purification of Fr3 via Sephadex LH-20 chromatography in MeOH yielded five subfractions (Fr3.1-Fr3.5). Specifically, Fr3.1 was further refined using the same chromatographic technique to isolate **8** (136.8 mg). Meanwhile, Fr3.4 and Fr3.5 underwent RP-HPLC with a 20–80% MeCN in H_2_O gradient over 20.0 min at a 15 mL/min flow rate. This procedure led to the isolation of **15** (1.0 mg, *t*_R_ = 13.3 min) and **14** (2.2 mg, *t*_R_ = 18.5 min) from Fr3.4, and **17** (2.3 mg, *t*_R_ = 16.0 min) from Fr3.5. Fr4 was similarly fractionated using Sephadex LH-20 in MeOH, resulting in four subfractions (Fr4.1-Fr4.4). The RP-HPLC purification of Fr4.2, with a nuanced protocol involving 20–80% MeCN, followed by 80–100% MeCN, and finally 100% MeCN, yielded **9** (1.3 mg, *t*_R_ = 24.6 min). The subfractions Fr4.3 and Fr4.4 were also purified by RP-HPLC, leading to the isolation of **18** (1.1 mg, *t*_R_ = 16.4 min) and **6** (1.4 mg, *t*_R_ = 5.5 min), respectively. Further, Fr5 was divided into three subfractions (Fr5.1-Fr5.3) via Sephadex LH-20 in MeOH. The RP-HPLC purification of Fr5.2, with a modified gradient of 30–80% MeCN in H_2_O, followed by 80–100% MeCN, and finally 100% MeCN, resulted in the isolation of **13** (4.3 mg, *t*_R_ = 18.4 min), **1** (2.6 mg, *t*_R_ = 16.1 min), **4** (1.1 mg, *t*_R_ = 24.5 min), and **2** (1.0 mg, *t*_R_ = 27.1 min). Fr5.3 was processed similarly to yield **10** (1.3 mg, *t*_R_ = 9.0 min) and **16** (1.2 mg, *t*_R_ = 15.7 min). Finally, Fr6 was subdivided into eight subfractions (Fr6.1-Fr6.8) using Sephadex LH-20 in MeOH, with Fr6.1, Fr6.2, and Fr6.3 undergoing RP-HPLC purification (30–60% MeCN in H_2_O, then 60–100% MeCN) to yield **11** (1.6 mg, *t*_R_ = 13.5 min), **7** (2.1 mg, *t*_R_ = 9.9 min), and **5** (0.8 mg, *t*_R_ = 14.0 min) respectively. Fr6.8 was further purified by RP-HPLC with a similar gradient, culminating in 100% MeCN, to isolate **3** (1.2 mg, *t*_R_ = 25.0 min) and **12** (1.6 mg, *t*_R_ = 31.2 min).

#### 2.3.1 Compound 1

A white amorphous powder; [α]25 D − 14.2 (*c* 0.1, MeOH); UV (MeOH) *λ*_max_ (log *ε*): 270 (4.00) nm; ECD (*c* 0.1 mg/mL, MeOH) *λ*_max_ (∆*ε*): 218 (−7.93), 263 (1.71) nm; HRESIMS *m/z* 607.2017 [M + Na]^+^ (calcd for C_28_H_32_N_4_O_10_Na, 607.2016); ^1^H and ^13^C NMR data, Table 1.

**Table 1:** ^1^H (600 MHz) and ^13^C NMR (125 MHz) data of **1**–**3** (Acetone-*d*_6_).

No.	**1**		**2**		**3**	
	*δ*_H_ (mult, *J* in Hz)	*δ*_C_, type	*δ*_H_ (mult, *J* in Hz)	*δ*_C_, type	*δ*_H_ (mult, *J* in Hz)	*δ*_C_, type
1	5.63 (m)	86.4, CH	6.40 (m)	91.3, CH	–	198.3, CH
2	4.66 (m)	47.6, CH	4.08 (qd, 6.9, 3.0)	58.9, CH	3.79 (q, 6.1)	57.4, CH
4	–	172.3, C	–	171.4, C	–	170.7, C
5	-	65.0, C	-	64.1, C	-	64.8, C
6	4.46 (s)	62.7, CH	4.38 (s)	61.7, CH	4.65 (s)	62.9, CH
7	–	142.7, C	–	142.4, C	–	141.9, C
8	7.68 (d, 8.6)	128.8, CH	7.68 (d, 8.6)	128.9, CH	7.74 (d, 8.8)	128.8, CH
9	8.33 (d, 8.6)	124.3, CH	8.32 (d, 8.7)	124.3, CH	8.35 (d, 8.8)	124.5, CH
10	–	148.9, C	–	148.9, C	–	148.8, C
11	8.33 (d, 8.6)	124.3, CH	8.32 (d, 8.7)	124.3, CH	8.35 (d, 8.8)	124.5, CH
12	7.68 (d, 8.6)	128.8, CH	7.68 (d, 8.6)	128.9, CH	7.74 (d, 8.8)	128.8, CH
13	1.31 (d, 7.1)	19.2, CH_3_	1.32 (d, 6.4)	13.7, CH_3_	1.46 (d, 6.4)	15.5, CH_3_
14	1.39 (s)	15.3, CH_3_	1.36 (s)	14.1, CH_3_	1.48 (s)	13.2, CH_3_
15	3.43 (s)	54.9, CH_3_				
1’	5.63 (m)	86.4, CH	6.40 (m)	91.3, CH	5.22 (d, 9.2)	92.8, CH
2’	4.66 (m)	47.6, CH	4.08 (qd, 6.9, 3.0)	58.9, CH	4.25 (m)	46.0, CH
4’	–	172.3, C	–	171.4, C	–	169.5, C
5’	–	65.0, C	–	64.1, C	–	64.8, C
6’	4.46 (s)	62.7, CH	4.38 (s)	61.7, CH	4.42 (s)	63.2, CH
7’	–	142.7, C	–	142.4, C	–	142.3, C
8’	7.68 (d, 8.6)	128.8, CH	7.68 (d, 8.6)	128.9, CH	7.60 (d, 8.8)	128.8, CH
9’	8.33 (d, 8.6)	124.3, CH	8.32 (d, 8.7)	124.3, CH	8.24 (d, 8.8)	124.3, CH
10’	–	148.9, C	–	148.9, C	–	149.1, C
11’	8.33 (d, 8.6)	124.3, CH	8.32 (d, 8.7)	124.3, CH	8.24 (d, 8.8)	124.3, CH
12’	7.68 (d, 8.6)	128.8, CH	7.68 (d, 8.6)	128.9, CH	7.60 (d, 8.7)	128.8, CH
13’	1.31 (d, 7.1)	19.2, CH_3_	1.32 (d, 6.4)	13.7, CH_3_	1.38 (d, 6.5)	18.6, CH_3_
14’	1.39 (s)	15.3, CH_3_	1.36 (s)	14.1, CH_3_	1.24 (s)	12.3, CH_3_
15’	3.43 (s)	54.9, CH_3_			3.62 (s)	58.0, CH_3_

#### 2.3.2 Compound 2

A white amorphous powder; [α]25 D + 11.3 (*c* 0.1, MeOH); UV (MeOH) *λ*_max_ (log *ε*): 270 (3.89) nm; ECD (*c* 0.1 mg/mL, MeOH) *λ*_max_ (∆*ε*): 217 (−7.06), 246 (2.10) nm; HRESIMS *m/z* 555.1711 [M − H]^−^ (calcd for C_26_H_27_N_4_O_10_, 555.1727); ^1^H and ^13^C NMR data, Table 1.

#### 2.3.3 Compound 3

A white amorphous powder; [α]25 D − 62.0 (*c* 0.1, MeOH); UV (MeOH) *λ*_max_ (log *ε*): 269 (4.06) nm; ECD (*c* 0.1 mg/mL, MeOH) *λ*_max_ (∆*ε*): 209 (−10.28), 255 (1.68) nm; HRESIMS *m/z* 593.1859 [M + Na]^+^ (calcd for C_27_H_30_N_4_O_10_Na, 593.1860); ^1^H and ^13^C NMR data, Table 1.

### 2.4 Cytotoxic activity assay

Initially, MGC803 and MKN28 cell lines were dissociated and counted. Following this, 3 × 10^3^ cells from each line were seeded into each well of 96-well plates and incubated overnight to promote cell attachment. During the incubation period, the edge wells of the plate were filled with PBS. Post-attachment, the cells were exposed to increasing concentrations of the test compounds for a growth period of 72 h. Subsequently, 10 μL of CCK8 reagent was added to each well, followed by an incubation at 37°C of 1 h. The absorbance was measured at 450 nm using a microplate reader (Tecan, Morrisville, NC, United States) according to the manufacturer’s instructions. The CCK8 experimental setup was conducted in triplicate. Data were analyzed with GraphPad Prism software to determine IC_50_ of each compound (Wang et al., 2018, 2019; Hong et al., 2022; Lv et al., 2022).

### 2.5 Antimicrobial assay

Antimicrobial activity testing against *Salmonella enteritidis* (CICC 21482), *Vibrio vulnificus* (MCCC 1A08743), *Staphylococcus aureus* (CICC 10384), *Vibrio parahaemolyticus* (ATCC 17802), *Escherichia coli* (CICC 10302), and *Vibrio parahaemolyticus* (vp-HL) (Yang et al., 2022) was conducted using a microdilution method in 96-well plates with resazurin as a growth marker. Resazurin sodium salt was dissolved in sterile water to give a 2.0 mg/mL solution. A tested bacterial strain, during its mid-logarithmic phase and with an initial inoculum of 1 × 10^5^ CFU/mL, was introduced into plates containing serial dilutions of the test compound and a 10% resazurin solution. These plates were covered with foil and incubated at 37°C with shaking for 24 h. Following incubation, a visual inspection was conducted to detect any color change from blue to pink, indicating bacterial growth. The Minimum Inhibitory Concentration (MIC) was determined as the lowest concentration at which no color change occurred. All experiments were conducted in triplicate, and the average MIC values were calculated for the test compound (Wang et al., 2018, 2019; Hong et al., 2022; Lv et al., 2022).

### 2.6 NMR and ECD calculations

Conformers were initially generated using CREST software (Grimme, 2019; Pracht et al., 2020) and subsequently underwent refinement via DFT optimization at the B3LYP/6-31G(d) level in Gaussian 16 (Frisch et al., 2016). This process targeted conformers within a 10 kcal/mol energy window. Frequency analysis was employed to verify the local minimum status of each conformer. Electronic energies were then recalculated at the more advanced M062X/6–311 + G(2d,p) level. The conformer populations were assessed using Boltzmann distribution, focusing on those that accounted for more than 2% of the total for in-depth analysis.

The GIAO method was used for the calculation of NMR shielding constants at mPW1PW91-SCRF/6–31 + G(d,p) level with tetramethylsilane (TMS) as a reference (Willoughby et al., 2014). For each candidate, linear regression parameters a and b (*δ*cal = *aδ*exp + *b*) were calculated, along with the correlation coefficient (R^2^), Mean Absolute Error (MAE, Σn |δcal − δexp|/n), and Corrected Mean Absolute Error (CMAE, Σn |δcorr − δexp|/n), where δcorr = (δcal − b)/a.

ECD calculations were done via TDDFT at the Cam-B3LYP/6-311G(d) level, calculating 36 excited states per conformer (Pescitelli and Bruhn, 2016). Multiwfn 3.6 software was used to generate ECD curves (Lu and Chen, 2012).

## 3 Results and discussion

Compound **1**, a white powder, was analyzed using HRESIMS and demonstrated a sodium adduct ion peak with *m/z* of 607.2017 [M + Na]^+^. This peak suggested the molecular formula of C_28_H_32_N_4_O_10_, indicative of 15 degrees of unsaturation. Examination of the ^13^C NMR and DEPT spectra revealed 14 carbon atoms: comprising two methyl groups (*δ*_C_ 19.2, C-13 and *δ*_C_ 15.3, C-14), a single methoxyl group (*δ*_C_ 54.9, C-15), three methines linked to oxygen or nitrogen (*δ*_C_ 86.4, C-1; *δ*_C_ 47.6, C-2; *δ*_C_ 62.7, C-6), an oxygen-bearing quaternary carbon (*δ*_C_ 65.0, C-5), four aromatic methines (*δ*_C_ 124.3, C-9/11 and *δ*_C_ 128.8, C-8/12), two quaternary aromatic carbons (*δ*_C_ 142.7, C-7 and *δ*_C_ 148.9, C-10), and a carbonyl carbon atom (*δ*_C_ 172.3, C-4) (Table 1). The ^1^H NMR data supplemented these observations, showing signals for three methyl groups (*δ*_H_ 3.43, OCH_3_-1; *δ*_H_ 1.31, H-13; *δ*_H_ 1.39, H-14), three methines related to oxygen or nitrogen (*δ*_H_ 4.46, H-6; *δ*_H_ 5.63, H-1; *δ*_H_ 4.66, H-2), along with four aromatic methines (*δ*_H_ 7.68, d, H-8/12; *δ*_H_ 8.33, d, *J* = 8.6 Hz, H-9/11) (Table 1). Considering the NMR data and its molecular formula, compound **1** was theorized to be a symmetrically structured dimer.

**Figure 2:**
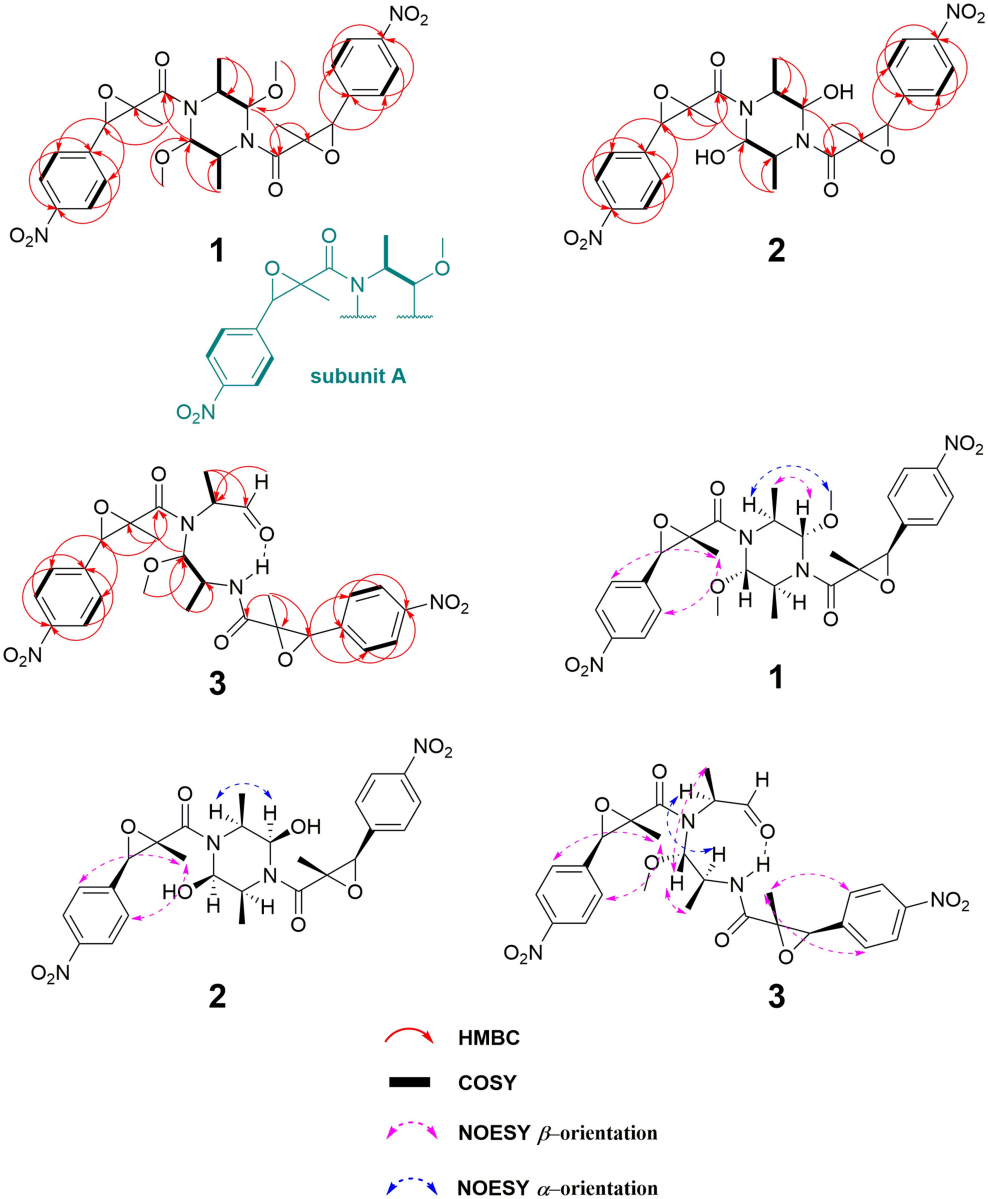
Key HMBC, COSY, and NOESY correlations of **1–3**.

The planar configuration of compound **1** was established through a comprehensive analysis using both 1D and 2D NMR spectroscopy. The existence of a *p*-nitrophenyl group was initially inferred from HMBC spectral correlations, including signals from H-9/H-11 to C-7 (*δ*_C_ 142.7) and C-10 (*δ*_C_ 148.9), and from H-8/H-12 to C-10. These findings were further corroborated by COSY spectral correlations, specifically between H-9 and H-8, and H-11 and H-12 (Figure 2). Further, the structure was identified to contain a 2-methyl-2,3-epoxyamide group, evidenced by HMBC correlations linking H-6 to C-4 and C-5, and H_3_-14 to C-4, C-5, and C-6, with the chemical shifts of C-5 (*δ*_C_ 65.0) and C-6 (*δ*_C_ 62.7) confirming the presence of an epoxy group (Figure 2). The isopropyl segment in the structure was pinpointed through COSY correlations from the methyl group H_3_-13 to H-1 and H-2, and its attachment to N-3 was established by HMBC correlations from H-2 to C-4 (Figure 2). As compound **1** was presumed to be a dimer, the molecular formula of its monomer was deduced to be C_14_H_16_N_2_O_5_. This monomer comprised a 2-methyl-2,3-epoxyamide group, an isopropyl group, and a 1,4-disubstituted phenyl, along with a methoxy group attached to the C-1 position. Considering the remaining atomic composition of one nitrogen and two oxygens in the monomer, it was concluded that a nitro group was attached at the C-10 position. All these data led to the confirmation of subunit A in the structure, as shown in Figure 2. Additionally, the HMBC correlations between H-1 and C-4′, coupled with the chemical shifts of C-1/1′ (*δ*_C_ 86.4) (Figure 2), suggested that two subunit A units are linked via two C-N bonds, forming a dimer with a central symmetrical framework, as represented in Figure 2.

**Figure 3:**
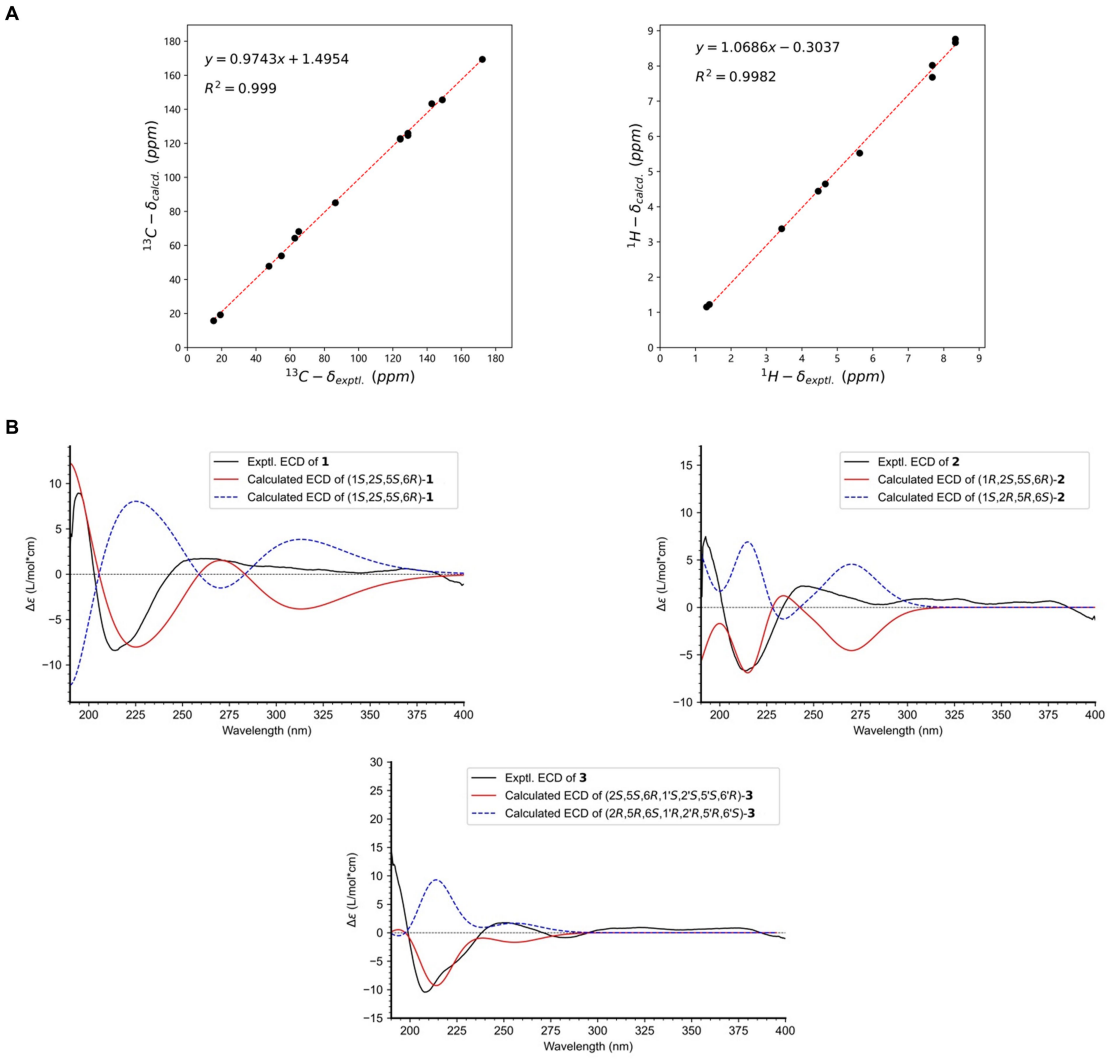
**(A)** Linear regression analysis between the experimental and calculated NMR data of 1; **(B)** Experimental and calculated ECD spectra of **1–3**.

Key NOESY correlations, specifically from H-1,1′ to H_3_-13/13′, as well as between H-2,2′ and H_3_-15,15′, demonstrated that H-1/1′ and H_3_-13/13′ resided on the same piperazine ring, suggesting an *α*-orientation. Further NOESY correlations between H-8,8′ and H_3_-14,14′ suggested that H-6,6′ and H_3_-14,14′ occupy different faces of the oxirane ring (Figure 2). This led to the deduction of two possible relative configurations of (1S*,1’S*,2S*,2’S*,5S*,5’S*,6R*,6’R*)-**1** (**1a**) and (1S*,1’S*,2S*,2’S*,5R*,5’R*,6S*,6’S*)-**1** (**1b**). A conformational search was conducted for these configurations, followed by geometry optimization. The lowest energy conformers identified were subjected to NMR calculations at the mPW1PW91-SCRF/6–31 + G(d,p) level using GIAO method. **1a** had a DP4+ possibility of 100%, with its calculated ^13^C NMR data closely aligned with the experimental results, exhibiting *R*^2^, MAE, and CMAE values of 0.9990, 1.8, and 1.2, respectively (Figure 3A). The absolute configuration was definitely identified as 1*S*,1’*S*,2*S*,2’*S*,5*S*,5’*S*,6*R*,6’*R* through comparative analysis of the experimental ECD spectrum and the CAM-B3LYP/6-311G(d) calculated spectra for both stereoisomers in MeOH. This identification was affirmed as the calculated ECD for (1*S*,1’*S*,2*S*,2’*S*,5*S*,5’*S*,6*R*,6’*R*) closely matched the experimental spectrum (Figure 3B).

Compound **2** has been established to possess the molecular formula C_26_H_28_N_4_O_10_, which was derived from HRESIMS data exhibiting a deprotonated ion peak at a *m/z* of 555.1711 [M − H]^−^. This indicated a reduction of 28 atomic mass units (amu) compared to compound **1**, along with the presence of 15 degrees of unsaturation. A comparative analysis of NMR data between compounds **1** and **2** revealed that the primary variations are concentrated in the piperazine ring. Notably, the chemical shifts at C-1 and C-2 altered from *δ*_C_ 86.4 to *δ*_C_ 91.3, and from *δ*_C_ 47.6 to *δ*_C_ 58.9, respectively. The observation of 13 unique carbon resonances in the ^13^C NMR spectrum, in conjunction with its molecular weight, indicates that compound **2** shared the same symmetrical framework as compound **1**, as detailed in Table 1. In the NOESY spectrum, correlations between H-1,1′ and H-2,2′ in compound **2** suggested that H_3_-13/13′ and H-1/1′ were positioned on opposite sides of the piperazine ring. Furthermore, NOESY correlations between H-8,8′ and H_3_-14,14′ implied that H-6,6′ and H_3_-14,14′ were situated on different faces of the oxirane ring, paralleling the pattern observed in compound **1**, as depicted in Figure 2. Considering the biosynthetic pathway, compound **2** is likely the 1,1′-epimer derivative of compound **1**, characterized by a hydroxyl group attached to both C-1′ and C-1. Finally, the absolute configuration of compound **2** is designated as 1*R*,1’*R*,2*S*,2’*S*,5*S*,5’*S*,6*R*,6’*R* by ECD calculation at CAM-B3LYP/6-311G(d) (Figure 3B).

Compound **3**, isolated as a white powder, had a molecular formula of C_27_H_30_N_4_O_10_. This was established from the HRESIMS data, which showed a sodium adduct ion peak at *m/z* 593.1859 [M + Na]^+^, indicating a reduction of 14 amu from **1** and 15 degrees of unsaturation. A comparison of the ^1^H NMR and ^13^C NMR data revealed similarities between **1** and **3**. The most significant difference was the substitution of the nitrogenated methine group (*δ*_C_ 86.4, C-1) in **1** with a formyl group (*δ*_C_ 198.3) in **3**, as detailed in Table 1. This alteration was corroborated by COSY correlations of H_3_-13/H-2/H-1 and HMBC correlations of H-1/C-13, H-2/C-1′, and H-2/C-4, as illustrated in Figure 2. The coupling constant ^3^*J*_H1’ − H2_ of 9.2 Hz indicated that H-1′ and H-2′ were positioned on opposite faces of the octatomic ring, which was formed by a hydrogen bond between the formyl and the amide group. In the NOESY spectrum, correlations between H_3_-13/H-1′, H_3_-13’/H-1′, and H-2/H-2′ indicated that H_3_-13, H-1′, and H_3_-13′ are located on the same side of the octatomic ring. Similarly, the positioning of H-6/6′ and H_3_-14/14′ on opposite faces of the oxirane ring was evidenced by NOESY correlations between H-8/8′ and H_3_-14/14′. Considering the biosynthetic pathway, the absolute configurations of C-2 and C-2′ were supposed to be *S*. Compound **3** was assigned the absolute configuration as 2*S*,5*S*,6*R*,1’*S*,2’*S*,5’*S*,6’*R*, which was corroborated by ECD calculations at the CAM-B3LYP/6-311G(d) level, as illustrated in Figure 3B.

**Figure 4:**
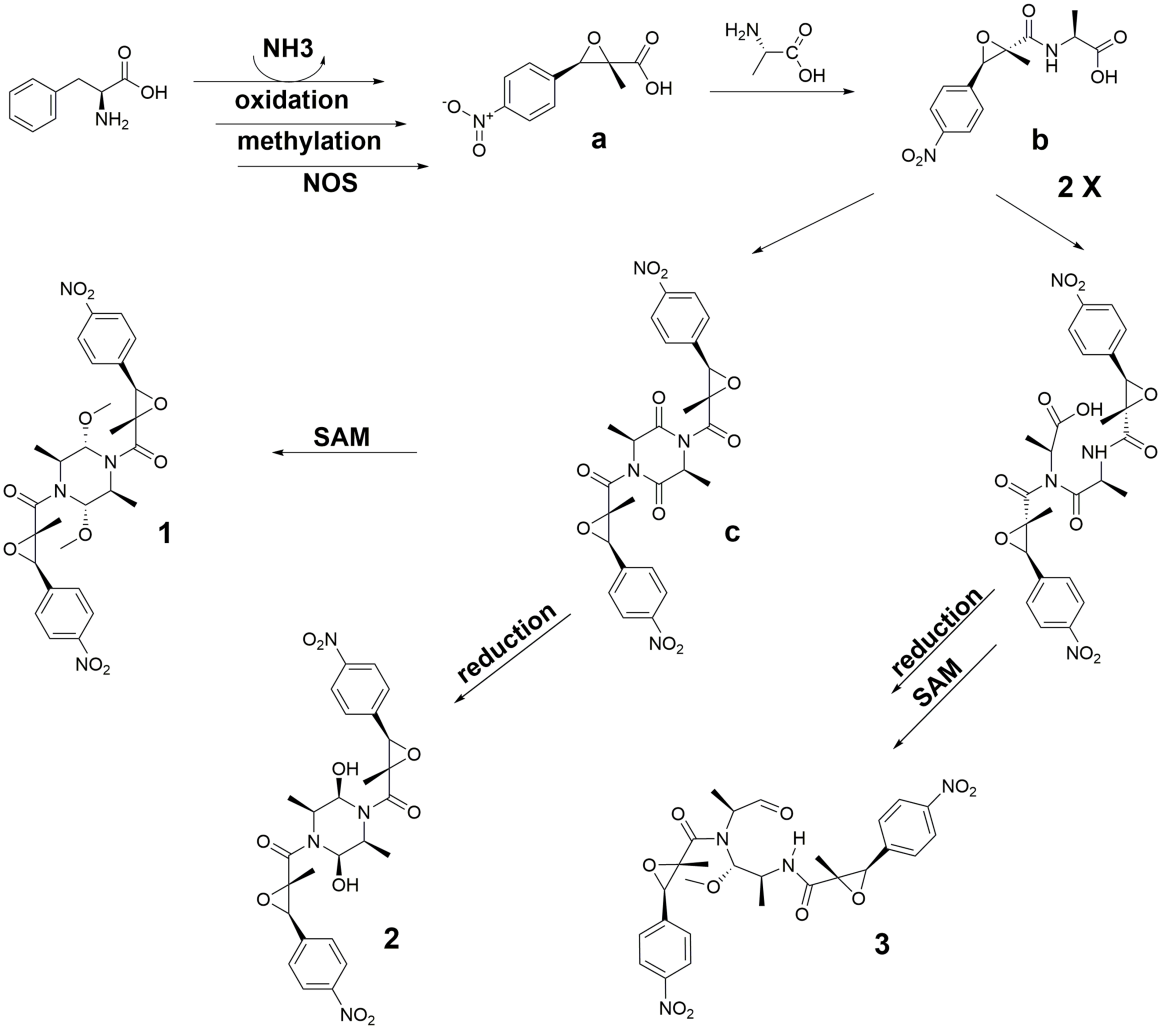
Biosynthetic pathway of **1–3**.

The biosynthetic pathway, as proposed in Figure 4, began with L-Phenylalanine undergoing a sequence of transformations: deamination, oxidation, methylation, and aryl nitration, leading to the formation of intermediate **a**. Acylation of intermediate **a** with L-Alanine resulted in intermediate **b**. Two units of **b** underwent a condensation reaction by removing two molecules of water to give intermediate **c**, which was reduced to yield **2**. Intermediate **c** underwent reduction and methylation to yield **1**. Similarly, two units of **b** condensed by eliminating one molecule of water, followed by reduction and methylation steps, resulting in **3** (Figure 4).

A comparison of NMR data with previously reported data in the literature led to the identification of the remaining fifteen known compounds, numbered **4**–**18**. They were questin (**4**) (Liu et al., 2015), ω-hydroxyemodin (**5**) (Khamthong et al., 2012), penicillixanthone (**6**) (Rukachaisirikul et al., 2014), isorhodoptilometrin (**7**) (Kim et al., 2022), emodin (**8**) (Khaliq et al., 2023), citrinin H1 (**9**) (Ngan et al., 2017), 3,5-dichloroasterric acid (**10**) (Lee et al., 2002), dihydrogeodin (**11**) (Hamed et al., 2019), dimethoxyphtalide (**12**) (Wei et al., 2020), anodendroic acid (**13**) (Prompanya et al., 2016), varioloid B (**14**) (Zhang et al., 2015), italicum (**15**) (Tomás-Barberán et al., 1990), 46-dichloro-5-methylbenzene-13-diol (**16**) (Slavov et al., 2010), 4-hydroxybenzaldehyde (**17**) (Hsu et al., 2009), and 2-phenylethanol (**18**) (Pehlivan et al., 2011).

We performed a CCK8 assay to investigate the effects of all compounds on the viability of two gastric cancer cell lines, MKN28 and MGC803. **1** exhibited a moderate inhibitory effect on the growth of MKN28 cells, with an IC_50_ value of 7.4 μM. Similarly, **7** displayed a moderate inhibitory effect on MGC803 cells, with an IC_50_ value of 2.5 μM. The remaining compounds did not achieve a 50% inhibition rate within the tested concentration range, indicating that their IC_50_ values exceeded 10 μM. Paclitaxel served as the positive control, with IC_50_ values of 0.32 and 0.7 nM for MKN28 and MGC803, respectively.

The *in vitro* antibacterial activities of compounds **1**–**18** were tested against six bacterial strains. Among them, Compound **16** showed potency against *Vibrio parahaemolyticus* ATCC 17802, exhibiting a MIC of 7.8 μg/mL. This potency was compared to the positive control, chloramphenicol, which had an MIC of 1.1 μg/mL.

## 4 Conclusion

This study details the characterization of three new compounds (**1**–**3**) and fifteen known compounds (**4**–**18**) from the deep-sea fungus *Aspergillus terreus*. Compound **1** was identified as a symmetrical dimer with a complex structure, including a p-nitrophenyl moiety, a 2-methyl-2,3-epoxyamide group, and an isopropyl fragment. Its absolute configuration was determined using DFT methods and ECD spectrum analysis. **2**, structurally similar to **1** but differing in the piperazine ring, is considered an epimer of **1**. Compound **3** shares a similar structure with **1**, but with a formyl group replacing a nitrogenated methine group. The biogenetic pathways of these compounds are proposed, involving precursors like L-phenylalanine and L-alanine. The study also reports the biological activities of these compounds. Specifically, compounds **1** and **7** exhibit moderate growth inhibitory effects on gastric cancer cell lines, while compound **16** shows moderate antibacterial properties. The study emphasizes the need for extensive research on the biological activities and potential therapeutic applications of these compounds, particularly those showing promise in preliminary tests. Developing synthetic methods for these compounds could facilitate further studies and potential pharmaceutical applications. Investigating the mechanisms behind their observed biological activities could lead to the discovery of new drug targets or therapeutic strategies.

## Data availability statement

The datasets presented in this study can be found in online repositories. The names of the repository/repositories and accession number(s) can be found in the article/Supplementary material.

## Ethics statement

Ethical approval was not required for the studies on humans in accordance with the local legislation and institutional requirements because only commercially available established cell lines were used. Ethical approval was not required for the studies on animals in accordance with the local legislation and institutional requirements because only commercially available established cell lines were used.

## Author contributions

XH: Conceptualization, Formal analysis, Funding acquisition, Writing – original draft, Writing – review & editing, Supervision. YW: Formal analysis, Writing – review & editing, Methodology. GL: Writing – review & editing, Resources. ZS: Writing – review & editing, Project administration, Investigation. JX: Data curation, Methodology, Supervision, Validation, Writing – review & editing. J-JQ: Formal analysis, Writing – review & editing. WW: Conceptualization, Formal analysis, Funding acquisition, Project administration, Writing – original draft, Writing – review & editing.
