# Supplementary material for: Secondary metabolites from the deep-sea derived fungus Aspergillus terreus MCCC M28183
Source: Front Microbiol. 2024 Feb 14;15:1361550. doi: 10.3389/fmicb.2024.1361550 (PMC10899347; doi:10.3389/fmicb.2024.1361550)
Supplement: Supplementary file 1 [file Data_Sheet_1.PDF]

Secondary Metabolites from the Deep-sea Derived Fungus *Aspergillus terreus* MCCC M28183

Xiaomei Huang[1,a], Yichao Wang[3,4,a], Guangyu Li[2], Zongze Shao[2], Jinmei Xia[2,*], Jiang-Jiang Qin[3,*], Weiyi Wang[2,*]

Table of Contents

Figure S1. ^{1}H-NMR spectrum of 1 4
Figure S2. ^{13}C-NMR spectrum of 1 4
Figure S3. HSQC spectrum of 1 5
Figure S4. HMBC spectrum of 1 5
Figure S5. COSY spectrum of 1 6
Figure S6. NOESY spectrum of 1 6
Figure S7. MS spectrum of 1 6
Figure S8. ECD spectrum of 1 7
Figure S9. ^{1}H-NMR spectrum of 2 8
Figure S10. ^{13}C-NMR spectrum of 2 8
Figure S11. HSQC spectrum of 2 9
Figure S12. HMBC spectrum of 2 9
Figure S13. COSY spectrum of 2 10
Figure S14. NOESY spectrum of 2 10
Figure S15. MS spectrum of 2 10
Figure S16. ECD spectrum of 2 11
Figure S17. ^{1}H-NMR spectrum of 3 12
Figure S18. ^{13}C-NMR spectrum of 3 12
Figure S19. HSQC spectrum of 3 13
Figure S20. HMBC spectrum of 3 13
Figure S21. COSY spectrum of 3 14
Figure S22. NOESY spectrum of 3 14
Figure S23. MS spectrum of 3 15
Figure S24. ECD spectrum of 3 15

Table S1. Conformational analysis of the B3LYP/6-31G(d) optimized conformers of 1a in the gas phase (T=298.15 K) 15
Table S2. Experimental and calculated ^{13}C-NMR and ^{1}H-NMR chemical shifts of 1a and 1b and the result of DP4+ analysis (Isomer 1: 1a; Isomer 2: 1b) 16
Table S3. Atomic coordinates (Å) of 1a-1 obtained at the B3LYP/6-31G(d) level of theory in the gas phase. 17
Table S4. Atomic coordinates (Å) of 1a-2 obtained at the B3LYP/6-31G(d) level of theory in the gas phase. 17
Table S5. Atomic coordinates (Å) of 1a-3 obtained at the B3LYP/6-31G(d) level of theory in the gas phase. 18
Table S6. Atomic coordinates (Å) of 1a-4 obtained at the B3LYP/6-31G(d) level of theory in the gas phase. 19
Table S7. Atomic coordinates (Å) of 1a-5 obtained at the B3LYP/6-31G(d) level of theory in the gas phase. 20
Table S8. Conformational analysis of the B3LYP/6-31G(d) optimized conformers of 1b in the gas phase (T=298.15 K) 20
Table S9. Atomic coordinates (Å) of 1b-1 obtained at the B3LYP/6-31G(d) level of theory in the gas phase. 21
Table S10. Atomic coordinates (Å) of 1b-2 obtained at the B3LYP/6-31G(d) level of theory in the gas phase. 21
Table S11. Key transitions, oscillator strengths, and rotatory strengths in the ECD spectrum of conformer 1a-1 at the CAM-B3LYP/6-311G(d) level of theory in MeOH with IEFPCM solvent model. 22
Table S12. Key transitions, oscillator strengths, and rotatory strengths in the ECD spectrum of conformer 1a-2 at the CAM-B3LYP/6-311G(d) level of theory in MeOH with IEFPCM solvent model. 24
Table S13. Key transitions, oscillator strengths, and rotatory strengths in the ECD spectrum of conformer 1a-3 at the CAM-B3LYP/6-311G(d) level of theory in MeOH with IEFPCM solvent model. 26
Table S14. Key transitions, oscillator strengths, and rotatory strengths in the ECD spectrum of conformer 1a-4 at the CAM-B3LYP/6-311G(d) level of theory in MeOH with IEFPCM solvent model. 27
Table S15. Key transitions, oscillator strengths, and rotatory strengths in the ECD spectrum of conformer 1a-5 at the CAM-B3LYP/6-311G(d) level of theory in MeOH with IEFPCM solvent model. 29
Figure S25. Optimized geometries of 5 dominant conformers of 1a (1a-1 to 1a-5, respectively) at the B3LYP/6-31G(d) level of theory in the gas phase 31
Table S16. Conformational analysis of the B3LYP/6-31G(d) optimized conformers of 2 in the gas phase (T=298.15 K) 31

Table S17. Atomic coordinates (Å) of 2-1 obtained at the B3LYP/6-31G(d) level of theory in the gas phase. 31
Table S18. Atomic coordinates (Å) of 2-2 obtained at the B3LYP/6-31G(d) level of theory in the gas phase. 32
Table S19. Atomic coordinates (Å) of 2-3 obtained at the B3LYP/6-31G(d) level of theory in the gas phase. 33
Table S20. Atomic coordinates (Å) of 2-4 obtained at the B3LYP/6-31G(d) level of theory in the gas phase. 33
Table S21. Key transitions, oscillator strengths, and rotatory strengths in the ECD spectrum of conformer 2-1 at the CAM-B3LYP/6-311G(d) level of theory in MeOH with IEFPCM solvent model. 34
Table S22. Key transitions, oscillator strengths, and rotatory strengths in the ECD spectrum of conformer 2-2 at the CAM-B3LYP/6-311G(d) level of theory in MeOH with IEFPCM solvent model. 36
Table S23. Key transitions, oscillator strengths, and rotatory strengths in the ECD spectrum of conformer 2-3 at the CAM-B3LYP/6-311G(d) level of theory in MeOH with IEFPCM solvent model. 37
Table S24. Key transitions, oscillator strengths, and rotatory strengths in the ECD spectrum of conformer 2-4 at the CAM-B3LYP/6-311G(d) level of theory in MeOH with IEFPCM solvent model. 38
Table S25. Conformational analysis of the B3LYP/6-31G(d) optimized conformers of 3 in the gas phase (T=298.15 K) 40
Table S26. Atomic coordinates (Å) of 3-1 obtained at the B3LYP/6-31G(d) level of theory in the gas phase. 40
Table S27. Atomic coordinates (Å) of 3-2 obtained at the B3LYP/6-31G(d) level of theory in the gas phase. 41
Table S28. Key transitions, oscillator strengths, and rotatory strengths in the ECD spectrum of conformer 3-1 at the CAM-B3LYP/6-311G(d) level of theory in MeOH with IEFPCM solvent model. 42
Table S29. Key transitions, oscillator strengths, and rotatory strengths in the ECD spectrum of conformer 3-2 at the CAM-B3LYP/6-311G(d) level of theory in MeOH with IEFPCM solvent model. 44
Figure S26. Fractionaion Tree 46
General experimental procedures 46

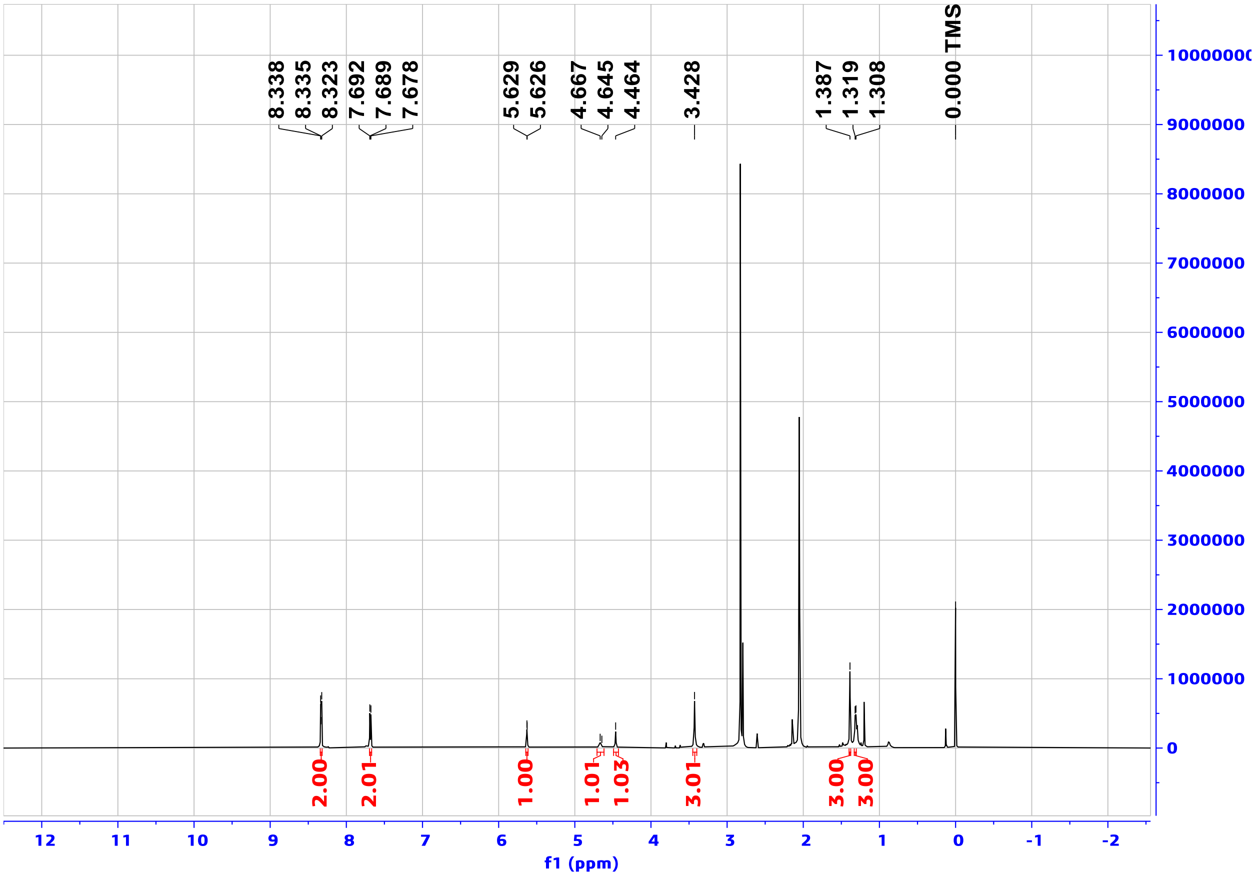

Figure S1. ^{1}H-NMR spectrum of **1**

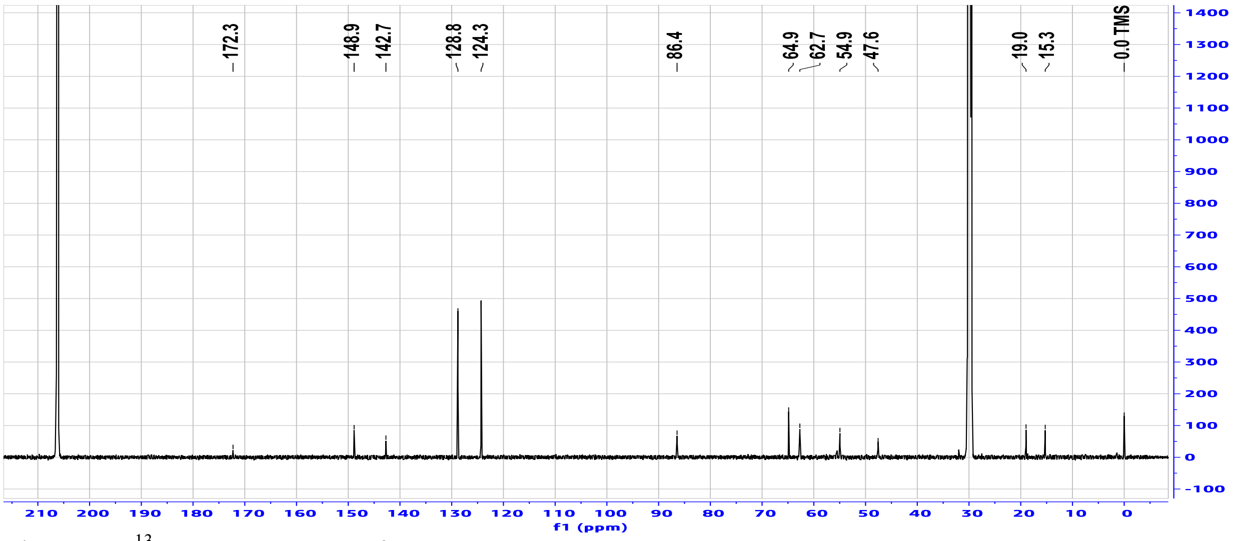

Figure S2. ^{13}C-NMR spectrum of **1**

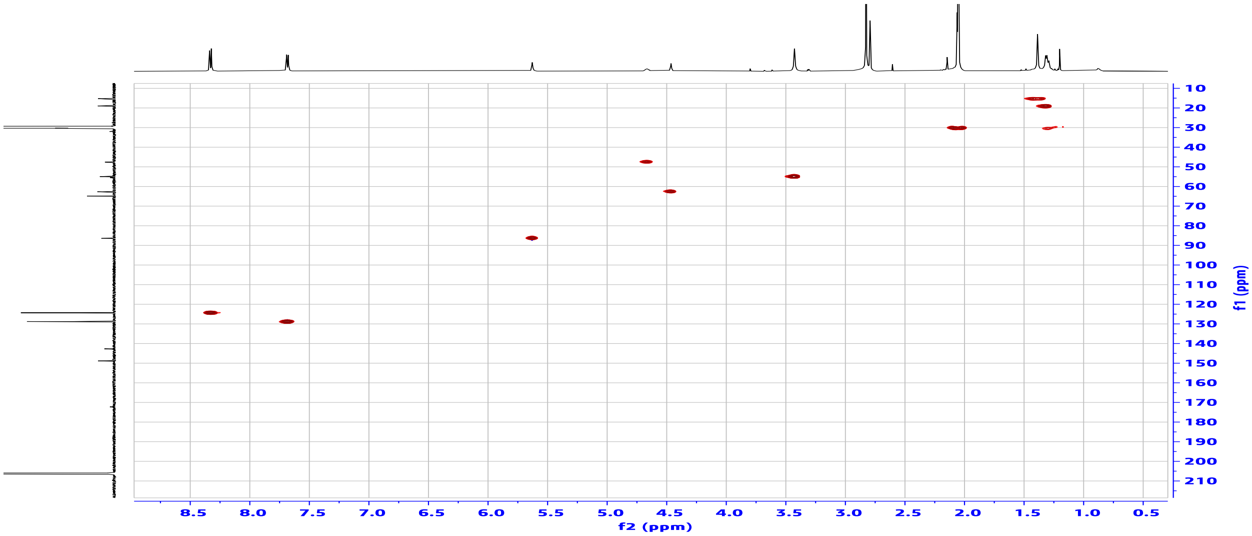

Figure S3. HSQC spectrum of **1**

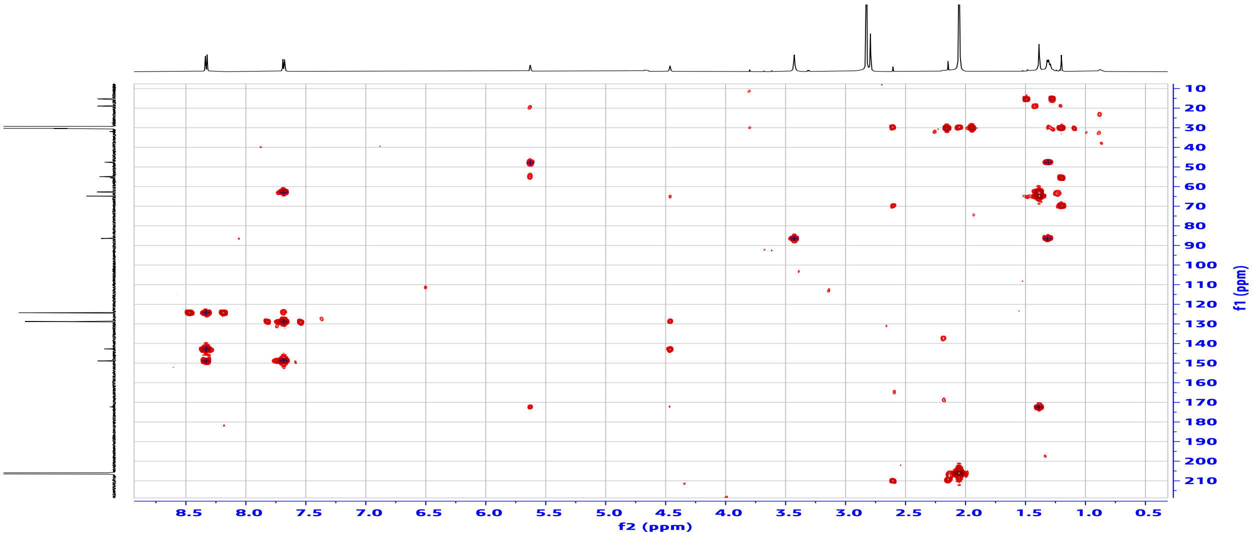

Figure S4. HMBC spectrum of **1**

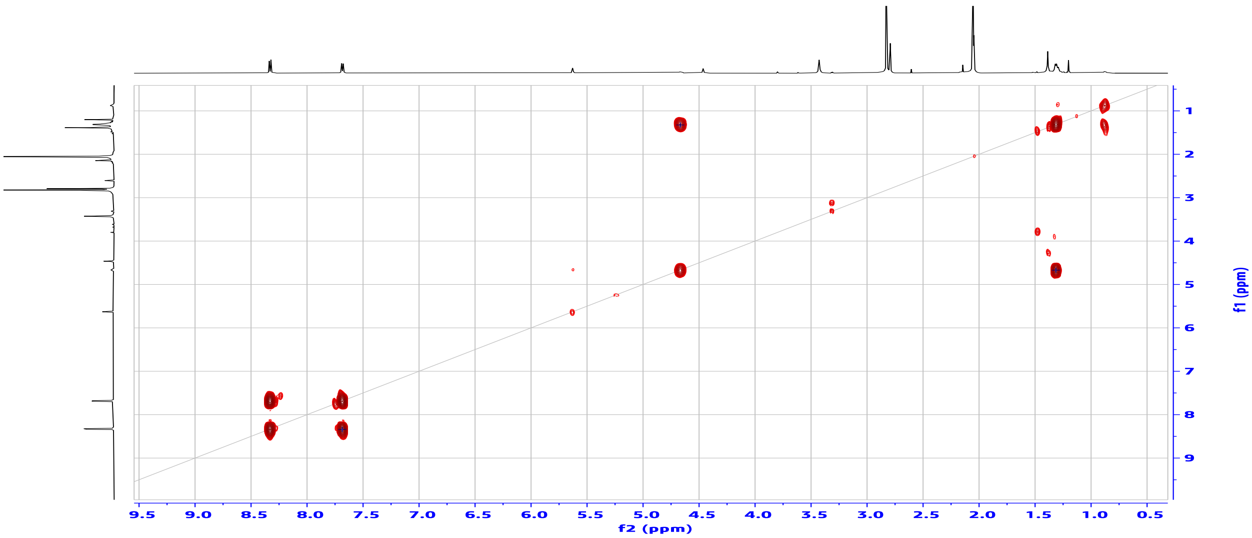

Figure S5. COSY spectrum of **1**

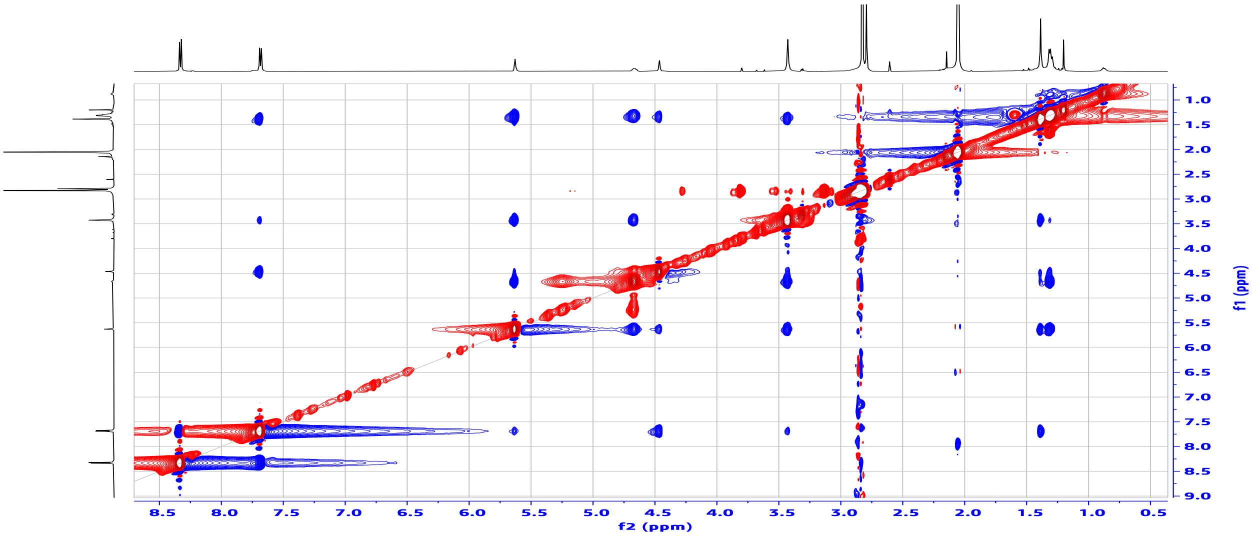

Figure S6. NOESY spectrum of **1**

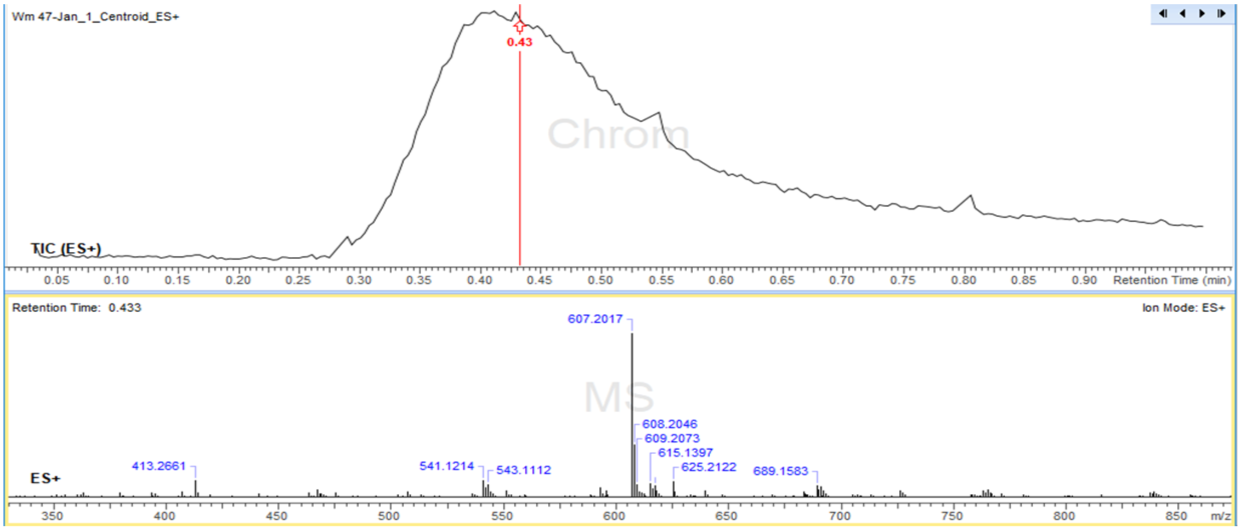

Figure S7. MS spectrum of **1**

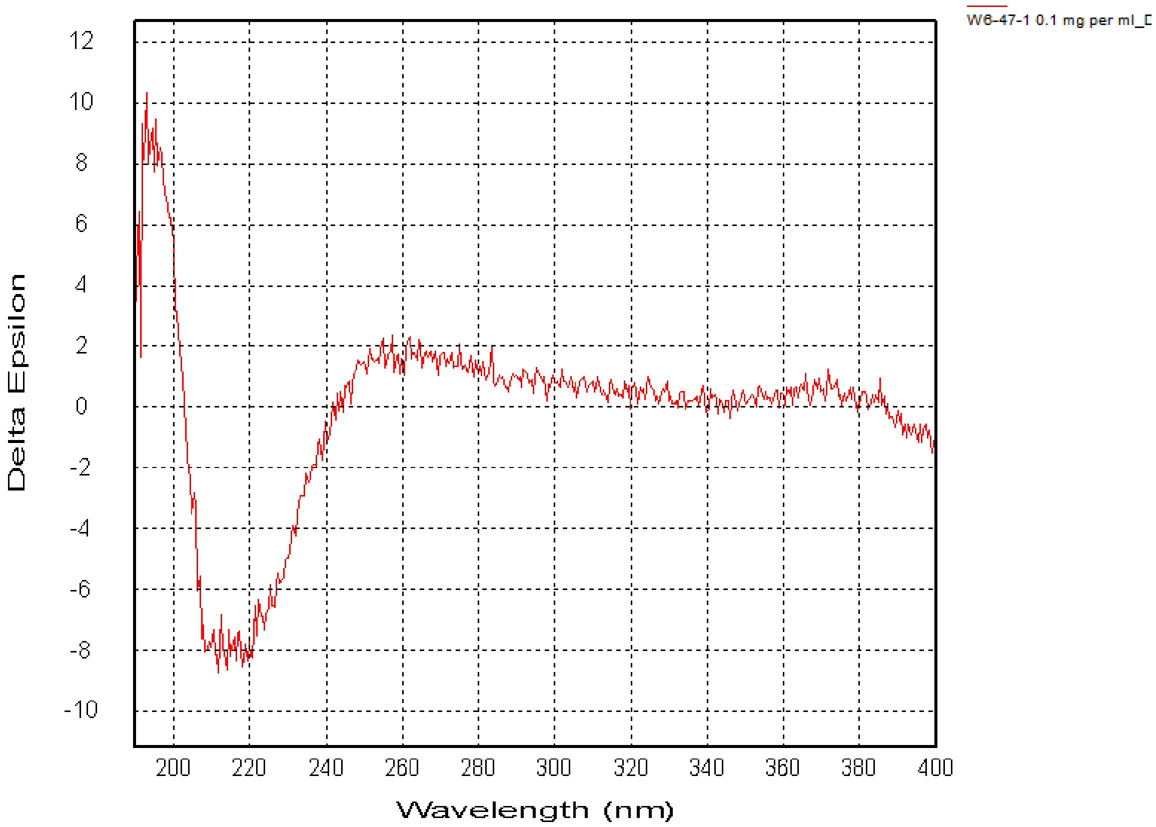

Figure S8. ECD spectrum of **1**

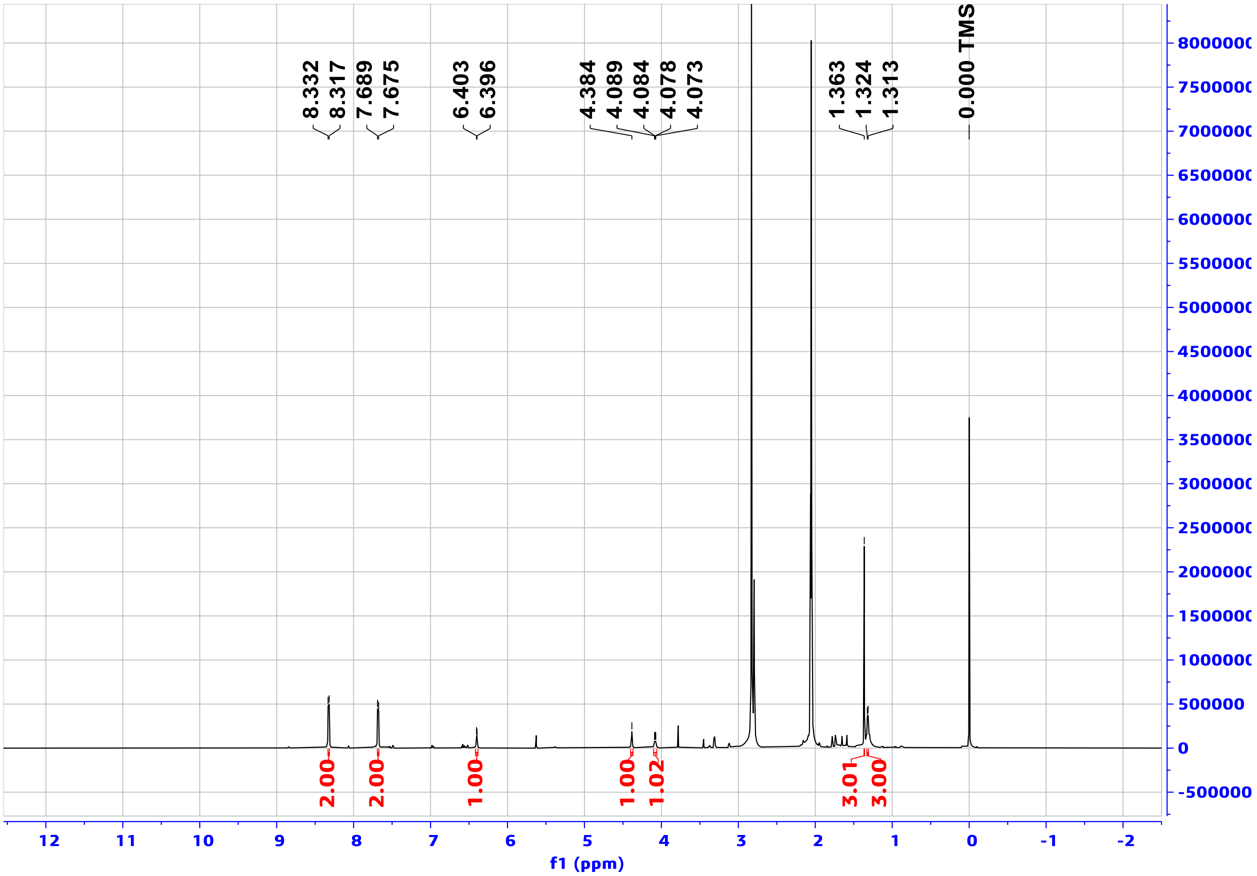

Figure S9. ^{1}H-NMR spectrum of **2**

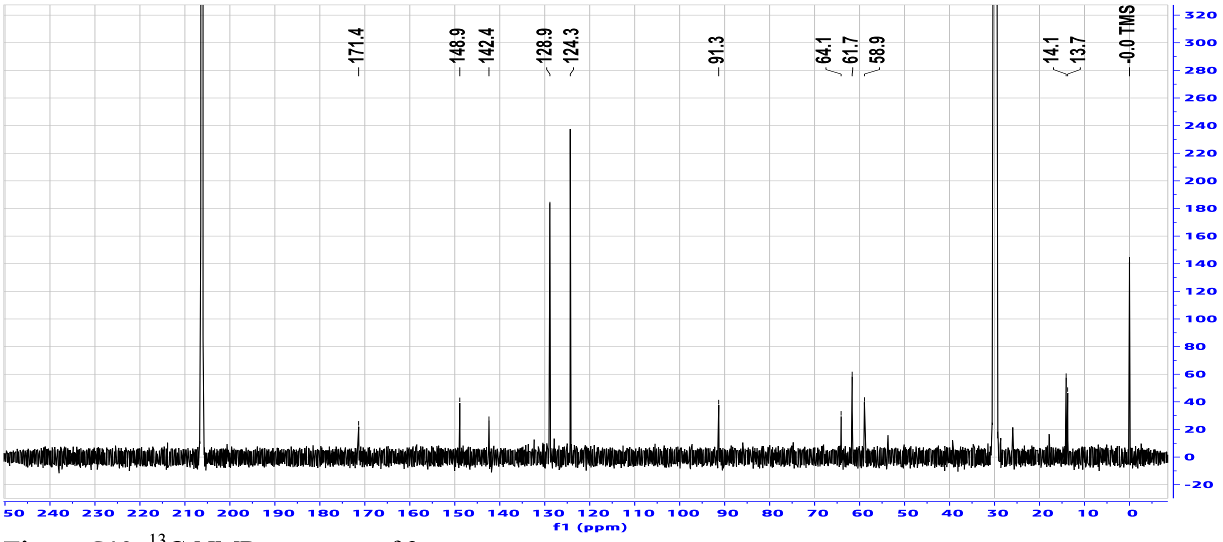

Figure S10. ^{13}C-NMR spectrum of **2**

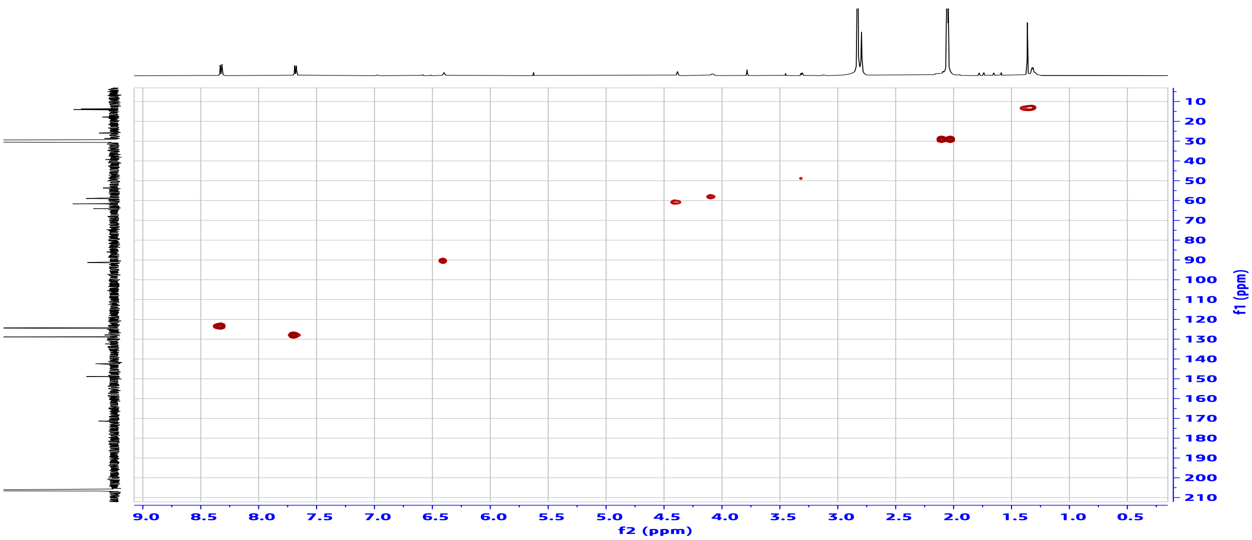

Figure S11. HSQC spectrum of **2**

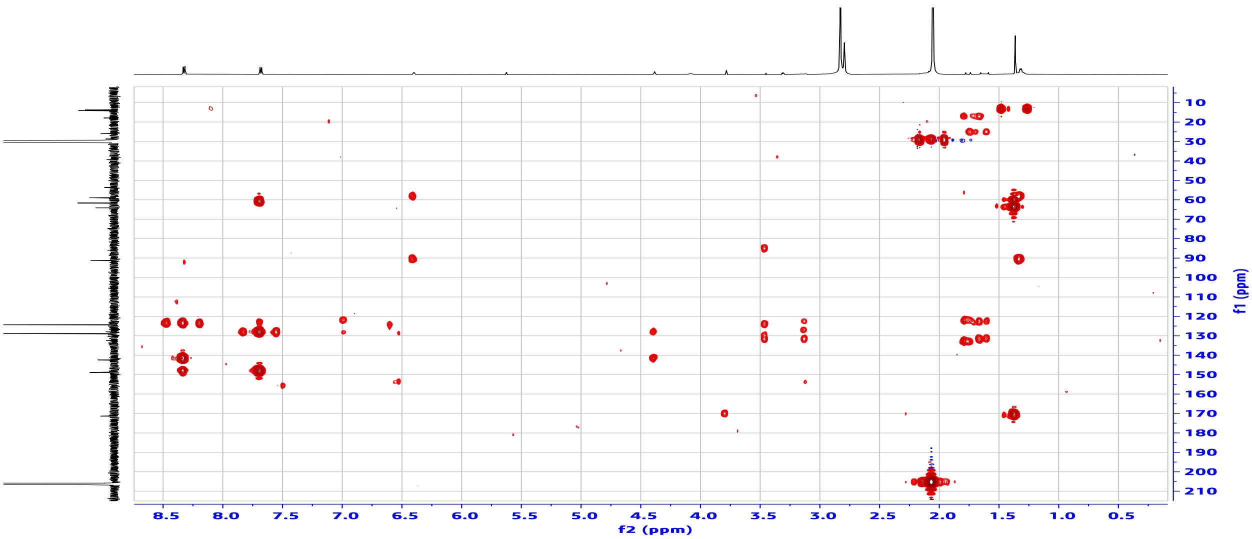

Figure S12. HMBC spectrum of **2**

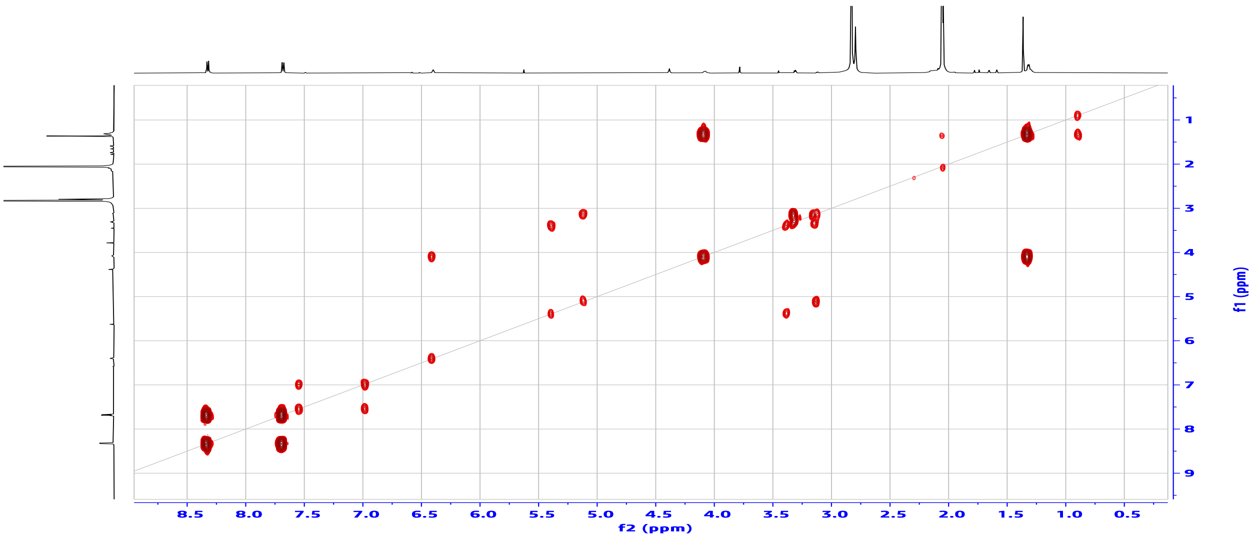

Figure S13. COSY spectrum of **2**

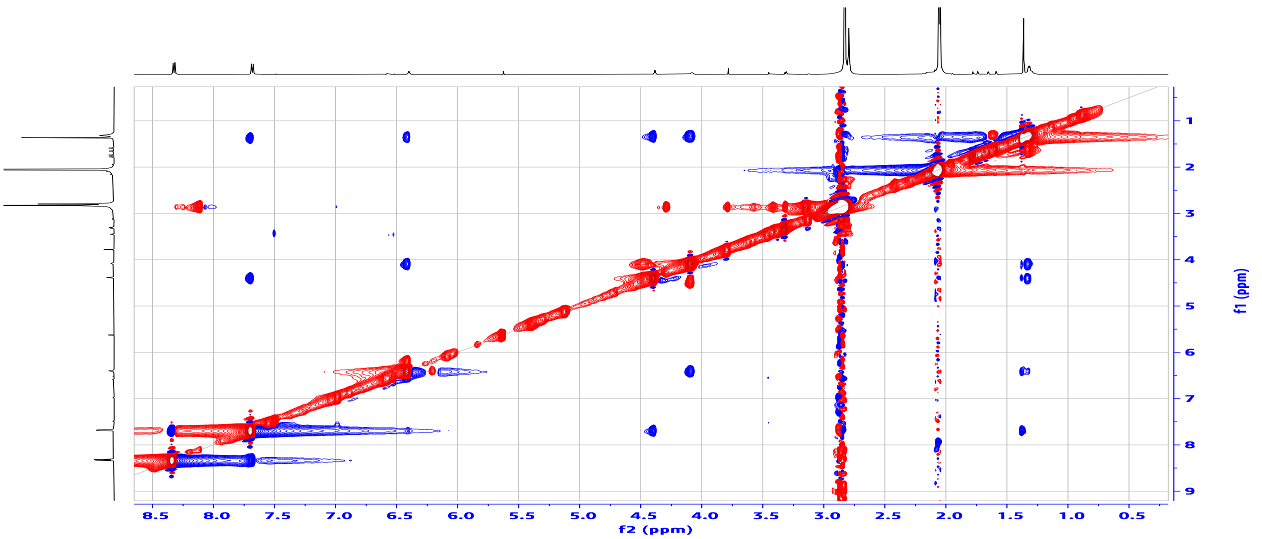

Figure S14. NOESY spectrum of **2**

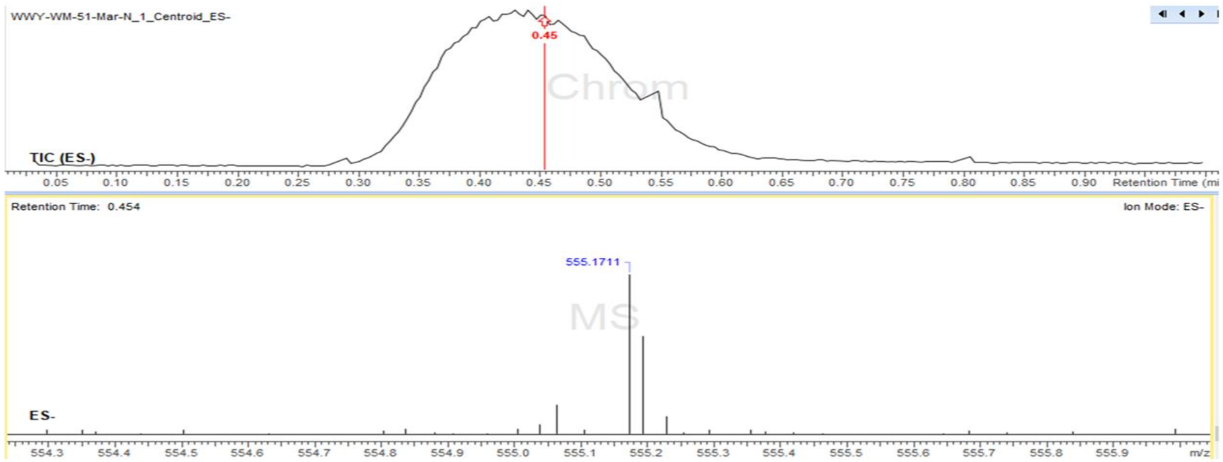

Figure S15. MS spectrum of **2**

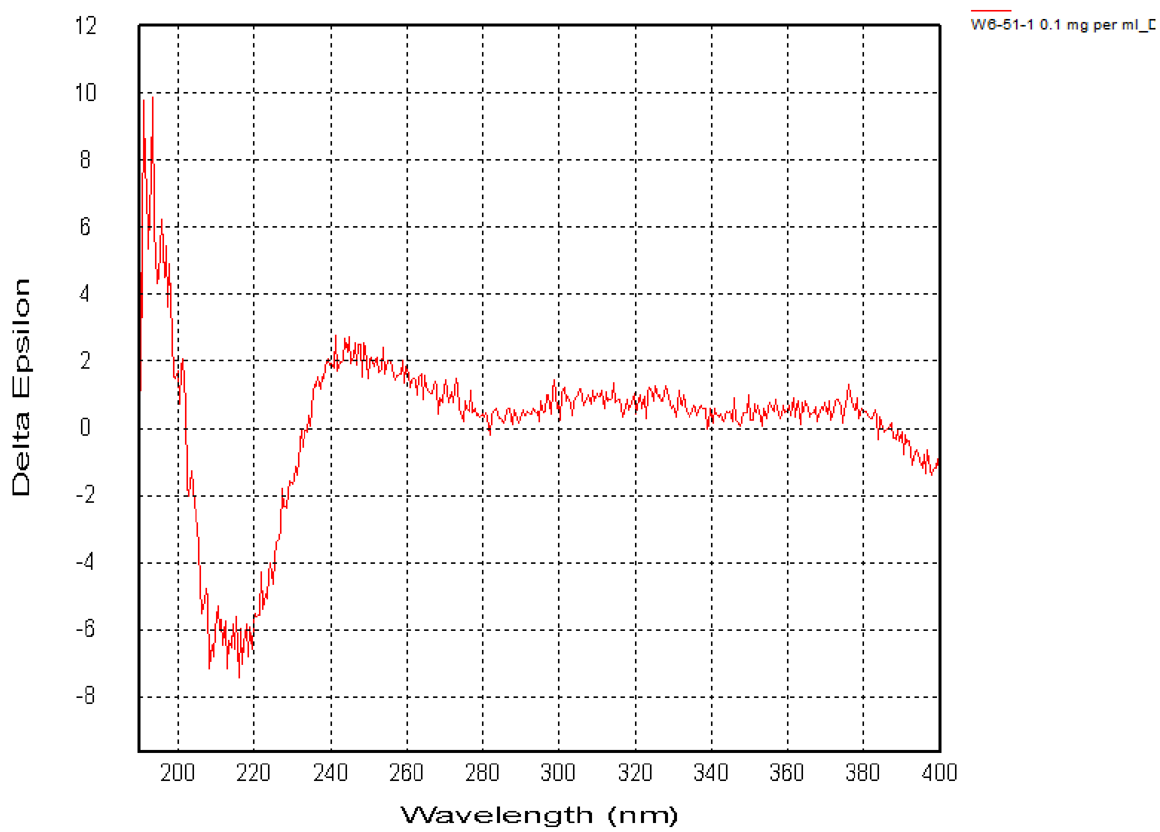

Figure S16. ECD spectrum of **2**

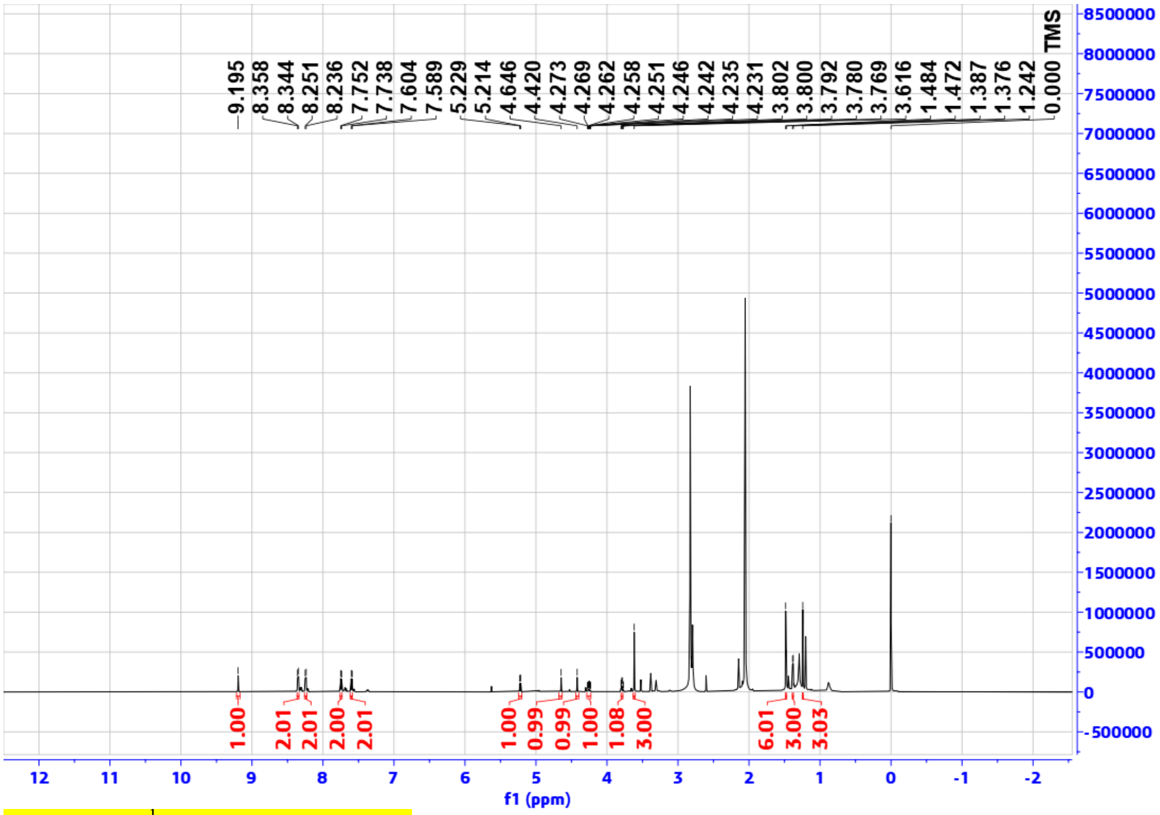

Figure S17. ^{1}H-NMR spectrum of **3**

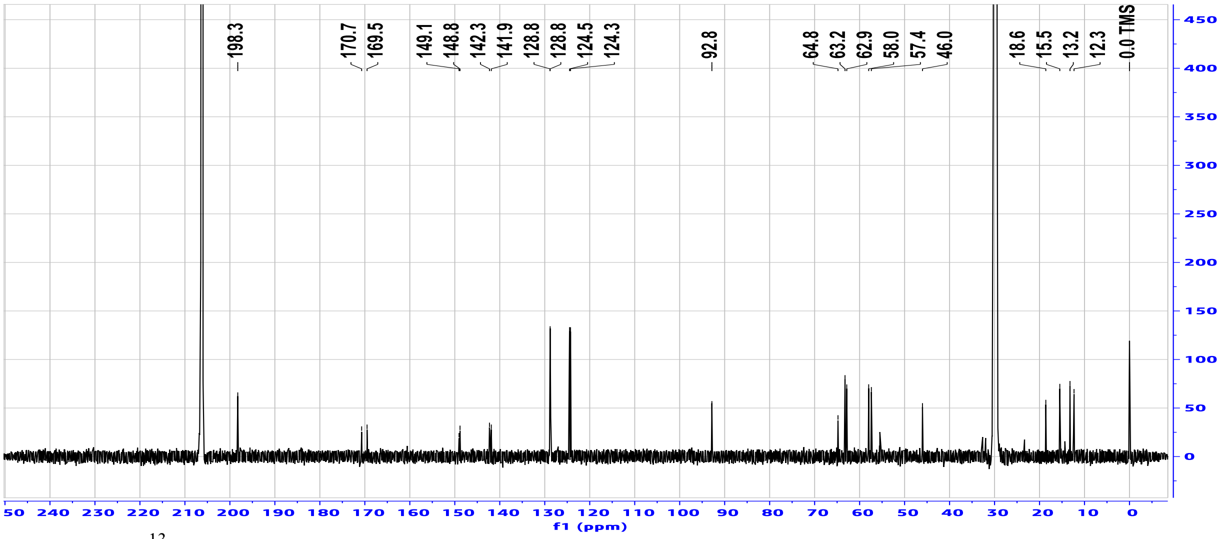

Figure S18. ^{13}C-NMR spectrum of **3**

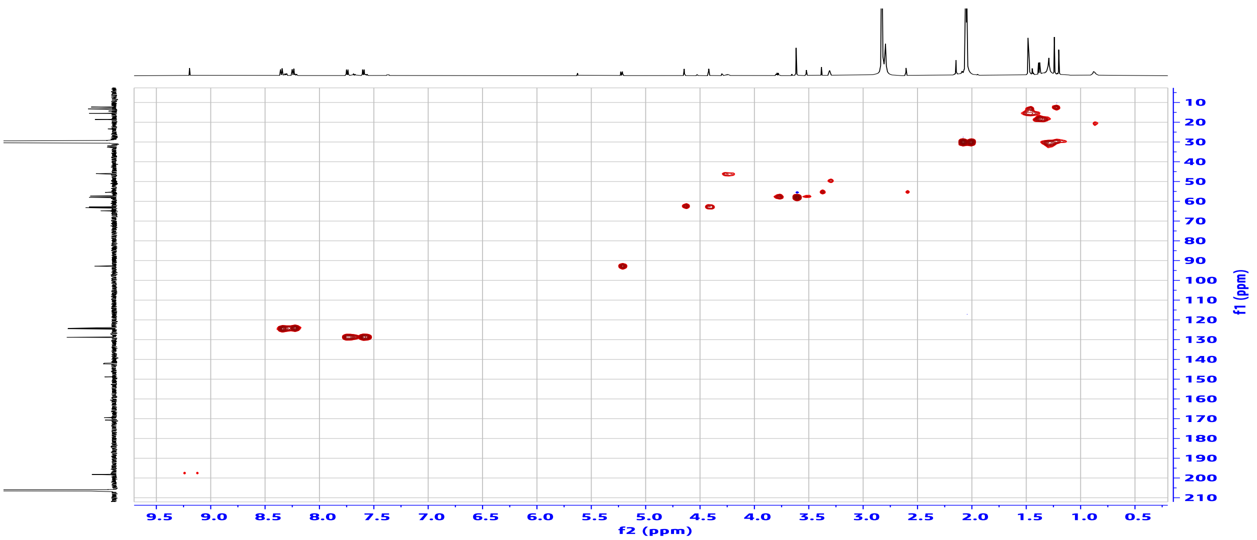

Figure S19. HSQC spectrum of **3**

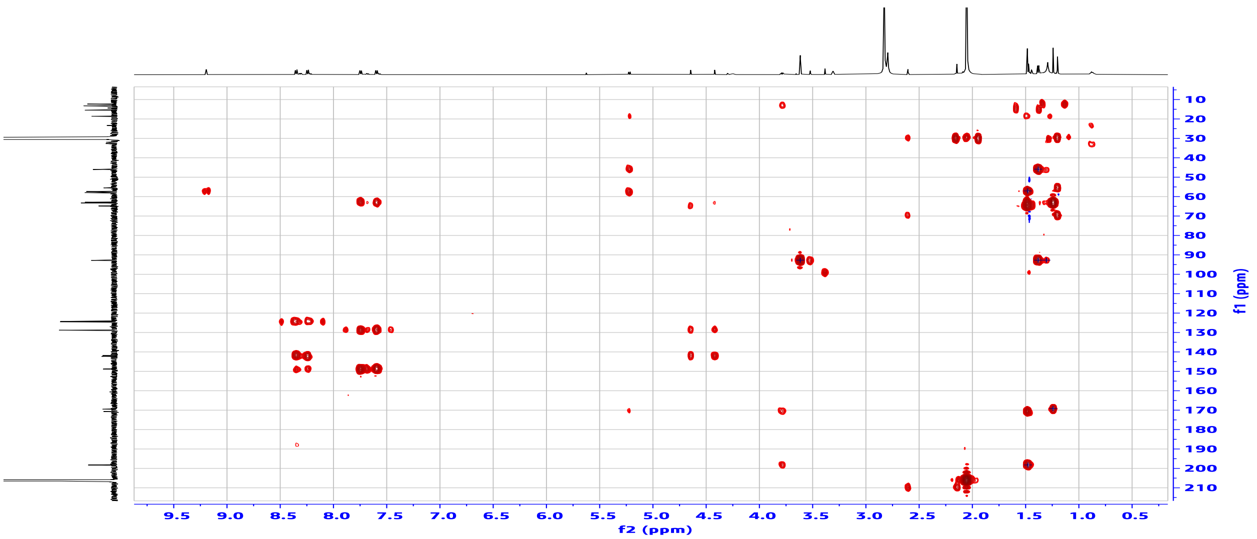

Figure S20. HMBC spectrum of **3**

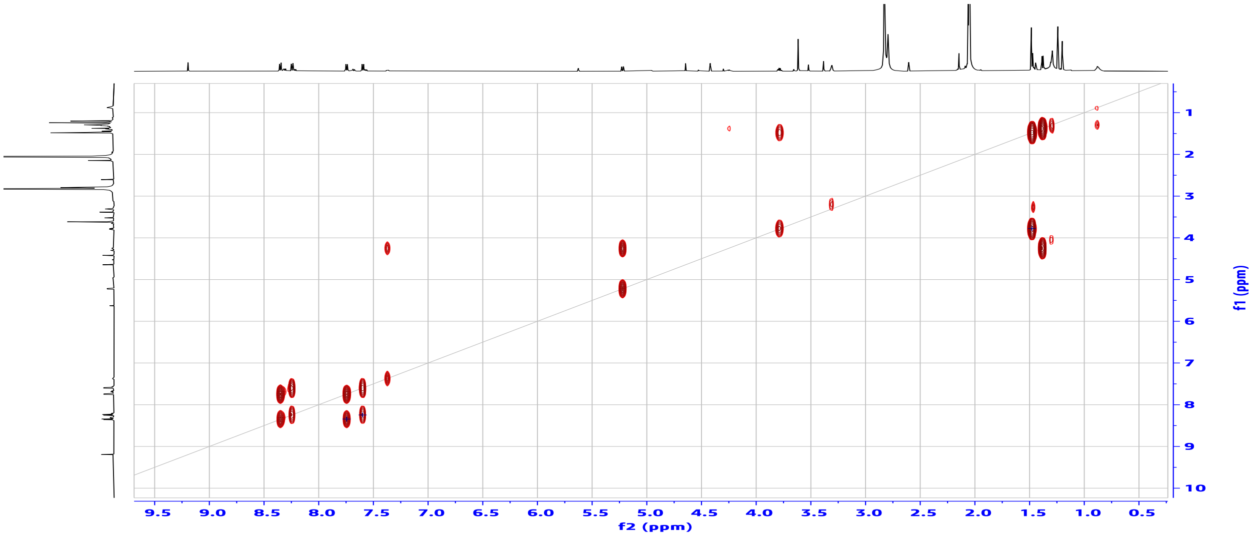

Figure S21. COSY spectrum of **3**

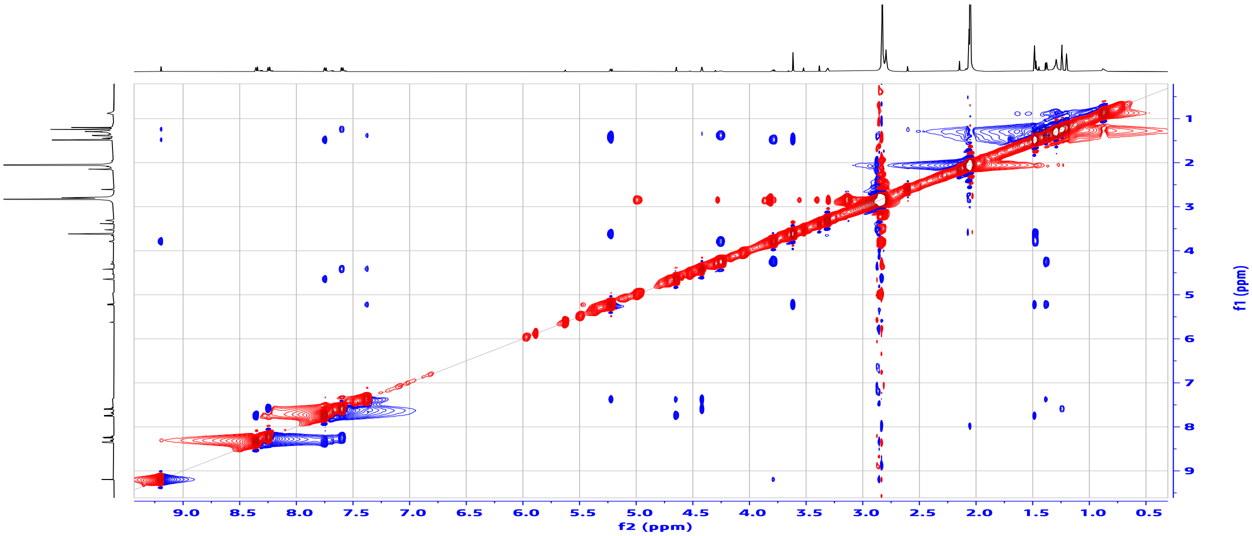

Figure S22. NOESY spectrum of **3**

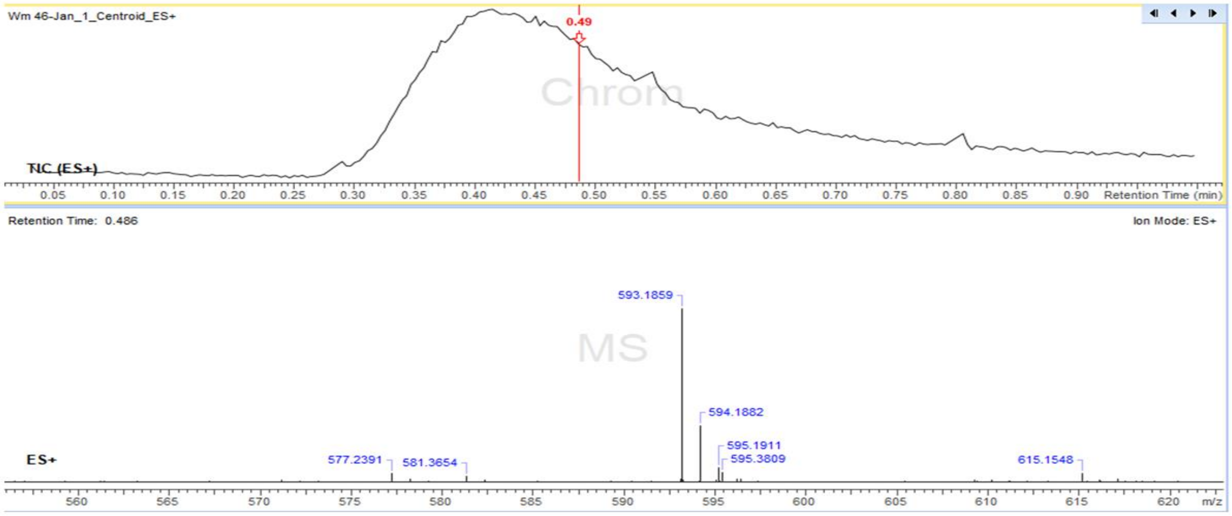

Figure S23. MS spectrum of **3**

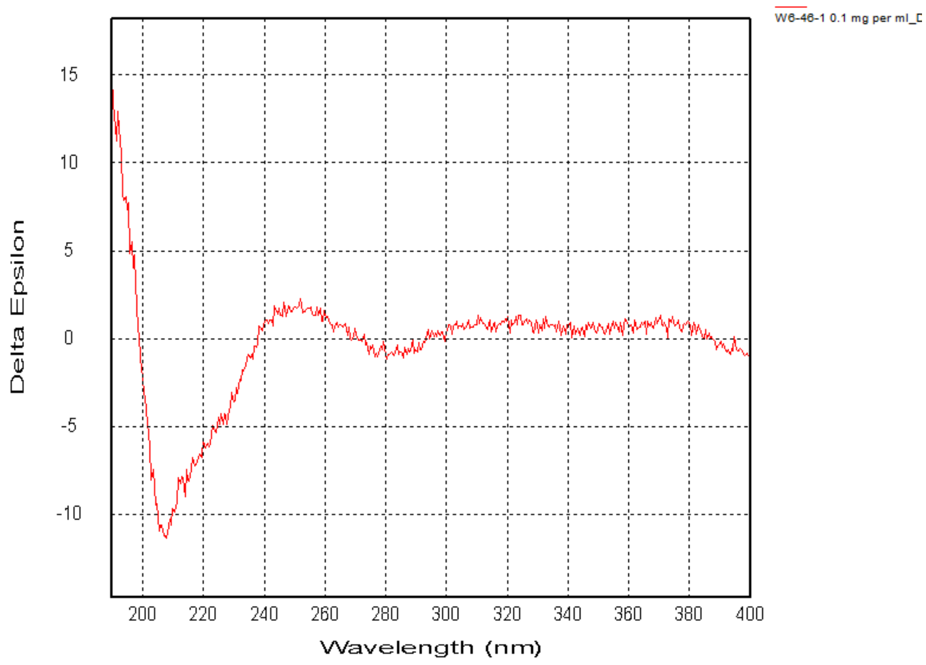

Figure S24. ECD spectrum of **3**

Table S1. Conformational analysis of the B3LYP/6-31G(d) optimized conformers of 1a in the gas phase (T=298.15 K)

Conformer	E[a] (Hartree)	C[b] (Hartree)	G[c] (kcal/mol)	ΔG[d] (kcal/mol)	Population[e]
1a-1	-2057.065863	0.500687	-1290494.648122	0.0	56.16%
1a-2	-2057.064834	0.501195	-1290493.683749	0.964373	11.02%
1a-3	-2057.064834	0.501195	-1290493.683686	0.964436	11.02%
1a-4	-2057.064834	0.501204	-1290493.677806	0.970316	10.91%
1a-5	-2057.064834	0.501204	-1290493.677781	0.970341	10.91%

[a]Electronic energy obtained at M062X/6-311+G(2d,p) level of theory; [b]Thermal correction to Gibbs free energy obtained at B3LYP/6-31G(d) level of theory; [c]Gibbs free energy (E + C); [d]The relative Gibbs free energy; [e]The Boltzmann distribution of each conformer.

Table S2. Experimental and calculated ^{13}C-NMR and ^{1}H-NMR chemical shifts of 1a and 1b and the result of DP4+ analysis (Isomer 1: 1a; Isomer 2: 1b)

Functional		Solvent?		Basis Set		Type of Data	
mPW1PW91		PCM		6-31+G(d,p)		Unscaled Shifts	
		DP4+	100.00%	0.00%	–	–	–
Nuclei	sp2?	Experimental	Isomer 1	Isomer 2	Isomer 3	Isomer 4	Isomer 5
C	|	86.4	85.1	86.7			
C	|	47.6	47.8	48.2			
C	x	172.3	169.4	169.5			
C	|	65	68.2	67.1			
C	|	62.7	64.2	63.3			
C	x	142.7	143.3	142.9			
C	x	128.8	124.7	124.3			
C	x	124.3	122.8	122.9			
C	x	148.9	145.6	147.9			
C	x	124.3	122.4	122.1			
C	x	128.8	125.9	125.1			
C	|	19.2	19.2	20			
C	|	15.3	15.7	13.6			
C	|	54.9	53.9	54.2			
C	|	86.4	85.1	86.8			
C	|	47.6	47.8	48			
C	x	172.3	169.4	169.6			
C	|	65	68.2	66.9			
C	|	62.7	64.2	63.3			
C	x	142.7	143.3	142.9			
C	x	128.8	125.9	125.1			
C	x	124.3	122.4	122.1			
C	x	148.9	145.6	147.9			
C	x	124.3	122.8	122.9			
C	x	128.8	124.7	124.4			
C	|	19.2	19.2	19.9			
C	|	15.3	15.7	13.7			
C	|	54.9	53.9	54.3			
H	|	5.63	5.52	4.83			
H	|	4.66	4.65	4.66			
H	|	4.46	4.44	4.32			
H	x	7.68	7.68	7.76			
H	x	8.33	8.76	8.73			
H	x	8.33	8.67	8.91			
H	x	7.68	8.02	8.03			
H	|	1.31	1.15	1.4			
H	|	1.39	1.22	1.22			
H	|	3.43	3.38	2.93			
H	|	5.63	5.52	4.79			
H	|	4.66	4.65	4.69			
H	|	4.46	4.44	4.34			
H	x	7.68	8.02	8.04			
H	x	8.33	8.67	8.9			
H	x	8.33	8.76	8.73			
H	x	7.68	7.68	7.77			
H	|	1.31	1.15	1.39			
H	|	1.39	1.22	1.22			
H	|	3.43	3.38	2.94			

Table S3. Atomic coordinates (Å) of 1a-1 obtained at the B3LYP/6-31G(d) level of theory in the gas phase.

C	-1.104736	0.868890	0.054322	N	-10.413442	0.084222	0.190820
C	0.189691	1.421044	-0.548911	O	-10.861585	1.066398	0.781813
N	1.327670	0.507332	-0.339240	O	-11.087642	-0.873040	-0.188453
C	2.554138	1.108210	-0.219116	O	-1.131101	0.932917	1.476139
C	3.774686	0.292359	0.188649	O	-3.969777	1.009272	-0.402479
C	4.789834	0.076067	-0.867367	H	9.085353	1.858484	-1.050406
C	6.249734	0.010854	-0.589829	H	6.629271	1.920112	-1.519326
C	6.814916	-1.085051	0.072787	H	6.173452	-1.905824	0.373094
C	8.181275	-1.122615	0.331745	H	8.642878	-1.959952	0.839474
C	8.969891	-0.051653	-0.080799	H	4.530259	0.432270	-1.865318
C	8.433860	1.049093	-0.746875	H	4.276132	1.525198	1.856940
C	7.068082	1.071241	-1.002686	H	3.319756	0.085467	2.266317
C	0.011297	1.804744	-2.022137	H	5.057338	-0.073888	1.884934
C	4.136538	0.460902	1.644658	H	0.436149	2.335941	-0.005325
C	-1.425980	2.225846	1.977464	H	1.922461	-1.475592	-0.340603
C	1.104744	-0.868840	0.054327	H	-0.704872	2.629494	-2.105489
C	-0.189681	-1.420989	-0.548916	H	-0.363310	0.971270	-2.620878
N	-1.327662	-0.507271	-0.339273	H	0.965845	2.142444	-2.431239
C	-2.554132	-1.108148	-0.219179	H	-9.085346	-1.858623	-1.050244
C	-3.774688	-0.292297	0.188566	H	-6.629274	-1.920223	-1.519223
C	-4.789866	-0.076089	-0.867439	H	-6.173500	1.905854	0.372921
C	-6.249760	-0.010891	-0.589868	H	-8.642915	1.959955	0.839359
C	-7.068093	-1.071327	-1.002631	H	-4.530307	-0.432342	-1.865377
C	-8.433865	-1.049195	-0.746786	H	-4.276085	-1.525041	1.856941
C	-8.969905	0.051585	-0.080773	H	-3.319697	-0.085286	2.266211
C	-8.181305	1.122595	0.331676	H	-5.057290	0.074046	1.884867
C	-6.814951	1.085046	0.072686	H	-0.436147	-2.335880	-0.005322
C	-0.011264	-1.804704	-2.022136	H	-1.922454	1.475650	-0.340592
C	-4.136497	-0.460756	1.644595	H	0.363375	-0.971241	-2.620873
C	1.426034	-2.225850	1.977419	H	0.704889	-2.629469	-2.105466
O	3.969733	-1.009250	-0.402322	H	-0.965809	-2.142387	-2.431258
O	-2.714221	-2.313340	-0.389580	H	1.531080	-2.122110	3.059437
N	10.413433	-0.084307	0.190759	H	0.622973	-2.947204	1.771702
O	10.861566	-1.066444	0.781824	H	2.366388	-2.611681	1.555766
O	11.087645	0.872919	-0.188586	H	-1.531086	2.122059	3.059471
O	1.131108	-0.932913	1.476142	H	-0.622868	2.947165	1.771823
O	2.714235	2.313399	-0.389530	H	-2.366291	2.611751	1.555784

Table S4. Atomic coordinates (Å) of 1a-2 obtained at the B3LYP/6-31G(d) level of theory in the gas phase.

C	-0.866445	1.378762	0.461062	N	-9.851994	-1.179461	0.057561
C	0.162539	2.360226	-0.117757	O	-10.484766	-0.375073	0.740933
N	1.486103	1.737883	-0.311186	O	-10.326001	-2.197925	-0.444937
C	2.559988	2.602650	-0.368501	O	-0.721929	1.066026	1.843926
C	3.929399	1.978881	-0.616039	O	-3.711611	1.033331	-0.258307
C	4.643402	1.378156	0.542718	H	5.913237	-3.056348	1.138955
C	5.616569	0.265794	0.395042	H	4.336546	-1.128559	1.420445
C	6.847276	0.450522	-0.247028	H	7.105099	1.437996	-0.613145
C	7.729359	-0.615022	-0.396139	H	8.687834	-0.497277	-0.885609
C	7.364136	-1.862658	0.104284	H	4.107547	1.395969	1.490950
C	6.150925	-2.071145	0.758389	H	3.380443	0.951570	-2.441202

C	5.281914	-0.997045	0.904504	H	4.206335	2.501255	-2.666836
C	-0.366240	2.965093	-1.424533	H	5.132738	1.068938	-2.167597
C	4.180733	1.591906	-2.057321	H	0.297307	3.178684	0.592864
C	-0.778536	2.180411	2.717400	H	2.533651	-0.031915	-0.591554
C	1.640304	0.323317	-0.066151	H	-1.260378	3.564660	-1.224100
C	0.460925	-0.447893	-0.673396	H	-0.634915	2.188376	-2.145681
N	-0.834041	0.122764	-0.256306	H	0.391367	3.619100	-1.859660
C	-1.901036	-0.739857	-0.275049	H	-8.165178	-2.678866	-1.351077
C	-3.264987	-0.257927	0.202236	H	-5.740413	-2.215822	-1.754730
C	-4.297108	-0.137220	-0.852769	H	-6.045088	1.386808	0.559677
C	-5.743151	-0.387648	-0.609420	H	-8.481393	0.914341	0.959119
C	-6.338019	-1.531331	-1.159404	H	-3.965603	-0.330287	-1.873936
C	-7.684106	-1.799867	-0.941657	H	-3.470455	-1.728083	1.737101
C	-8.427165	-0.904561	-0.174364	H	-2.890245	-0.148978	2.305425
C	-7.863362	0.243583	0.375913	H	-4.611718	-0.371244	1.883965
C	-6.513706	0.497586	0.153595	H	0.484031	-1.476165	-0.308527
C	0.585787	-0.504669	-2.200200	H	-1.844392	1.842283	0.312669
C	-3.587277	-0.645436	1.624887	H	0.661999	0.494956	-2.635022
C	1.588145	-1.119677	1.888367	H	1.477259	-1.075211	-2.483492
O	4.994545	2.669093	0.037040	H	-0.285080	-1.014412	-2.616816
O	-1.797128	-1.900812	-0.657881	H	1.972824	-1.087412	2.910454
N	8.287598	-2.993423	-0.056239	H	0.512305	-1.321803	1.924359
O	9.354032	-2.783356	-0.633289	H	2.097541	-1.929593	1.345015
O	7.937911	-4.084524	0.394277	H	-0.941596	1.779711	3.720390
O	1.858834	0.156591	1.332641	H	0.158547	2.751333	2.713895
O	2.423087	3.814042	-0.302707	H	-1.611185	2.852935	2.461332

Table S5. Atomic coordinates (Å) of 1a-3 obtained at the B3LYP/6-31G(d) level of theory in the gas phase.

C	1.640304	0.323316	-0.066150	N	8.287599	-2.993421	-0.056240
C	0.460925	-0.447895	-0.673393	O	9.354034	-2.783353	-0.633291
N	-0.834041	0.122762	-0.256304	O	7.937913	-4.084522	0.394278
C	-1.901037	-0.739858	-0.275048	O	1.858835	0.156591	1.332642
C	-3.264988	-0.257928	0.202235	O	4.994544	2.669093	0.037038
C	-4.297109	-0.137223	-0.852770	H	-8.165179	-2.678869	-1.351071
C	-5.743151	-0.387649	-0.609421	H	-5.740414	-2.215827	-1.754725
C	-6.513706	0.497587	0.153591	H	-6.045088	1.386810	0.559670
C	-7.863362	0.243586	0.375909	H	-8.481393	0.914346	0.959113
C	-8.427166	-0.904559	-0.174365	H	-3.965603	-0.330293	-1.873936
C	-7.684107	-1.799868	-0.941655	H	-3.470457	-1.728079	1.737104
C	-6.338020	-1.531333	-1.159402	H	-2.890246	-0.148973	2.305424
C	0.585787	-0.504673	-2.200197	H	-4.611719	-0.371239	1.883964
C	-3.587279	-0.645432	1.624887	H	0.484032	-1.476167	-0.308523
C	1.588147	-1.119676	1.888369	H	-1.844392	1.842280	0.312672
C	-0.866445	1.378760	0.461063	H	1.477259	-1.075215	-2.483489
C	0.162537	2.360224	-0.117757	H	0.661999	0.494952	-2.635020
N	1.486102	1.737881	-0.311186	H	-0.285080	-1.014417	-2.616812
C	2.559986	2.602650	-0.368502	H	5.913240	-3.056346	1.138956
C	3.929398	1.978881	-0.616040	H	4.336548	-1.128558	1.420446
C	4.643402	1.378157	0.542717	H	7.105098	1.437998	-0.613148
C	5.616569	0.265795	0.395041	H	8.687834	-0.497275	-0.885611
C	5.281915	-0.997044	0.904504	H	4.107547	1.395970	1.490949

C	6.150927	-2.071143	0.758389	H	3.380442	0.951568	-2.441202
C	7.364137	-1.862656	0.104283	H	4.206333	2.501254	-2.666838
C	7.729359	-0.615020	-0.396141	H	5.132737	1.068937	-2.167598
C	6.847276	0.450523	-0.247029	H	0.297306	3.178683	0.592864
C	-0.366242	2.965090	-1.424532	H	2.533651	-0.031916	-0.591553
C	4.180732	1.591905	-2.057322	H	-0.634917	2.188372	-2.145680
C	-0.778529	2.180409	2.717402	H	-1.260380	3.564656	-1.224100
O	-3.711611	1.033330	-0.258312	H	0.391364	3.619097	-1.859661
O	2.423085	3.814041	-0.302708	H	-0.941589	1.779709	3.720392
N	-9.851995	-1.179459	0.057561	H	0.158556	2.751328	2.713895
O	-10.484767	-0.375067	0.740930	H	-1.611176	2.852936	2.461335
O	-10.326002	-2.197924	-0.444933	H	1.972826	-1.087410	2.910456
O	-0.721928	1.066024	1.843927	H	0.512307	-1.321803	1.924363
O	-1.797128	-1.900814	-0.657881	H	2.097543	-1.929593	1.345018

Table S6. Atomic coordinates (Å) of 1a-4 obtained at the B3LYP/6-31G(d) level of theory in the gas phase.

C	-0.866347	1.379341	0.460484	N	-9.851656	-1.180257	0.057723
C	0.162931	2.360754	-0.117919	O	-10.325531	-2.198535	-0.445272
N	1.486444	1.738273	-0.311268	O	-10.484504	-0.376344	0.741584
C	2.560453	2.602909	-0.368167	O	-0.722257	1.066667	1.843399
C	3.929830	1.979006	-0.615546	O	-3.711635	1.033696	-0.257542
C	4.643468	1.377872	0.543221	H	5.912343	-3.057027	1.138525
C	5.616475	0.265378	0.395476	H	4.335927	-1.129027	1.420147
C	6.847378	0.450064	-0.246229	H	7.105469	1.437593	-0.612009
C	7.729305	-0.615599	-0.395414	H	8.687926	-0.497891	-0.884607
C	7.363726	-1.863309	0.104565	H	4.107409	1.395535	1.491340
C	6.150309	-2.071760	0.758298	H	3.381140	0.952275	-2.441117
C	5.281456	-0.997543	0.904493	H	4.207354	2.501877	-2.666137
C	-0.365509	2.966095	-1.424616	H	5.133390	1.069268	-2.167057
C	4.181448	1.592370	-2.056871	H	0.297701	3.178984	0.592971
C	-0.781188	2.180978	2.716815	H	2.533803	-0.031601	-0.591903
C	1.640394	0.323616	-0.066593	H	-1.259507	3.565857	-1.224137
C	0.461020	-0.447223	-0.674263	H	-0.634309	2.189639	-2.146003
N	-0.833894	0.123347	-0.256909	H	0.392318	3.620012	-1.859494
C	-1.900831	-0.739353	-0.275562	H	-8.481271	0.913188	0.960450
C	-3.264744	-0.257760	0.202163	H	-6.045091	1.386293	0.561016
C	-4.297041	-0.136583	-0.852623	H	-5.740125	-2.214833	-1.755698
C	-5.743010	-0.387382	-0.609235	H	-8.164767	-2.678511	-1.352052
C	-6.513612	0.497248	0.154434	H	-3.965651	-0.328983	-1.873953
C	-7.863202	0.242894	0.376750	H	-3.469822	-1.728872	1.736159
C	-8.426892	-0.904989	-0.174187	H	-2.889670	-0.150046	2.305339
C	-7.683785	-1.799695	-0.942132	H	-4.611189	-0.372228	1.884052
C	-6.337766	-1.530809	-1.159870	H	0.484017	-1.475650	-0.309832
C	0.585912	-0.503395	-2.201081	H	-1.844210	1.842955	0.311811
C	-3.586769	-0.646171	1.624627	H	0.662193	0.496390	-2.635515
C	1.587376	-1.119885	1.887542	H	1.477357	-1.073879	-2.484579
O	4.994936	2.668881	0.037956	H	-0.284977	-1.012925	-2.617916
O	-1.796903	-1.900237	-0.658614	H	1.971645	-1.087902	2.909791
N	8.287026	-2.994198	-0.056035	H	0.511487	-1.321836	1.923065
O	9.353650	-2.784165	-0.632749	H	2.096840	-1.929786	1.344229
O	7.937030	-4.085360	0.394094	H	-0.944146	1.780054	3.719731

O	1.858530	0.156450	1.332201	H	0.154924	2.753504	2.713841
O	2.423703	3.814306	-0.302134	H	-1.614805	2.852106	2.460217

Table S7. Atomic coordinates (Å) of 1a-5 obtained at the B3LYP/6-31G(d) level of theory in the gas phase.

C	1.640394	0.323615	-0.066593	N	8.287028	-2.994196	-0.056035
C	0.461020	-0.447224	-0.674263	O	9.353651	-2.784163	-0.632749
N	-0.833894	0.123346	-0.256909	O	7.937032	-4.085359	0.394094
C	-1.900831	-0.739353	-0.275562	O	1.858530	0.156450	1.332201
C	-3.264745	-0.257760	0.202162	O	4.994935	2.668881	0.037956
C	-4.297042	-0.136583	-0.852623	H	-8.481271	0.913191	0.960446
C	-5.743010	-0.387382	-0.609235	H	-6.045092	1.386295	0.561012
C	-6.337767	-1.530811	-1.159869	H	-5.740125	-2.214835	-1.755695
C	-7.683786	-1.799696	-0.942130	H	-8.164767	-2.678513	-1.352048
C	-8.426892	-0.904989	-0.174187	H	-3.965651	-0.328985	-1.873954
C	-7.863203	0.242896	0.376748	H	-3.469824	-1.728871	1.736160
C	-6.513613	0.497249	0.154432	H	-2.889670	-0.150045	2.305338
C	0.585912	-0.503395	-2.201080	H	-4.611189	-0.372226	1.884052
C	-3.586770	-0.646170	1.624626	H	0.484017	-1.475650	-0.309832
C	1.587377	-1.119886	1.887542	H	-1.844210	1.842954	0.311812
C	-0.866347	1.379340	0.460485	H	1.477356	-1.073880	-2.484579
C	0.162931	2.360753	-0.117918	H	0.662192	0.496390	-2.635514
N	1.486443	1.738273	-0.311268	H	-0.284977	-1.012926	-2.617915
C	2.560453	2.602909	-0.368167	H	5.912345	-3.057026	1.138526
C	3.929829	1.979006	-0.615546	H	4.335928	-1.129027	1.420148
C	4.643468	1.377872	0.543221	H	7.105469	1.437594	-0.612010
C	5.616475	0.265379	0.395475	H	8.687927	-0.497890	-0.884608
C	5.281457	-0.997542	0.904494	H	4.107409	1.395535	1.491340
C	6.150310	-2.071759	0.758298	H	3.381139	0.952275	-2.441118
C	7.363727	-1.863307	0.104565	H	4.207353	2.501877	-2.666138
C	7.729305	-0.615598	-0.395415	H	5.133389	1.069267	-2.167057
C	6.847378	0.450065	-0.246230	H	0.297701	3.178983	0.592971
C	-0.365510	2.966094	-1.424616	H	2.533803	-0.031602	-0.591903
C	4.181447	1.592370	-2.056871	H	-0.634310	2.189639	-2.146002
C	-0.781186	2.180976	2.716816	H	-1.259508	3.565856	-1.224136
O	-3.711635	1.033696	-0.257544	H	0.392317	3.620011	-1.859493
O	2.423702	3.814305	-0.302133	H	-0.944143	1.780053	3.719732
N	-9.851656	-1.180255	0.057723	H	0.154927	2.753501	2.713841
O	-10.325532	-2.198535	-0.445269	H	-1.614802	2.852106	2.460219
O	-10.484505	-0.376342	0.741583	H	1.971647	-1.087903	2.909791
O	-0.722257	1.066666	1.843400	H	0.511488	-1.321837	1.923066
O	-1.796904	-1.900238	-0.658615	H	2.096841	-1.929786	1.344228

Table S8. Conformational analysis of the B3LYP/6-31G(d) optimized conformers of 1b in the gas phase (T=298.15 K)

Conformer	E[a] (Hartree)	C[b] (Hartree)	G[c] (kcal/mol)	ΔG[d] (kcal/mol)	Population[e]
1b-1	-2057.060178	0.501272	-1290490.713245	0.0	95.08%
1b-2	-2057.057854	0.501742	-1290488.960004	1.753241	4.92%

[a]Electronic energy obtained at M062X/6-311+G(2d,p) level of theory; [b]Thermal correction to Gibbs free energy obtained at B3LYP/6-31G(d) level of theory; [c]Gibbs free energy (E + C); [d]The relative Gibbs free energy; [e]The Boltzmann distribution of each conformer.

Table S9. Atomic coordinates (Å) of 1b-1 obtained at the B3LYP/6-31G(d) level of theory in the gas phase.

C	1.384894	-0.128879	0.303129	N	8.794883	-1.227171	0.194814
C	0.610142	-1.303181	-0.306007	O	8.805966	-2.451557	0.069778
N	-0.845163	-1.131894	-0.148342	O	9.749543	-0.552219	0.577750
C	-1.593358	-2.291114	-0.199133	O	1.414233	-0.132900	1.723030
C	-3.098950	-2.176442	0.017804	O	3.855777	2.918838	-0.943605
C	-3.909158	-1.494806	-1.030784	H	-6.539133	2.333446	-0.677521
C	-5.195039	-0.809245	-0.738415	H	-4.420412	1.141021	-1.249869
C	-6.317335	-1.527169	-0.306602	H	-6.250411	-2.606223	-0.226330
C	-7.501973	-0.864951	-0.001453	H	-8.383065	-1.398384	0.332072
C	-7.549161	0.520699	-0.134327	H	-3.356538	-1.082066	-1.878333
C	-6.453468	1.259014	-0.577086	H	-3.322278	-3.341954	1.784598
C	-5.278687	0.583112	-0.884758	H	-2.944626	-1.634909	2.087913
C	1.006517	-1.519226	-1.771960	H	-4.589599	-2.110818	1.585420
C	-3.524552	-2.315795	1.460595	H	0.876196	-2.210077	0.241780
C	2.242365	-1.141688	2.277615	H	-2.415648	0.215297	-0.057411
C	-1.384904	0.128863	0.303128	H	2.054964	-1.833825	-1.832272
C	-0.610156	1.303141	-0.306058	H	0.888373	-0.606713	-2.362395
N	0.845148	1.131861	-0.148380	H	0.396254	-2.313176	-2.205631
C	1.593340	2.291083	-0.199185	H	6.539177	-2.333416	-0.677623
C	3.098929	2.176418	0.017776	H	4.420457	-1.140996	-1.249987
C	3.909165	1.494818	-1.030816	H	6.250383	2.606233	-0.226260
C	5.195047	0.809262	-0.738440	H	8.383036	1.398399	0.332158
C	5.278715	-0.583090	-0.884832	H	3.356569	1.082099	-1.878390
C	6.453496	-1.258989	-0.577151	H	3.322204	3.341875	1.784613
C	7.549169	-0.520677	-0.134335	H	2.944574	1.634811	2.087860
C	7.501960	0.864967	-0.001412	H	4.589548	2.110765	1.585415
C	6.317323	1.527183	-0.306570	H	-0.876212	2.210060	0.241689
C	-1.006527	1.519123	-1.772022	H	2.415634	-0.215325	-0.057419
C	3.524501	2.315730	1.460579	H	-0.888375	0.606587	-2.362420
C	-2.242292	1.141796	2.277585	H	-2.054976	1.833713	-1.832351
O	-3.855785	-2.918829	-0.943611	H	-0.396267	2.313059	-2.205721
O	1.082174	3.380791	-0.407734	H	-1.816116	2.146216	2.155486
N	-8.794876	1.227195	0.194812	H	-3.248307	1.126033	1.828861
O	-9.749557	0.552236	0.577682	H	-2.323031	0.923927	3.344234
O	-8.805942	2.451584	0.069803	H	2.323084	-0.923788	3.344260
O	-1.414224	0.132940	1.723028	H	1.816255	-2.146139	2.155540
O	-1.082193	-3.380826	-0.407669	H	3.248381	-1.125870	1.828897

Table S10. Atomic coordinates (Å) of 1b-2 obtained at the B3LYP/6-31G(d) level of theory in the gas phase.

C	1.627792	0.229855	-0.496673	N	8.626813	-2.826931	0.373120
C	0.345216	-0.439115	-1.047944	O	9.792082	-2.451493	0.500195
N	-0.926234	0.193694	-0.643864	O	8.253498	-3.995771	0.474496
C	-2.041123	-0.602873	-0.733061	O	2.212702	-0.482688	0.575125
C	-3.421170	0.013732	-0.520999	O	4.957237	2.503033	-0.537719
C	-4.247865	-0.580655	0.549877	H	-8.611172	1.110892	0.499061
C	-5.726524	-0.714419	0.455664	H	-6.119812	1.382303	0.694572
C	-6.284185	-1.987707	0.276544	H	-5.634427	-2.856031	0.214976
C	-7.660668	-2.144985	0.166494	H	-8.114952	-3.116807	0.021753
C	-8.469987	-1.013099	0.246642	H	-3.755139	-1.331105	1.169008
C	-7.942730	0.261764	0.434731	H	-3.417680	1.416832	-2.152737

C	-6.562796	0.405000	0.539317	H	-3.991007	-0.208922	-2.573415
C	0.426618	-0.570110	-2.576353	H	-5.039987	0.891472	-1.648206
C	-4.009475	0.564943	-1.799743	H	0.337140	-1.451796	-0.637514
C	1.465720	-0.607551	1.782217	H	-1.892345	1.963254	-0.147287
C	-0.953777	1.445621	0.078714	H	1.264121	-1.222908	-2.844789
C	0.191638	2.353763	-0.353057	H	0.581232	0.398710	-3.059359
N	1.483507	1.661087	-0.233801	H	-0.487830	-1.022622	-2.962472
C	2.561019	2.479223	0.048820	H	9.044603	-0.215908	0.080722
C	3.943387	1.874379	0.269677	H	7.299773	1.526958	-0.412518
C	4.653904	1.130092	-0.791229	H	4.275390	-1.518302	-0.417024
C	5.692662	0.106356	-0.476547	H	6.020540	-3.249489	0.056115
C	7.031399	0.480399	-0.319629	H	4.138424	0.973564	-1.741157
C	8.001417	-0.478176	-0.041689	H	4.189480	2.695515	2.222536
C	7.609343	-1.808794	0.079578	H	3.628599	1.006087	2.216357
C	6.280192	-2.205144	-0.061368	H	5.324463	1.378656	1.842804
C	5.321351	-1.238593	-0.340879	H	0.240799	3.176882	0.361729
C	-0.033065	2.973552	-1.736327	H	2.375018	0.098758	-1.278257
C	4.294567	1.723221	1.731738	H	-0.164293	2.217386	-2.513919
C	-1.406788	2.187000	2.298441	H	-0.930162	3.603031	-1.721365
O	-3.700367	0.740901	0.689224	H	0.818455	3.607795	-1.992528
O	2.424820	3.686665	0.198310	H	-0.769788	3.081854	2.274560
N	-9.926694	-1.170726	0.132352	H	-2.431597	2.455840	2.014207
O	-10.367025	-2.309096	-0.024007	H	-1.409947	1.787089	3.314547
O	-10.617656	-0.154546	0.198940	H	2.081435	-1.221526	2.442769
O	-0.909962	1.150710	1.464962	H	1.276583	0.367506	2.237477
O	-1.988568	-1.769042	-1.108089	H	0.501827	-1.100304	1.615456

Table S11. Key transitions, oscillator strengths, and rotatory strengths in the ECD spectrum of conformer 1a-1 at the CAM-B3LYP/6-311G(d) level of theory in MeOH with IEFPCM solvent model.

Num[a]	*Transition*[b]	*CI-coeff*[c]	*ΔE (eV)*[d]	*λ (nm)*[e]	*f*[f]	R_{vel}[g]	R_{len}[h]
1	143->155	0.3058	3.9934	310.48	0.0000	-3.2831	-3.3672
	143->156	0.34838					
	144->155	0.36139					
	144->156	0.28913					
2	143->155	0.34963	3.9934	310.47	0.0000	-2.3075	-2.3671
	143->156	-0.30732					
	144->155	-0.28766					
	144->156	0.36008					
3	136->155	0.45994	4.4839	276.51	0.0002	-2.5153	-8.5065
	137->156	0.46048					
4	136->156	0.45955	4.4839	276.51	0.0002	-0.3637	5.5903
	137->155	0.46079					
5	147->155	-0.34282	4.6685	265.58	0.1820	42.6693	42.8692
	148->156	0.41943					
	149->155	0.24464					
6	147->156	-0.35113	4.6702	265.48	0.0049	-46.8788	-44.2103
	148->155	0.4389					
	149->156	0.26748					
7	152->155	0.37289	4.7083	263.33	0.8858	24.9984	23.1153
	153->156	-0.36495					
8	152->156	0.39119	4.7222	262.55	0.0117	-33.365	-33.3159

	153->155	-0.38394					
9	150->159	0.25408	5.4944	225.65	0.0005	-15.5247	-15.8056
	150->161	0.27292					
10	150->162	0.30656	5.4972	225.54	0.0020	7.0947	5.8423
11	150->156	0.33844	5.8311	212.62	0.0001	2.2638	1.9287
	154->155	0.37398					
12	150->155	0.338	5.8321	212.59	0.0001	-0.6988	-0.5867
	154->156	0.37344					
13	151->156	0.40775	6.0551	204.76	0.0082	-4.7108	-4.4327
	152->155	0.23128					
	154->155	-0.36545					
14	151->155	0.40679	6.0557	204.74	0.0004	-0.0488	-0.043
	152->156	0.23241					
	154->156	-0.36599					
15	152->157	0.34365	6.1052	203.08	0.0085	-87.0756	-76.7167
	153->158	0.33785					
16	152->158	0.34372	6.1054	203.07	0.0299	93.5562	79.8402
	153->157	0.33764					
17	151->156	0.4754	6.3973	193.81	0.0030	-0.3888	-0.3969
	153->156	0.32024					
	154->155	0.36342					
18	151->155	0.47826	6.3979	193.79	0.0000	0.112	0.1307
	153->155	0.32063					
	154->156	0.36139					
19	139->155	-0.46228	6.5102	190.45	0.0605	-717.0288	-779.5229
	140->156	0.46359					
20	139->156	0.46653	6.5104	190.44	0.2326	714.0744	775.4332
	140->155	-0.46447					
21	145->155	0.25706	6.5579	189.06	0.0353	-4.0912	-4.3199
	146->156	0.53859					
22	145->156	0.24671	6.5587	189.04	0.0006	3.997	3.6094
	146->155	0.5209					
	150->155	0.22699					
23	147->158	0.25037	6.5612	188.96	0.1353	39.1134	37.9999
	148->157	0.3321					
	149->158	-0.22539					
	152->160	0.23419					
24	147->157	0.24449	6.5618	188.95	0.0142	-3.4381	-3.5222
	148->158	0.32444					
25	152->155	0.33507	6.6372	186.80	0.2139	-4.5656	-4.6413
	153->156	0.24561					
26	152->156	0.34653	6.6410	186.70	0.0007	-2.7796	-2.773
	153->155	0.2594					
27	141->155	0.24463	6.7280	184.28	0.5566	-12.1309	-12.125
	142->156	0.23886					
	153->156	0.24028					
28	141->156	0.29047	6.7412	183.92	0.0002	-2.094	-2.4064
	142->155	0.29957					
	153->155	0.22818					
29	135->156	-0.25789	6.7758	182.98	0.1980	-11.5953	-11.6823
	138->155	0.28928					

30	129->155	-0.2292	6.7853	182.72	0.0016	4.4327	4.8072
	135->155	-0.29704					
	138->156	0.32002					
31	147->155	0.2676	6.8232	181.71	0.0063	0.7601	0.716
	149->155	0.34328					
	150->156	-0.30931					
	152->155	-0.23301					
32	147->156	0.26864	6.8233	181.71	0.0003	-0.4701	-0.5473
	149->156	0.34539					
	150->155	-0.30986					
	152->156	-0.23724					
33	151->159	0.26777	6.8449	181.13	0.1886	8.3526	9.5812
	151->161	0.32546					
	154->162	-0.28335					
34	151->162	-0.31872	6.8557	180.85	0.0012	2.1728	3.1462
	154->159	0.29608					
	154->161	0.32927					
35	145->155	0.49814	6.9982	177.17	0.0581	-19.0411	-19.0309
	146->156	-0.23861					
36	145->156	0.50645	6.9988	177.15	0.0003	0.5188	0.6636
	146->155	-0.24503					

[a]Number of the excited states; [b]Only transitions with contribution over 10.0% were listed; [c]Configuration-interaction coefficient; [d]Excitation energy; [e]Wavelength; [f]Oscillator strength; [g]Rotatory strength in velocity form (10^{-40} cgs); [h]Rotatory strength in length form (10^{-40} cgs).

Table S12. Key transitions, oscillator strengths, and rotatory strengths in the ECD spectrum of conformer 1a-2 at the CAM-B3LYP/6-311G(d) level of theory in MeOH with IEFPCM solvent model.

Num[a]	***Transition***[b]	***CI-coeff***[b]	***ΔE (eV)***[d]	***λ (nm)***[e]	f[f]	R_{vel}[g]	R_{len}[h]
1	142->155	0.67092	3.9904	310.71	0.0000	-0.9701	-0.9954
2	143->156	0.66777	3.9956	310.30	0.0000	-3.0009	-3.0752
3	136->155	0.65434	4.4827	276.58	0.0002	-0.8172	-0.4516
4	137->156	0.66135	4.4836	276.53	0.0001	-0.8592	-0.88
5	147->155	-0.35987	4.6567	266.25	0.2528	24.1084	31.088
	154->155	0.46261					
6	148->156	0.55542	4.6708	265.45	0.0744	20.2701	20.8635
	152->156	-0.22925					
7	147->155	0.40678	4.6869	264.53	0.3469	66.7732	62.9236
	154->155	0.39576					
8	150->156	0.22579	4.7213	262.61	0.3670	-88.2368	-89.9132
	152->156	0.53516					
	153->156	-0.2288					
9	150->161	0.22875	5.4929	225.72	0.0010	-0.961	-2.0377
10	146->162	0.30321	5.5233	224.48	0.0027	-6.1265	-5.9958
	154->162	0.26333					
11	152->155	0.30923	5.8471	212.04	0.0002	-1.4486	-1.3595
	153->155	0.49071					
12	149->156	0.2285	5.8632	211.46	0.0003	-0.2047	-0.2852
	150->156	0.41441					
	153->156	0.33651					
13	146->155	0.38027	6.0415	205.22	0.0033	-6.5309	-6.7922
	153->155	-0.25871					

	154->155	0.24421					
14	154->157	0.48136	6.0741	204.12	0.0328	7.4438	5.1721
15	152->158	0.4815	6.1083	202.98	0.0191	3.5971	1.9682
16	147->155	0.27376	6.1855	200.44	0.0094	0.2226	0.3715
	149->155	0.49986					
	150->155	-0.24134					
17	150->156	-0.2779	6.2190	199.36	0.0046	-2.4689	-2.6027
	151->156	0.56158					
	154->156	-0.23292					
18	149->156	-0.28517	6.2862	197.23	0.0005	0.3885	0.3738
	152->156	0.28585					
	153->156	0.51573					
19	144->155	0.37325	6.3959	193.85	0.0074	-3.5758	-4.9778
	145->155	-0.23934					
	150->155	0.23269					
	153->155	0.30141					
20	151->155	0.5795	6.4198	193.13	0.0005	-0.6633	-0.7209
21	144->155	-0.33762	6.4902	191.03	0.0059	-4.7792	-5.9928
	150->155	0.37148					
	152->155	-0.32132					
22	139->155	0.66315	6.5021	190.68	0.1501	-12.3372	-12.5702
23	140->156	0.65674	6.5120	190.39	0.1460	18.5472	19.6434
24	154->156	0.63398	6.5141	190.33	0.0021	-2.0538	-2.1817
25	147->157	0.46131	6.5536	189.18	0.1724	11.3828	10.9646
26	148->158	0.39853	6.5612	188.97	0.0731	18.0261	17.66
27	148->158	-0.2494	6.5918	188.09	0.0614	-0.0872	-0.3614
	149->156	0.39629					
	150->156	-0.24803					
	151->156	-0.26302					
28	150->155	0.41923	6.6963	185.15	0.0002	-0.0376	-0.0535
	152->155	0.48153					
29	141->156	0.35538	6.7489	183.71	0.4589	-6.3088	-6.6617
	148->158	0.27637					
30	145->155	0.45135	6.8039	182.23	0.0787	-1.8734	-2.6359
31	129->156	0.30447	6.8112	182.03	0.0113	1.7593	1.9663
	135->156	0.35541					
	141->156	0.33184					
32	130->155	0.23112	6.8555	180.85	0.1705	8.615	9.0489
	145->155	0.35263					
33	130->155	0.36985	6.8931	179.87	0.1480	-19.0008	-20.1219
34	145->156	0.44771	6.9413	178.62	0.0073	0.4851	0.5539
	146->156	0.39155					
	149->156	-0.24082					
35	151->159	0.24213	6.9587	178.17	0.1050	-7.7794	-7.9944
	151->161	0.28774					
	151->162	-0.28047					
36	147->155	-0.23877	6.9650	178.01	0.0001	-0.0543	-0.0538
	148->155	0.62627					

[a]Number of the excited states; [b]Only transitions with contribution over 10.0% were listed; [c]Configuration-interaction coefficient; [d]Excitation energy; [e]Wavelength; [f]Oscillator strength; [g]Rotatory strength in velocity form (10^{-40} cgs); [h]Rotatory strength in length form (10^{-40} cgs).

Table S13. Key transitions, oscillator strengths, and rotatory strengths in the ECD spectrum of conformer 1a-3 at the CAM-B3LYP/6-311G(d) level of theory in MeOH with IEFPCM solvent model.

Num[a]	*Transition*[b]	*CI-coeff*[b]	*ΔE (eV)*[d]	*λ (nm)*[e]	*f*[f]	R_{vel}[g]	R_{len}[h]
1	142->155	0.67092	3.9904	310.71	0.0000	-0.9701	-0.9954
2	143->156	0.66777	3.9956	310.30	0.0000	-3.0009	-3.0755
3	136->155	0.65434	4.4827	276.58	0.0002	-0.817	-0.4519
4	137->156	0.66135	4.4836	276.53	0.0001	-0.861	-0.8801
5	147->155	-0.35989	4.6567	266.25	0.2528	24.0956	31.0768
	154->155	0.46258					
6	148->156	0.55542	4.6708	265.45	0.0744	20.2745	20.8679
	152->156	-0.22924					
7	147->155	0.40676	4.6869	264.53	0.3470	66.7887	62.9395
	154->155	0.39578					
8	150->156	0.2258	4.7213	262.61	0.3670	-88.2432	-89.9212
	152->156	0.53516					
	153->156	-0.22881					
9	150->161	0.22875	5.4929	225.72	0.0010	-0.9618	-2.0385
10	146->162	0.3032	5.5233	224.48	0.0027	-6.1271	-5.9953
	154->162	0.26332					
11	152->155	0.30924	5.8471	212.04	0.0002	-1.4486	-1.3595
	153->155	0.4907					
12	149->156	0.22846	5.8632	211.46	0.0003	-0.2046	-0.2851
	150->156	0.41442					
	153->156	0.33651					
13	146->155	0.38027	6.0415	205.22	0.0033	-6.5316	-6.7933
	153->155	-0.2587					
	154->155	0.24421					
14	154->157	0.48135	6.0741	204.12	0.0328	7.4444	5.1731
15	152->158	0.48149	6.1083	202.98	0.0191	3.6002	1.9695
16	147->155	0.27376	6.1855	200.44	0.0094	0.2231	0.3718
	149->155	0.49988					
	150->155	-0.2413					
17	150->156	-0.2779	6.2190	199.36	0.0046	-2.4691	-2.6029
	151->156	0.56157					
	154->156	-0.23292					
18	149->156	-0.28516	6.2862	197.23	0.0005	0.3885	0.3737
	152->156	0.28585					
	153->156	0.51572					
19	144->155	0.37325	6.3959	193.85	0.0074	-3.5761	-4.9776
	145->155	-0.23934					
	150->155	0.23269					
	153->155	0.30141					
20	151->155	0.57949	6.4198	193.13	0.0005	-0.6632	-0.7208
21	144->155	-0.33761	6.4902	191.03	0.0059	-4.7783	-5.9917
	150->155	0.37149					
	152->155	-0.32132					
22	139->155	0.66315	6.5021	190.68	0.1501	-12.3459	-12.5878
23	140->156	0.65676	6.5120	190.39	0.1460	18.5483	19.6458
24	154->156	0.63399	6.5141	190.33	0.0020	-2.0487	-2.1761
25	147->157	0.46131	6.5536	189.18	0.1724	11.3884	10.9684

26	148->158	0.39854	6.5612	188.97	0.0731	18.0213	17.6565
27	148->158	-0.24939	6.5918	188.09	0.0614	-0.0881	-0.3622
	149->156	0.39632					
	150->156	-0.248					
	151->156	-0.26303					
28	150->155	0.41924	6.6963	185.15	0.0002	-0.0375	-0.0535
	152->155	0.48152					
29	141->156	0.35537	6.7489	183.71	0.4589	-6.3062	-6.6593
	148->158	0.27637					
30	145->155	0.4513	6.8039	182.23	0.0787	-1.8786	-2.6406
31	129->156	0.30445	6.8112	182.03	0.0113	1.7645	1.9717
	135->156	0.3554					
	141->156	0.33182					
32	130->155	0.23107	6.8555	180.85	0.1705	8.6194	9.051
	145->155	0.35266					
33	130->155	0.36987	6.8931	179.87	0.1480	-19.0019	-20.1227
34	145->156	0.44771	6.9413	178.62	0.0073	0.4852	0.554
	146->156	0.39154					
	149->156	-0.24083					
35	151->159	0.24213	6.9587	178.17	0.1050	-7.7805	-7.9954
	151->161	0.28773					
	151->162	-0.28048					
36	147->155	-0.23878	6.9650	178.01	0.0001	-0.0543	-0.0538
	148->155	0.62627					

[a]Number of the excited states; [b]Only transitions with contribution over 10.0% were listed; [c]Configuration-interaction coefficient; [d]Excitation energy; [e]Wavelength; [f]Oscillator strength; [g]Rotatory strength in velocity form (10^{-40} cgs); [h]Rotatory strength in length form (10^{-40} cgs).

Table S14. Key transitions, oscillator strengths, and rotatory strengths in the ECD spectrum of conformer 1a-4 at the CAM-B3LYP/6-311G(d) level of theory in MeOH with IEFPCM solvent model.

Num[a]	***Transition***[b]	***CI-coeff***[b]	***ΔE (eV)***[d]	***λ (nm)***[e]	***f***[f]	R_{vel}[g]	R_{len}[h]
1	142->155	0.67091	3.9904	310.71	0.0000	-0.9696	-0.9966
2	143->156	0.66774	3.9956	310.30	0.0000	-2.9694	-3.0448
3	136->155	0.65428	4.4827	276.58	0.0002	-0.8188	-0.4486
4	137->156	0.66144	4.4836	276.53	0.0001	-0.9069	-0.8975
5	147->155	-0.35973	4.6567	266.25	0.2528	24.1152	31.0792
	154->155	0.4625					
6	148->156	0.5555	4.6708	265.44	0.0744	20.2765	20.8688
	152->156	-0.2291					
7	147->155	0.40666	4.6869	264.53	0.3469	66.8756	63.0206
	154->155	0.39572					
8	150->156	0.22683	4.7213	262.61	0.3670	-88.362	-90.0295
	152->156	0.53475					
	153->156	-0.22954					
9	150->161	0.23094	5.4929	225.72	0.0010	-1.0129	-2.0601
10	146->162	0.30337	5.5233	224.48	0.0027	-6.1237	-5.9926
	154->162	0.26336					
11	152->155	0.31003	5.8472	212.04	0.0002	-1.4481	-1.3598
	153->155	0.49007					
12	150->156	0.41706	5.8634	211.45	0.0003	-0.2058	-0.2831
	153->156	0.33658					

13	146->155	0.38039	6.0415	205.22	0.0033	-6.5314	-6.7916
	153->155	-0.25871					
	154->155	0.2445					
14	154->157	0.4813	6.0741	204.12	0.0328	7.4387	5.1747
15	152->158	0.48114	6.1083	202.98	0.0191	3.6046	1.9684
16	147->155	0.27465	6.1856	200.44	0.0094	0.224	0.3734
	149->155	0.50258					
	150->155	-0.23472					
17	150->156	-0.27749	6.2185	199.38	0.0045	-2.4744	-2.6056
	151->156	0.56093					
	154->156	-0.23309					
18	149->156	-0.28452	6.2867	197.22	0.0005	0.3882	0.3721
	152->156	0.28658					
	153->156	0.51473					
19	144->155	0.3718	6.3959	193.85	0.0074	-3.589	-4.9922
	145->155	-0.23906					
	150->155	0.23347					
	153->155	0.30284					
20	151->155	0.57873	6.4192	193.15	0.0005	-0.6437	-0.7037
21	144->155	-0.33647	6.4905	191.02	0.0059	-4.8661	-6.0932
	150->155	0.37228					
	152->155	-0.3213					
22	139->155	0.66304	6.5021	190.68	0.1501	-12.2434	-12.4555
23	140->156	0.65678	6.5120	190.39	0.1460	18.4958	19.639
24	154->156	0.63405	6.5141	190.33	0.0020	-2.0553	-2.1811
25	147->157	0.46116	6.5537	189.18	0.1724	11.3797	10.9665
26	148->158	0.39866	6.5612	188.97	0.0731	18.0444	17.6805
27	148->158	-0.24931	6.5918	188.09	0.0615	-0.1083	-0.3853
	149->156	0.39927					
	150->156	-0.24294					
	151->156	-0.26286					
28	150->155	0.4209	6.6964	185.15	0.0002	-0.0371	-0.0533
	152->155	0.48111					
29	141->156	0.35538	6.7489	183.71	0.4588	-6.2596	-6.6148
	148->158	0.27645					
30	145->155	0.44642	6.8043	182.21	0.0808	-2.2381	-3.0299
31	129->156	0.3032	6.8113	182.03	0.0105	2.2094	2.4591
	135->156	0.35433					
	141->156	0.32876					
32	130->155	0.23032	6.8558	180.85	0.1693	8.5494	8.9827
	145->155	0.35555					
33	130->155	0.36983	6.8932	179.87	0.1480	-18.9823	-20.1041
34	145->156	0.44776	6.9414	178.62	0.0073	0.488	0.5576
	146->156	0.39137					
	149->156	-0.24169					
35	151->159	0.24251	6.9585	178.18	0.1044	-7.7317	-7.9557
	151->161	0.2875					
	151->162	-0.28059					
36	147->155	-0.23826	6.9650	178.01	0.0001	-0.0523	-0.0521
	148->155	0.62645					

[a]Number of the excited states; [b]Only transitions with contribution over 10.0% were listed; [c]Configuration-

interaction coefficient; [d]Excitation energy; [e]Wavelength; [f]Oscillator strength; [g]Rotatory strength in velocity form (10^{-40} cgs); [h]Rotatory strength in length form (10^{-40} cgs).

Table S15. Key transitions, oscillator strengths, and rotatory strengths in the ECD spectrum of conformer 1a-5 at the CAM-B3LYP/6-311G(d) level of theory in MeOH with IEFPCM solvent model.

Num[a]	***Transition***[b]	***CI-coeff***[b]	***ΔE (eV)***[d]	***λ (nm)***[e]	***f***[f]	R_{vel}[g]	R_{len}[h]
1	142->155	0.67092	3.9904	310.71	0.0000	-0.9687	-0.9964
2	143->156	0.66775	3.9956	310.30	0.0000	-2.9701	-3.045
3	136->155	0.65428	4.4827	276.58	0.0002	-0.8182	-0.4488
4	137->156	0.66143	4.4836	276.53	0.0001	-0.9075	-0.8979
5	147->155	-0.35971	4.6567	266.25	0.2528	24.1115	31.075
	154->155	0.4625					
6	148->156	0.55548	4.6708	265.45	0.0744	20.2859	20.8781
	152->156	-0.22913					
7	147->155	0.40666	4.6869	264.53	0.3469	66.8727	63.0198
	154->155	0.39572					
8	150->156	0.22682	4.7213	262.61	0.3670	-88.365	-90.0324
	152->156	0.53473					
	153->156	-0.22952					
9	150->161	0.23094	5.4929	225.72	0.0010	-1.0129	-2.0594
10	146->162	0.30338	5.5233	224.48	0.0027	-6.1223	-5.9927
	154->162	0.26336					
11	152->155	0.31003	5.8472	212.04	0.0002	-1.4481	-1.3599
	153->155	0.49008					
12	150->156	0.41704	5.8634	211.46	0.0003	-0.206	-0.2831
	153->156	0.33658					
13	146->155	0.38039	6.0415	205.22	0.0033	-6.5324	-6.7925
	153->155	-0.2587					
	154->155	0.2445					
14	154->157	0.48129	6.0741	204.12	0.0328	7.4384	5.1737
15	152->158	0.48115	6.1083	202.98	0.0191	3.6076	1.9711
16	147->155	0.27464	6.1856	200.44	0.0094	0.224	0.3734
	149->155	0.50257					
	150->155	-0.23475					
17	150->156	-0.27753	6.2185	199.38	0.0045	-2.4743	-2.6055
	151->156	0.56091					
	154->156	-0.23309					
18	149->156	-0.28453	6.2867	197.22	0.0005	0.3883	0.3721
	152->156	0.28657					
	153->156	0.51473					
19	144->155	0.3718	6.3960	193.85	0.0074	-3.5885	-4.9918
	145->155	-0.23906					
	150->155	0.23347					
	153->155	0.30284					
20	151->155	0.57872	6.4192	193.15	0.0005	-0.6436	-0.7036
21	144->155	-0.33646	6.4905	191.02	0.0059	-4.8668	-6.094
	150->155	0.37229					
	152->155	-0.3213					
22	139->155	0.66304	6.5021	190.68	0.1501	-12.2532	-12.4653
23	140->156	0.6568	6.5120	190.39	0.1460	18.5209	19.652
24	154->156	0.63408	6.5141	190.33	0.0020	-2.0485	-2.1742

25	147->157	0.46115	6.5537	189.18	0.1724	11.3741	10.9605
26	148->158	0.39863	6.5612	188.97	0.0730	18.0475	17.6832
27	148->158	-0.24934	6.5918	188.09	0.0615	-0.1082	-0.3852
	149->156	0.39923					
	150->156	-0.24295					
	151->156	-0.26287					
28	150->155	0.42088	6.6964	185.15	0.0002	-0.0371	-0.0533
	152->155	0.48111					
29	141->156	0.35539	6.7489	183.71	0.4588	-6.2605	-6.6157
	148->158	0.27646					
30	145->155	0.44641	6.8043	182.21	0.0808	-2.2393	-3.0316
31	129->156	0.30319	6.8113	182.03	0.0105	2.2095	2.4592
	135->156	0.35433					
	141->156	0.32874					
32	130->155	0.2303	6.8558	180.85	0.1693	8.5487	8.984
	145->155	0.35555					
33	130->155	0.36984	6.8932	179.87	0.1480	-18.9838	-20.1057
34	145->156	0.44776	6.9414	178.62	0.0073	0.4877	0.5572
	146->156	0.39137					
	149->156	-0.24169					
35	151->159	0.2425	6.9585	178.18	0.1044	-7.7339	-7.9581
	151->161	0.2875					
	151->162	-0.28058					
36	147->155	-0.23829	6.9650	178.01	0.0001	-0.0523	-0.0521
	148->155	0.62645					

[a]Number of the excited states; [b]Only transitions with contribution over 10.0% were listed; [c]Configuration-interaction coefficient; [d]Excitation energy; [e]Wavelength; [f]Oscillator strength; [g]Rotatory strength in velocity form (10^{-40} cgs); [h]Rotatory strength in length form (10^{-40} cgs).

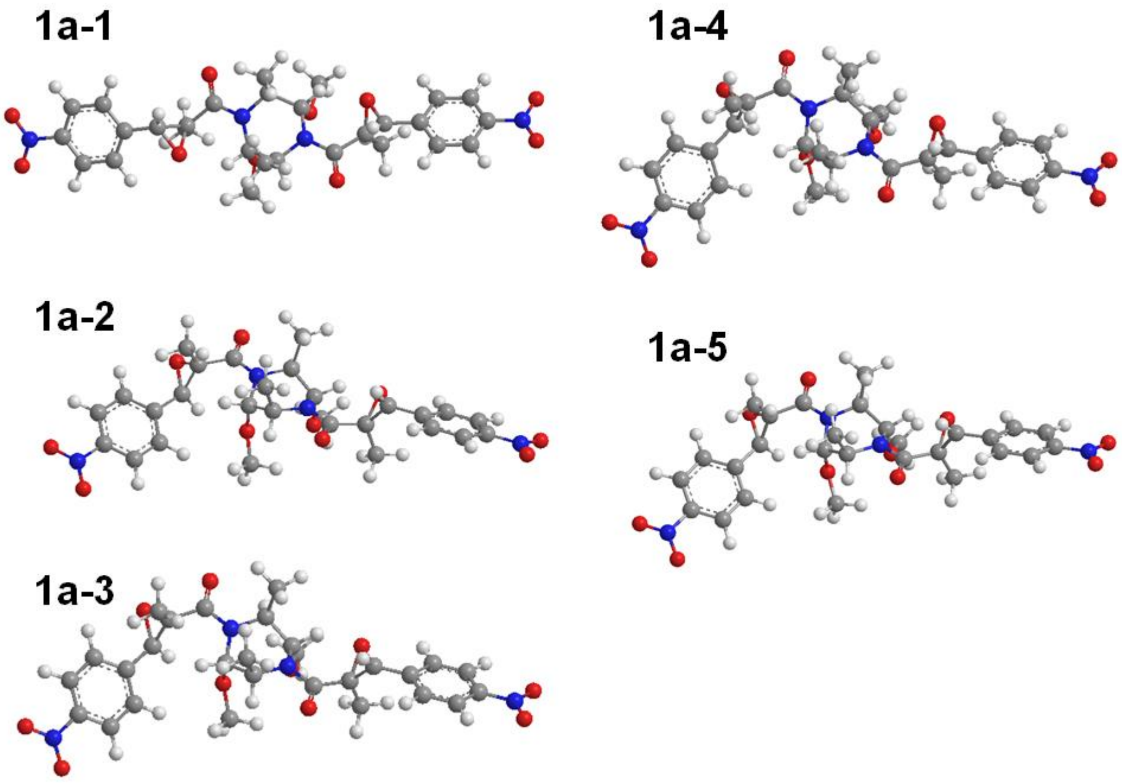

Figure S25. Optimized geometries of 5 dominant conformers of **1a** (**1a-1** to **1a-5**, respectively) at the B3LYP/6-31G(d) level of theory in the gas phase

Table S16. Conformational analysis of the B3LYP/6-31G(d) optimized conformers of 2 in the gas phase (T=298.15 K)

Conformer	E[a] (Hartree)	C[b] (Hartree)	G[c] (kcal/mol)	ΔG[d] (kcal/mol)	Population[e]
2-1	-1978.494826	0.451065	-1241222.459789	0.0	31.56%
2-2	-1978.494825	0.451069	-1241222.456633	0.003156	31.40%
2-3	-1978.494825	0.451069	-1241222.456614	0.003175	31.39%
2-4	-1978.49237	0.450233	-1241221.440892	1.018897	5.65%

[a]Electronic energy obtained at M062X/6-311+G(2d,p) level of theory; [b]Thermal correction to Gibbs free energy obtained at B3LYP/6-31G(d) level of theory; [c]Gibbs free energy (E + C); [d]The relative Gibbs free energy; [e]The Boltzmann distribution of each conformer.

Table S17. Atomic coordinates (Å) of 2-1 obtained at the B3LYP/6-31G(d) level of theory in the gas phase.

C	1.157411	1.403705	0.246611	O	-10.459185	-2.172999	-0.089736
C	-0.205944	1.901398	-0.269420	O	-2.555716	1.665936	-1.576439
N	-1.307818	1.061556	0.227032	N	10.149494	-0.653004	-0.725476
C	-2.442491	1.033799	-0.527697	O	10.687534	-1.751255	-0.592982
C	-3.601736	0.138582	-0.107587	O	10.719194	0.366913	-1.110881
C	-4.834486	0.841605	0.312579	O	3.919253	0.978546	0.387815
C	-6.200278	0.306521	0.065221	H	1.896254	1.705193	-0.495613
C	-6.679076	-0.808664	0.762928	H	-0.191596	1.760759	-1.354507
C	-7.954845	-1.302584	0.509848	H	-4.770867	1.929154	0.365059
C	-8.740760	-0.665832	-0.447209	H	-6.044473	-1.273483	1.508900
C	-8.291326	0.450165	-1.151138	H	-8.349217	-2.161132	1.038342
C	-7.016352	0.935021	-0.885439	H	-8.938095	0.914561	-1.884445

C	-0.438729	3.383295	0.029477	H	-6.645498	1.803024	-1.423071
C	-3.622724	-1.194610	-0.817004	H	0.403459	3.985388	-0.327810
O	1.485158	2.019031	1.487559	H	-1.345161	3.707790	-0.485556
C	-1.046983	0.238800	1.408451	H	-0.552446	3.546066	1.102712
C	0.090418	-0.746749	1.063113	H	-2.767638	-1.806103	-0.506585
N	1.186310	-0.045427	0.351327	H	-3.551552	-1.030575	-1.896561
C	2.205726	-0.803307	-0.154547	H	-4.540240	-1.744962	-0.600012
C	3.521784	-0.108107	-0.490858	H	2.422443	1.791508	1.634903
C	4.577477	-0.293793	0.534239	H	-1.935469	-0.349490	1.616534
C	6.027445	-0.362038	0.210156	H	-0.321679	-1.480999	0.362293
C	6.693359	-1.588061	0.342232	H	4.282988	-0.842566	1.429776
C	8.044514	-1.691979	0.033355	H	6.148733	-2.464355	0.682024
C	8.718821	-0.552084	-0.399820	H	8.580493	-2.628232	0.122161
C	8.083580	0.679726	-0.530360	H	8.650536	1.539706	-0.863405
C	6.729581	0.769031	-0.222240	H	6.207850	1.715811	-0.304324
C	0.602641	-1.467893	2.309478	H	1.335254	-2.228346	2.033595
C	3.827182	0.034942	-1.959064	H	1.051927	-0.754940	3.005204
O	-0.828175	1.006163	2.560440	H	-0.231442	-1.957156	2.822844
O	-4.011022	0.160687	1.272705	H	4.852387	0.371266	-2.124101
O	2.141960	-2.018781	-0.293149	H	3.142193	0.751152	-2.426458
N	-10.087819	-1.184013	-0.721326	H	3.687120	-0.936428	-2.442856
O	-10.764002	-0.598893	-1.567146	H	0.037316	1.455485	2.472735

Table S18. Atomic coordinates (Å) of 2-2 obtained at the B3LYP/6-31G(d) level of theory in the gas phase.

C	1.047050	0.238400	-1.408427	O	-10.719206	0.367571	1.110854
C	-0.090411	-0.747106	-1.063097	O	-2.142355	-2.019036	0.292653
N	-1.186281	-0.045724	-0.351340	N	10.088063	-1.183488	0.721401
C	-2.205917	-0.803530	0.154187	O	10.764332	-0.598013	1.566907
C	-3.521883	-0.108183	0.490472	O	10.459412	-2.172651	0.090074
C	-4.577650	-0.293883	-0.534524	O	4.011057	0.160348	-1.272510
C	-6.027595	-0.361995	-0.210265	H	1.935539	-0.349913	-1.616431
C	-6.729700	0.769288	0.221622	H	0.321634	-1.481346	-0.362239
C	-8.083670	0.680125	0.529905	H	-4.283303	-0.842854	-1.429990
C	-8.718906	-0.551760	0.400042	H	-6.207953	1.716100	0.303238
C	-8.044624	-1.691873	-0.032591	H	-8.650607	1.540264	0.862578
C	-6.693493	-1.588101	-0.341628	H	-8.580599	-2.628180	-0.120861
C	-0.602581	-1.468256	-2.309468	H	-6.148881	-2.464568	-0.680999
C	-3.827231	0.035242	1.958646	H	0.231536	-1.957538	-2.822766
O	0.828336	1.005721	-2.560461	H	-1.335227	-2.228686	-2.033627
C	-1.157418	1.403415	-0.246804	H	-1.051798	-0.755312	-3.005242
C	0.205927	1.901143	0.269252	H	-3.142115	0.751442	2.425870
N	1.307827	1.061239	-0.227056	H	-3.687337	-0.936036	2.442674
C	2.442431	1.033519	0.527778	H	-4.852381	0.371786	2.123590
C	3.601767	0.138352	0.107750	H	-0.037197	1.454977	-2.472872
C	4.834465	0.841467	-0.312455	H	-1.896315	1.704970	0.495338
C	6.200310	0.306534	-0.065088	H	0.191528	1.760596	1.354350
C	7.016506	0.935438	0.885212	H	4.770733	1.929001	-0.365039
C	8.291541	0.450751	1.150895	H	6.645702	1.803637	1.422561
C	8.740932	-0.665491	0.447311	H	8.938413	0.915455	1.883919
C	7.954909	-1.302631	-0.509392	H	8.349244	-2.161344	-1.037646
C	6.679068	-0.808870	-0.762459	H	6.044407	-1.273980	-1.508200

C	0.438780	3.383001	-0.029750	H	1.345210	3.707490	0.485296
C	3.622742	-1.194771	0.817319	H	0.552554	3.545676	-1.102991
O	-1.485120	2.018611	-1.487835	H	-0.403394	3.985153	0.327467
O	-3.919289	0.978407	-0.388393	H	4.540499	-1.744942	0.600889
O	2.555551	1.665695	1.576506	H	2.767970	-1.806493	0.506484
N	-10.149549	-0.652532	0.725870	H	3.550940	-1.030622	1.896813
O	-10.687581	-1.750863	0.593979	H	-2.422426	1.791150	-1.635149

Table S19. Atomic coordinates (Å) of 2-3 obtained at the B3LYP/6-31G(d) level of theory in the gas phase.

C	1.047053	0.238449	-1.408441	O	-10.687572	-1.750894	0.593971
C	-0.090407	-0.747067	-1.063140	O	-2.142348	-2.019039	0.292569
N	-1.186278	-0.045706	-0.351365	N	10.088049	-1.183527	0.721406
C	-2.205912	-0.803529	0.154143	O	10.764312	-0.598080	1.566936
C	-3.521877	-0.108194	0.490458	O	10.459400	-2.172671	0.090050
C	-4.577651	-0.293870	-0.534536	O	4.011064	0.160394	-1.272515
C	-6.027594	-0.361995	-0.210268	H	1.935543	-0.349858	-1.616459
C	-6.693491	-1.588098	-0.341662	H	0.321637	-1.481326	-0.362302
C	-8.044620	-1.691881	-0.032619	H	-4.283309	-0.842816	-1.430018
C	-8.718901	-0.551781	0.400052	H	-6.148880	-2.464554	-0.681061
C	-8.083665	0.680101	0.529946	H	-8.580593	-2.628186	-0.120912
C	-6.729698	0.769275	0.221657	H	-8.650601	1.540230	0.862648
C	-0.602575	-1.468184	-2.309531	H	-6.207952	1.716086	0.303296
C	-3.827215	0.035191	1.958638	H	0.231544	-1.957448	-2.822843
O	0.828342	1.005799	-2.560457	H	-1.335220	-2.228623	-2.033712
C	-1.157418	1.403430	-0.246792	H	-1.051794	-0.755221	-3.005285
C	0.205925	1.901147	0.269279	H	-3.142095	0.751377	2.425877
N	1.307827	1.061259	-0.227050	H	-3.687317	-0.936101	2.442638
C	2.442429	1.033519	0.527787	H	-4.852363	0.371730	2.123599
C	3.601764	0.138359	0.107741	H	-0.037193	1.455050	-2.472860
C	4.834468	0.841481	-0.312436	H	-1.896317	1.704966	0.495356
C	6.200309	0.306535	-0.065073	H	0.191525	1.760572	1.354372
C	7.016499	0.935406	0.885255	H	4.770741	1.929017	-0.364989
C	8.291529	0.450706	1.150934	H	6.645693	1.803590	1.422627
C	8.740923	-0.665515	0.447319	H	8.938396	0.915384	1.883979
C	7.954906	-1.302622	-0.509411	H	8.349242	-2.161320	-1.037690
C	6.679069	-0.808848	-0.762475	H	6.044413	-1.273933	-1.508236
C	0.438775	3.383014	-0.029684	H	1.345203	3.707492	0.485372
C	3.622729	-1.194784	0.817272	H	0.552549	3.545718	-1.102920
O	-1.485118	2.018656	-1.487809	H	-0.403401	3.985154	0.327548
O	-3.919293	0.978419	-0.388374	H	4.540486	-1.744953	0.600832
O	2.555545	1.665668	1.576532	H	2.767957	-1.806493	0.506413
N	-10.149541	-0.652565	0.725887	H	3.550921	-1.030667	1.896770
O	-10.719197	0.367526	1.110904	H	-2.422423	1.791194	-1.635132

Table S20. Atomic coordinates (Å) of 2-4 obtained at the B3LYP/6-31G(d) level of theory in the gas phase.

C	-1.013575	-3.275534	-0.791873	O	8.613168	3.855723	1.270898
C	0.306089	-2.871307	-1.462592	O	2.151831	-1.101725	-2.343264
N	1.155005	-2.086923	-0.551132	N	-8.013577	3.727724	-0.471271
C	2.049918	-1.234473	-1.124909	O	-8.230582	4.474499	0.481980
C	2.928296	-0.359163	-0.239543	O	-8.678396	3.698036	-1.506086
C	4.372955	-0.681128	-0.254090	O	-2.806221	0.107841	1.106978

C	5.435707	0.351151	-0.119264	H	-1.760875	-3.390771	-1.577832
C	5.670797	0.993253	1.102232	H	0.040590	-2.194224	-2.279813
C	6.660348	1.965604	1.207100	H	4.661081	-1.564953	-0.824570
C	7.407741	2.283601	0.076008	H	5.076199	0.714199	1.964752
C	7.198738	1.655028	-1.150450	H	6.863852	2.474082	2.140929
C	6.209650	0.682943	-1.239766	H	7.805396	1.933115	-2.002697
C	1.056282	-4.070760	-2.044913	H	6.028845	0.181465	-2.186257
C	2.408165	1.048856	-0.072884	H	0.403043	-4.648744	-2.706899
O	-0.890245	-4.529476	-0.116450	H	1.907730	-3.706104	-2.623034
C	0.864861	-2.185750	0.877115	H	1.412231	-4.726033	-1.247903
C	-0.570135	-1.661190	1.118675	H	1.477839	1.045492	0.506974
N	-1.505589	-2.230922	0.101290	H	2.196160	1.474430	-1.058439
C	-2.868702	-2.282813	0.300921	H	3.135112	1.681651	0.439889
C	-3.588885	-1.069114	0.852189	H	-1.803588	-4.772025	0.121956
C	-3.600717	0.103193	-0.078515	H	1.537705	-1.519245	1.408526
C	-4.738992	1.055559	-0.164870	H	-0.536512	-0.579803	0.968739
C	-5.026867	1.936212	0.884997	H	-3.020265	-0.008630	-0.995847
C	-6.100245	2.816066	0.790185	H	-4.395903	1.929436	1.766561
C	-6.876487	2.801463	-0.365614	H	-6.341843	3.508758	1.586286
C	-6.607714	1.941047	-1.428164	H	-7.236256	1.967910	-2.309017
C	-5.529768	1.070207	-1.321798	H	-5.302263	0.393241	-2.140426
C	-1.000705	-1.964697	2.553776	H	-1.926679	-1.448889	2.812812
C	-4.728895	-1.368362	1.791377	H	-1.125962	-3.040186	2.701239
O	1.116693	-3.463150	1.392729	H	-0.224905	-1.623047	3.246405
O	3.566183	-0.967333	0.899114	H	-5.324208	-0.475375	1.986189
O	-3.545052	-3.239942	-0.077172	H	-4.342530	-1.745665	2.744280
N	8.451963	3.311927	0.178572	H	-5.363645	-2.140156	1.350353
O	9.102139	3.568914	-0.834427	H	0.441729	-4.079011	1.038232

Table S21. Key transitions, oscillator strengths, and rotatory strengths in the ECD spectrum of conformer 2-1 at the CAM-B3LYP/6-311G(d) level of theory in MeOH with IEFPCM solvent model.

Num[a]	*Transition*[b]	*CI-coeff*[b]	*ΔE (eV)*[d]	*λ (nm)*[e]	*f*[f]	R_{vel}[g]	R_{len}[h]
1	137->148	0.66273	3.9906	310.69	0.0001	-3.2526	-3.3334
2	136->147	0.662	4.0004	309.93	0.0000	-3.1803	-3.2552
3	129->147	0.66216	4.4799	276.76	0.0001	-0.4688	-0.5453
4	130->148	0.67155	4.4833	276.55	0.0001	-1.2198	-1.2771
5	141->148	0.60762	4.6687	265.56	0.0794	-2.6934	-0.0985
	145->148	-0.26282					
6	140->147	0.61574	4.6798	264.93	0.0422	2.4172	3.282
7	141->148	0.23033	4.7186	262.76	0.6286	37.7447	36.3399
	143->148	-0.27084					
	145->148	0.55501					
8	144->147	0.6261	4.7586	260.55	0.3265	-42.8442	-43.5643
9	139->151	0.43358	5.4711	226.62	0.0009	-6.5618	-7.148
	139->153	0.30452					
	139->154	-0.22908					
10	143->148	0.23462	5.5785	222.25	0.0011	-5.2507	-7.0326
	143->152	0.22582					
	143->154	0.38663					
11	143->148	0.50862	5.8490	211.98	0.0006	-0.9156	-1.0284
	145->148	0.23375					

12	146->148	0.66145	5.9757	207.48	0.0036	-0.6955	-0.7397
13	142->147	0.33711	6.0378	205.35	0.0085	-0.1196	-0.4114
	146->147	0.53441					
14	139->147	0.4796	6.0688	204.30	0.0030	-5.4751	-5.57
	142->147	-0.34849					
15	141->152	-0.25211	6.1084	202.97	0.0190	5.4147	3.0544
	145->150	0.50815					
16	144->149	0.53393	6.1324	202.18	0.0165	4.1428	2.7394
17	139->147	-0.30462	6.4034	193.62	0.0071	-1.797	-1.9776
	142->147	-0.37369					
	146->147	0.43541					
18	133->148	0.65535	6.5084	190.50	0.1373	37.6649	40.659
19	131->147	0.65426	6.5143	190.33	0.1578	-34.7099	-37.6154
20	142->148	0.63997	6.5593	189.02	0.0043	-1.2002	-1.3217
21	141->150	0.49824	6.5649	188.86	0.1186	22.4546	21.2776
	145->152	0.33826					
22	140->149	0.53387	6.5732	188.62	0.1401	11.5703	10.9876
	144->151	-0.31109					
23	142->147	0.2508	6.6006	187.84	0.0030	-0.6438	-0.6728
	143->147	0.40334					
	145->147	0.50703					
24	134->148	0.27513	6.7024	184.98	0.3277	1.3741	0.965
	135->148	0.35286					
	141->150	0.29242					
25	143->147	0.51474	6.7397	183.96	0.0037	-0.4559	-0.4689
	145->147	-0.45962					
26	142->151	0.31163	6.7561	183.51	0.4486	-92.2617	-96.8648
	142->153	0.27142					
27	139->148	-0.28437	6.7856	182.72	0.0016	0.0717	0.0683
	144->148	0.62081					
28	122->148	-0.34878	6.7983	182.37	0.0890	-0.4527	-0.3472
	128->148	0.4368					
29	121->147	0.39684	6.8480	181.05	0.0864	20.6632	21.4165
	127->147	0.37557					
30	132->147	0.4155	6.8508	180.98	0.1499	27.4019	28.658
	134->147	0.23102					
	140->149	-0.22835					
31	139->148	0.5758	6.9119	179.38	0.0024	0.2731	0.3309
	144->148	0.31201					
32	146->152	0.23164	6.9168	179.25	0.1925	-25.5946	-27.5877
	146->153	0.27261					
	146->154	0.40838					
33	141->147	0.69923	7.0048	177.00	0.0000	0.0045	0.0048
34	122->148	0.47414	7.0530	175.79	0.1576	-28.2023	-25.4112
	128->148	0.22506					
35	121->147	0.47946	7.0594	175.63	0.0750	-14.9078	-14.549
36	140->148	0.67431	7.0731	175.29	0.0001	-0.0206	-0.0164

[a]Number of the excited states; [b]Only transitions with contribution over 10.0% were listed; [c]Configuration-interaction coefficient; [d]Excitation energy; [e]Wavelength; [f]Oscillator strength; [g]Rotatory strength in velocity form (10^{-40} cgs); [h]Rotatory strength in length form (10^{-40} cgs).

Table S22. Key transitions, oscillator strengths, and rotatory strengths in the ECD spectrum of conformer 2-2 at the CAM-B3LYP/6-311G(d) level of theory in MeOH with IEFPCM solvent model.

Num[a]	*Transition*[b]	*CI-coeff*[b]	*ΔE (eV)*[d]	*λ (nm)*[e]	*f*[f]	R_{vel}[g]	R_{len}[h]
1	137->148	0.66273	3.9906	310.69	0.0001	-3.2421	-3.3239
2	136->147	0.66203	4.0004	309.93	0.0000	-3.1881	-3.2634
3	129->147	0.66218	4.4798	276.76	0.0001	-0.4574	-0.536
4	130->148	0.67154	4.4832	276.55	0.0001	-1.2265	-1.2853
5	141->148	0.60765	4.6687	265.57	0.0794	-2.659	-0.0656
	145->148	-0.26283					
6	140->147	0.61567	4.6798	264.93	0.0422	2.3863	3.2533
7	141->148	0.2304	4.7186	262.76	0.6282	37.6703	36.2586
	143->148	-0.27094					
	145->148	0.55502					
8	144->147	0.6262	4.7586	260.54	0.3269	-42.7615	-43.4815
9	139->151	0.43363	5.4711	226.62	0.0009	-6.5615	-7.1532
	139->153	0.30476					
	139->154	-0.22897					
10	143->148	0.23488	5.5784	222.26	0.0011	-5.254	-7.032
	143->152	0.22584					
	143->154	0.3866					
11	143->148	0.50848	5.8489	211.98	0.0006	-0.9188	-1.033
	145->148	0.23384					
12	146->148	0.66147	5.9756	207.48	0.0036	-0.697	-0.7413
13	142->147	0.33629	6.0380	205.34	0.0085	-0.0911	-0.3787
	146->147	0.53452					
14	139->147	0.47927	6.0688	204.30	0.0030	-5.4943	-5.5907
	142->147	-0.34937					
15	141->152	-0.25212	6.1084	202.97	0.0190	5.4103	3.0525
	145->150	0.50811					
16	144->149	0.53395	6.1324	202.18	0.0165	4.1341	2.7375
17	139->147	-0.30447	6.4034	193.62	0.0071	-1.7934	-1.9738
	142->147	-0.37376					
	146->147	0.4355					
18	133->148	0.65519	6.5084	190.50	0.1374	37.4715	40.4298
19	131->147	0.65429	6.5143	190.33	0.1577	-34.49	-37.3772
20	142->148	0.64003	6.5593	189.02	0.0043	-1.2003	-1.3218
21	141->150	0.49821	6.5649	188.86	0.1185	22.4124	21.2326
	145->152	0.33828					
22	140->149	0.53392	6.5733	188.62	0.1404	11.6108	11.0283
	144->151	-0.31095					
23	142->147	0.25068	6.6005	187.84	0.0030	-0.6426	-0.6716
	143->147	0.40309					
	145->147	0.50732					
24	134->148	0.27514	6.7024	184.98	0.3277	1.3679	0.962
	135->148	0.35281					
	141->150	0.29253					
25	143->147	0.51496	6.7398	183.96	0.0037	-0.4563	-0.4694
	145->147	-0.45934					
26	142->151	0.31184	6.7562	183.51	0.4487	-92.2606	-96.8686
	142->153	0.27162					
27	139->148	-0.28453	6.7857	182.71	0.0016	0.0723	0.069

	144->148	0.62073					
28	122->148	-0.34884	6.7983	182.37	0.0890	-0.4289	-0.3234
	128->148	0.43672					
29	121->147	0.39755	6.8480	181.05	0.0847	20.2921	21.0305
	127->147	0.3755					
30	132->147	0.41503	6.8509	180.98	0.1514	27.7366	29.0091
	134->147	0.23058					
	140->149	-0.22931					
31	139->148	0.57615	6.9118	179.38	0.0022	0.2881	0.3462
	144->148	0.31241					
32	146->152	0.23188	6.9169	179.25	0.1928	-25.5868	-27.5775
	146->153	0.2726					
	146->154	0.4088					
33	141->147	0.69924	7.0048	177.00	0.0000	0.0045	0.0048
34	122->148	0.47488	7.0529	175.79	0.1563	-27.9333	-25.1355
	128->148	0.22547					
35	121->147	0.48006	7.0595	175.63	0.0764	-15.1883	-14.8364
36	140->148	0.67418	7.0731	175.29	0.0001	-0.0205	-0.0163

[a]Number of the excited states; [b]Only transitions with contribution over 10.0% were listed; [c]Configuration-interaction coefficient; [d]Excitation energy; [e]Wavelength; [f]Oscillator strength; [g]Rotatory strength in velocity form (10^{-40} cgs); [h]Rotatory strength in length form (10^{-40} cgs).

Table S23. Key transitions, oscillator strengths, and rotatory strengths in the ECD spectrum of conformer 2-3 at the CAM-B3LYP/6-311G(d) level of theory in MeOH with IEFPCM solvent model.

Num[a]	***Transition***[b]	***CI-coeff***[b]	***ΔE (eV)***[d]	***λ (nm)***[e]	f[f]	R_{vel}[g]	R_{len}[h]
1	137->148	0.66273	3.9906	310.69	0.0001	-3.2422	-3.3239
2	136->147	0.66203	4.0004	309.93	0.0000	-3.1882	-3.263
3	129->147	0.66218	4.4798	276.76	0.0001	-0.4581	-0.5364
4	130->148	0.67154	4.4832	276.55	0.0001	-1.2268	-1.285
5	141->148	0.60764	4.6687	265.57	0.0794	-2.6608	-0.0665
	145->148	-0.26285					
6	140->147	0.61568	4.6798	264.93	0.0422	2.387	3.253
7	141->148	0.23042	4.7186	262.76	0.6282	37.6773	36.2647
	143->148	-0.27093					
	145->148	0.55501					
8	144->147	0.6262	4.7586	260.55	0.3268	-42.7682	-43.4887
9	139->151	0.43362	5.4711	226.62	0.0009	-6.5614	-7.1532
	139->153	0.30476					
	139->154	-0.22897					
10	143->148	0.23487	5.5784	222.26	0.0011	-5.2537	-7.0319
	143->152	0.22584					
	143->154	0.3866					
11	143->148	0.50849	5.8489	211.98	0.0006	-0.9185	-1.0328
	145->148	0.23384					
12	146->148	0.66147	5.9756	207.48	0.0036	-0.6968	-0.7412
13	142->147	0.33627	6.0380	205.34	0.0085	-0.0905	-0.378
	146->147	0.53452					
14	139->147	0.47926	6.0688	204.30	0.0030	-5.4946	-5.5911
	142->147	-0.34938					
15	141->152	-0.25212	6.1084	202.97	0.0190	5.4106	3.0525
	145->150	0.50811					

16	144->149	0.53395	6.1324	202.18	0.0165	4.1369	2.7387
17	139->147	-0.30447	6.4034	193.62	0.0071	-1.7931	-1.9735
	142->147	-0.37376					
	146->147	0.4355					
18	133->148	0.65519	6.5084	190.50	0.1374	37.5126	40.4715
19	131->147	0.65429	6.5143	190.33	0.1577	-34.5317	-37.4189
20	142->148	0.64002	6.5593	189.02	0.0043	-1.2002	-1.3218
21	141->150	0.49821	6.5649	188.86	0.1185	22.4098	21.2312
	145->152	0.33828					
22	140->149	0.53391	6.5733	188.62	0.1404	11.6103	11.028
	144->151	-0.31095					
23	142->147	0.25069	6.6005	187.84	0.0030	-0.6427	-0.6716
	143->147	0.40309					
	145->147	0.50732					
24	134->148	0.27514	6.7024	184.98	0.3277	1.3698	0.9645
	135->148	0.35281					
	141->150	0.29253					
25	143->147	0.51496	6.7398	183.96	0.0037	-0.456	-0.469
	145->147	-0.45934					
26	142->151	0.31183	6.7562	183.51	0.4487	-92.2637	-96.8707
	142->153	0.27161					
27	139->148	-0.28453	6.7857	182.71	0.0016	0.0722	0.0689
	144->148	0.62073					
28	122->148	-0.34883	6.7983	182.37	0.0889	-0.4335	-0.3279
	128->148	0.43672					
29	121->147	0.39759	6.8480	181.05	0.0846	20.2738	21.0114
	127->147	0.37549					
30	132->147	0.415	6.8509	180.98	0.1515	27.7621	29.0356
	134->147	0.23054					
	140->149	-0.22937					
31	139->148	0.57614	6.9118	179.38	0.0022	0.2878	0.3459
	144->148	0.3124					
32	146->152	0.23187	6.9169	179.25	0.1928	-25.5946	-27.5849
	146->153	0.2726					
	146->154	0.40879					
33	141->147	0.69924	7.0048	177.00	0.0000	0.0045	0.0048
34	122->148	0.47488	7.0529	175.79	0.1563	-27.9346	-25.1358
	128->148	0.22547					
35	121->147	0.48006	7.0595	175.63	0.0764	-15.1884	-14.8365
36	140->148	0.67418	7.0731	175.29	0.0001	-0.0205	-0.0163

[a]Number of the excited states; [b]Only transitions with contribution over 10.0% were listed; [c]Configuration-interaction coefficient; [d]Excitation energy; [e]Wavelength; [f]Oscillator strength; [g]Rotatory strength in velocity form (10^{-40} cgs); [h]Rotatory strength in length form (10^{-40} cgs).

Table S24. Key transitions, oscillator strengths, and rotatory strengths in the ECD spectrum of conformer 2-4 at the CAM-B3LYP/6-311G(d) level of theory in MeOH with IEFPCM solvent model.

Num[a]	***Transition***[b]	***CI-coeff***[b]	***ΔE (eV)***[d]	***λ (nm)***[e]	***f***	R_{vel}^{g}	R_{len}^{h}
1	137->148	0.67038	3.9915	310.62	0.0001	-3.4265	-3.4886
2	136->147	0.66439	3.9977	310.14	0.0000	-3.1236	-3.2149
3	129->147	0.66326	4.4788	276.82	0.0002	-1.1351	-1.1541
4	130->148	0.67211	4.4834	276.54	0.0001	-1.0585	-1.1781

5	140->147	0.55109	4.6641	265.83	0.1337	48.3817	45.2041
	145->147	0.27925					
6	141->148	0.58772	4.6693	265.53	0.0580	-26.2376	-22.9785
	144->148	0.23171					
7	140->147	-0.30651	4.7023	263.67	0.4793	132.5246	135.7052
	145->147	0.46076					
8	143->148	-0.23999	4.7249	262.40	0.4170	-157.0194	-160.2278
	144->148	0.49704					
	145->147	-0.23524					
9	139->147	-0.25169	5.2467	236.31	0.0007	7.3599	11.6267
	139->151	0.44064					
	142->151	0.23536					
10	143->148	-0.2599	5.5807	222.16	0.0009	-5.5715	-7.0686
	143->152	0.25026					
	143->154	0.40416					
11	143->148	0.51977	5.8554	211.74	0.0012	-1.4369	-1.4328
	143->154	0.22918					
12	142->147	0.44595	5.8755	211.02	0.0581	-16.8619	-16.2038
	142->151	-0.30513					
13	146->148	0.64821	5.9804	207.32	0.0033	-0.5612	-0.5187
14	141->152	-0.24709	6.1089	202.96	0.0102	15.0188	11.1715
	144->150	0.46513					
15	145->149	0.45426	6.1104	202.91	0.0267	-6.053	-4.4329
16	139->147	0.29827	6.1297	202.27	0.0022	-4.1929	-3.5751
	146->147	0.49389					
17	139->147	-0.35892	6.2498	198.38	0.0061	-7.6868	-7.5663
	142->147	-0.24732					
	146->147	0.42243					
18	143->147	0.47558	6.4625	191.85	0.0065	-1.9724	-1.9025
	144->147	0.45826					
19	142->147	0.354	6.4815	191.29	0.1486	-18.2638	-15.9102
	142->151	0.39282					
20	142->148	0.36468	6.5041	190.63	0.0069	7.8898	8.3478
	145->148	0.54965					
21	131->147	0.64425	6.5055	190.58	0.1018	15.6905	17.1876
22	133->148	0.63354	6.5096	190.46	0.1832	-46.1064	-50.4959
23	140->149	0.37071	6.5159	190.28	0.0059	-2.1133	-1.9165
	145->151	0.38739					
24	141->150	0.49902	6.5651	188.85	0.1056	19.6765	17.9395
	144->152	0.32855					
25	143->147	0.48942	6.6071	187.65	0.0003	0.0201	0.0282
	144->147	-0.46218					
26	142->148	0.56231	6.6252	187.14	0.0004	0.0564	0.0717
	145->148	-0.36019					
27	127->147	-0.24837	6.6832	185.52	0.4424	101.563	97.5456
	140->149	0.42058					
28	134->148	0.34708	6.7071	184.85	0.1741	-63.0017	-63.6006
	138->148	-0.29971					
	141->150	-0.2787					
29	135->147	-0.22846	6.7733	183.05	0.2246	16.3739	17.1069
	139->147	0.25052					

	140->149	0.28164					
	145->151	-0.2401					
30	123->148	0.32834	6.7910	182.57	0.1317	-42.8867	-42.9694
	127->148	-0.2542					
	128->148	0.36918					
31	141->147	0.70191	6.8754	180.33	0.0000	-0.0009	-0.0012
32	146->151	0.23346	6.9093	179.45	0.2322	6.091	3.9733
	146->152	0.244					
	146->153	0.29961					
	146->154	0.39513					
33	140->148	0.70285	6.9416	178.61	0.0002	-0.0235	-0.0236
34	122->147	-0.38345	6.9860	177.48	0.0268	-8.6214	-8.9425
	139->148	0.49306					
35	122->147	0.39992	6.9897	177.38	0.0355	3.1637	3.5694
	139->148	0.46155					
36	123->148	0.47391	7.0522	175.81	0.1254	-27.8555	-23.7193

[a]Number of the excited states; [b]Only transitions with contribution over 10.0% were listed; [c]Configuration-interaction coefficient; [d]Excitation energy; [e]Wavelength; [f]Oscillator strength; [g]Rotatory strength in velocity form (10^{-40} cgs); [h]Rotatory strength in length form (10^{-40} cgs).

Table S25. Conformational analysis of the B3LYP/6-31G(d) optimized conformers of 3 in the gas phase (T=298.15 K)

Conformer	E[a] (Hartree)	C[b] (Hartree)	G[c] (kcal/mol)	ΔG[d] (kcal/mol)	Population[e]
3-1	-2017.786948	0.473915	-1265863.928289	0.0	97.16%
3-2	-2017.783689	0.473988	-1265861.837302	2.090987	2.84%

[a]Electronic energy obtained at M062X/6-311+G(2d,p) level of theory; [b]Thermal correction to Gibbs free energy obtained at B3LYP/6-31G(d) level of theory; [c]Gibbs free energy (E + C); [d]The relative Gibbs free energy; [e]The Boltzmann distribution of each conformer.

Table S26. Atomic coordinates (Å) of 3-1 obtained at the B3LYP/6-31G(d) level of theory in the gas phase.

C	2.744002	-1.659307	2.507698	O	4.026648	0.832072	1.762025
C	3.769585	-1.886190	1.404612	N	-7.322286	-1.891984	0.089308
N	3.590951	-0.903903	0.335344	O	-7.945384	-2.833658	-0.400482
C	3.595771	0.423987	0.690631	O	-7.830249	-0.974045	0.732221
C	2.880027	1.430691	-0.202255	O	-0.909090	-0.715644	-0.121385
C	1.409455	1.441535	0.038417	H	2.937697	-2.252624	3.424771
C	0.571232	2.663438	-0.024370	H	3.476921	-2.860405	0.992273
C	-0.035567	3.121678	1.153336	H	1.042944	0.664845	0.706058
C	-0.823221	4.266262	1.141132	H	0.115395	2.576925	2.080757
C	-1.004274	4.938606	-0.066445	H	-1.297881	4.642929	2.038110
C	-0.425020	4.496888	-1.253297	H	-0.602564	5.045836	-2.169310
C	0.365223	3.352466	-1.225533	H	0.816953	2.973440	-2.135275
C	5.196518	-2.006500	1.957866	H	5.863436	-2.365003	1.171612
C	3.670504	2.662143	-0.556362	H	5.545208	-1.040922	2.326827
O	1.767697	-0.948032	2.423367	H	5.221587	-2.731210	2.779736
C	3.683563	-1.348130	-1.051384	H	4.441618	2.416970	-1.295748
C	2.602083	-2.368808	-1.462303	H	4.162404	3.037722	0.344384
N	1.300241	-1.970392	-0.964426	H	3.029147	3.442995	-0.968863
C	0.668609	-2.630788	0.038477	H	3.586320	-0.458107	-1.676785
C	-0.671928	-2.029645	0.433841	H	2.831626	-3.329831	-0.997114
C	-1.649598	-1.808490	-0.674523	H	0.978481	-1.033374	-1.182008
C	-3.122652	-1.811230	-0.471981	H	-1.321282	-2.106751	-1.671502

C	-3.755707	-0.785978	0.241524	H	-3.156530	0.026457	0.636764
C	-5.133891	-0.806238	0.428420	H	-5.646989	-0.023961	0.973150
C	-5.866218	-1.862879	-0.106983	H	-5.872340	-3.691216	-1.229066
C	-5.262886	-2.891817	-0.827540	H	-3.396927	-3.647710	-1.571559
C	-3.885664	-2.856160	-1.010381	H	2.279970	-1.592125	-3.466473
C	2.581136	-2.529902	-2.983838	H	3.573903	-2.805393	-3.348762
C	-1.100425	-2.283855	1.853228	H	1.868412	-3.307703	-3.271911
C	6.006902	-1.056974	-1.409099	H	-1.018590	-3.354044	2.058919
O	1.953190	0.934408	-1.193427	H	-2.128634	-1.959500	2.020521
O	1.090956	-3.651871	0.577751	H	-0.434251	-1.753182	2.537590
N	-1.838518	6.147712	-0.088970	H	5.845584	-0.350667	-2.236905
O	-1.980071	6.724107	-1.167296	H	6.150021	-0.490226	-0.479994
O	-2.344752	6.512173	0.971960	H	6.902936	-1.647558	-1.610020
O	4.926973	-1.972692	-1.304269	-	-	-	-

Table S27. Atomic coordinates (Å) of 3-2 obtained at the B3LYP/6-31G(d) level of theory in the gas phase.

C	1.960390	-2.718502	2.139649	O	2.938446	-0.248964	2.192417
C	3.163752	-2.760600	1.196088	N	-7.563418	-0.760219	0.128554
N	3.149451	-1.558400	0.344065	O	-8.328252	-1.261248	-0.694915
C	2.958515	-0.361227	0.968398	O	-7.918090	-0.125413	1.120886
C	2.713580	0.891927	0.135664	O	-1.035011	-0.869441	0.197999
C	1.331659	1.419308	0.206947	H	1.127780	-2.075399	1.793579
C	1.008354	2.868012	0.122715	H	2.967748	-3.608740	0.530706
C	0.563369	3.527928	1.276276	H	0.612833	0.819367	0.762746
C	0.263544	4.884582	1.239828	H	0.458912	2.975703	2.205779
C	0.406233	5.566872	0.033052	H	-0.076399	5.418316	2.118067
C	0.835966	4.933076	-1.129663	H	0.923866	5.502806	-2.045998
C	1.137446	3.575690	-1.077893	H	1.466070	3.049950	-1.967289
C	4.477883	-2.994281	1.938733	H	5.278075	-3.230064	1.233627
C	3.916628	1.785504	-0.033266	H	4.744361	-2.109332	2.521040
O	1.870584	-3.384999	3.141838	H	4.349510	-3.833689	2.626848
C	3.344622	-1.712402	-1.097549	H	4.680109	1.292913	-0.644924
C	2.192102	-2.465290	-1.801492	H	4.346788	1.991163	0.951861
N	0.910634	-2.067744	-1.247364	H	3.644922	2.730835	-0.506492
C	0.145875	-2.934033	-0.531306	H	3.423762	-0.708760	-1.522310
C	-1.083303	-2.315167	0.118306	H	2.300396	-3.531963	-1.594262
C	-1.947349	-1.452580	-0.740475	H	0.741687	-1.072100	-1.138424
C	-3.402256	-1.258645	-0.499615	H	-1.637266	-1.349183	-1.781480
C	-3.863690	-0.541698	0.610924	H	-3.142534	-0.113507	1.297643
C	-5.228224	-0.374325	0.822583	H	-5.609941	0.178138	1.671712
C	-6.119838	-0.933033	-0.089461	H	-6.417678	-2.059982	-1.889883
C	-5.687981	-1.646012	-1.205926	H	-3.964798	-2.350352	-2.274361
C	-4.321571	-1.800906	-1.407685	H	2.065375	-1.170733	-3.541625
C	2.251645	-2.226403	-3.310786	H	3.237273	-2.500887	-3.697271
C	-1.621543	-3.072431	1.303429	H	1.493912	-2.828807	-3.819344
C	5.708624	-1.686526	-1.203996	H	-1.829171	-4.101510	1.000255
O	1.810180	0.771612	-0.992160	H	-2.535857	-2.613772	1.682174
O	0.389212	-4.129771	-0.397427	H	-0.881538	-3.111317	2.107983
N	0.089106	7.001710	-0.014655	H	5.738329	-0.838131	-1.903539
O	0.227731	7.580601	-1.091556	H	5.808097	-1.310093	-0.178064
O	-0.294232	7.535695	1.025224	H	6.539610	-2.360166	-1.421057

O	4.516778	-2.440718	-1.371402	-	-	-	-

Table S28. Key transitions, oscillator strengths, and rotatory strengths in the ECD spectrum of conformer 3-1 at the CAM-B3LYP/6-311G(d) level of theory in MeOH with IEFPCM solvent model.

Num[a]	*Transition*[b]	*CI-coeff*[b]	*ΔE (eV)*[d]	*λ (nm)*[e]	*f*[f]	R_{vel}[g]	R_{len}[h]
1	140->151	0.504	3.9905	310.70	0.0000	-2.678	-2.7053
	140->152	-0.44253					
2	139->151	0.44208	3.9930	310.50	0.0001	-3.3702	-3.4039
	139->152	0.5034					
3	142->153	0.39373	4.4645	277.71	0.0001	4.0324	5.5816
	143->153	0.28007					
	146->153	0.19592					
	150->153	-0.2471					
4	133->151	0.43137	4.4810	276.69	0.0002	-1.4148	-1.6014
	133->152	0.49449					
5	132->151	0.4916	4.4812	276.67	0.0002	-0.8184	-0.7815
	132->152	-0.43445					
6	144->151	-0.26009	4.6511	266.57	0.1445	46.0778	40.0704
	144->152	0.30383					
	147->151	-0.20784					
	148->151	-0.23807					
	149->151	0.19396					
	150->152	0.28373					
7	144->151	0.21321	4.6601	266.06	0.0931	-30.9623	-31.8145
	145->151	0.34148					
	145->152	0.4366					
8	144->151	0.25909	4.6802	264.91	0.1016	-181.1747	-177.4597
	145->151	-0.23556					
	146->152	0.31653					
	150->152	0.24255					
9	146->151	0.26681	4.7086	263.31	0.7282	138.8309	140.6662
	147->152	-0.23066					
	148->152	-0.227					
	149->152	0.27465					
	150->151	0.35475					
10	148->156	0.24392	5.3208	233.02	0.0000	-4.5775	-1.6271
11	143->159	0.20231	5.4812	226.20	0.0001	-3.8879	-1.5695
12	148->151	0.31235	5.8776	210.94	0.0168	3.2289	3.5895
	148->152	-0.26219					
	149->151	0.28163					
	149->152	-0.21636					
	150->151	-0.26438					
	150->152	0.20763					
13	147->151	0.2844	5.9441	208.58	0.0114	0.5753	0.6453
	147->152	0.29692					
	149->151	0.23899					
	149->152	0.29155					
	150->152	-0.22223					
14	147->151	0.27074	5.9540	208.24	0.0002	-0.692	-0.1564
	147->152	-0.254					
	150->151	-0.2669					

	150->152	0.24478					
15	149->151	0.37889	5.9867	207.10	0.0004	1.0684	1.3768
	149->152	-0.34675					
	150->151	0.24104					
	150->152	-0.19837					
16	142->153	-0.23385	6.0492	204.96	0.0075	4.7798	4.376
	148->153	0.40435					
	149->153	0.29062					
17	150->152	0.18879	6.0945	203.44	0.0090	1.8228	2.6316
	150->154	0.19138					
18	147->154	-0.2267	6.0964	203.37	0.0128	11.3158	10.2419
	148->154	-0.2412					
	150->155	0.24161					
19	145->157	-0.19896	6.1089	202.96	0.0146	-8.746	-7.0841
	149->155	0.23347					
	150->155	0.21544					
20	147->151	0.23114	6.1851	200.46	0.0005	1.4131	1.4821
	147->152	0.25917					
	149->151	-0.20787					
	149->152	-0.23963					
	150->152	0.19002					
21	144->151	0.19083	6.2536	198.26	0.0004	0.2323	0.159
	146->151	0.27079					
	146->152	-0.25675					
	147->151	-0.28623					
	147->152	0.29098					
22	148->151	0.38948	6.2618	198.00	0.0008	0.2187	0.2625
	148->152	0.42887					
23	142->151	0.38456	6.3966	193.83	0.0074	-10.4925	-10.5479
	142->152	-0.3342					
	145->151	-0.25625					
	145->152	0.22177					
24	142->151	0.20391	6.4094	193.44	0.0022	-4.3886	-4.378
	145->151	0.43172					
	145->152	-0.38145					
25	144->151	0.38309	6.4626	191.85	0.0003	-0.0252	-0.0189
	144->152	0.43675					
	146->151	-0.25339					
	146->152	-0.29025					
26	143->151	0.41482	6.4708	191.61	0.0001	-0.2266	-0.17
	143->152	-0.36756					
	146->151	-0.19233					
27	142->153	0.20188	6.4946	190.90	0.0124	-25.7896	-25.4042
	147->153	-0.22199					
	148->153	0.22697					
	149->153	-0.21281					
	150->153	0.21072					
28	135->151	0.20146	6.5009	190.72	0.0920	170.639	184.8076
	135->152	0.27547					
	136->151	0.43106					
	136->152	-0.35387					

29	135->151	0.38611	6.5057	190.58	0.1893	-145.372	-158.7945
	135->152	0.40948					
	136->151	-0.23626					
	136->152	0.24631					
30	144->154	0.25508	6.5243	190.03	0.0162	1.001	1.1007
	148->156	-0.25334					
31	143->152	0.26357	6.5455	189.42	0.0032	4.024	3.683
	146->152	0.20002					
32	145->155	0.40566	6.5562	189.11	0.0702	18.5798	18.7222
	150->157	0.20388					
33	144->154	0.25852	6.6582	186.21	0.2454	-156.4802	-158.7974
	147->153	-0.22215					
34	147->153	0.25987	6.6679	185.94	0.0155	44.5636	44.2477
	149->153	0.19964					
35	134->152	0.19346	6.6964	185.15	0.0784	0.4648	1.7621
	138->151	0.24103					
	138->152	0.25281					
	143->151	0.22445					
36	142->151	0.2992	6.7240	184.39	0.0593	7.1559	8.2915
	142->152	0.3076					

[a]Number of the excited states; [b]Only transitions with contribution over 10.0% were listed; [c]Configuration-interaction coefficient; [d]Excitation energy; [e]Wavelength; [f]Oscillator strength; [g]Rotatory strength in velocity form (10^{-40} cgs); [h]Rotatory strength in length form (10^{-40} cgs).

Table S29. Key transitions, oscillator strengths, and rotatory strengths in the ECD spectrum of conformer 3-2 at the CAM-B3LYP/6-311G(d) level of theory in MeOH with IEFPCM solvent model.

Num[a]	*Transition*[b]	*CI-coeff*[b]	*ΔE (eV)*[d]	*λ (nm)*[e]	*f*[f]	R_{vel}[g]	R_{len}[h]
1	140->151	0.64736	3.9950	310.35	0.0000	-1.9309	-1.9667
2	139->152	0.64943	3.9970	310.19	0.0000	-2.559	-2.6301
3	134->151	0.64204	4.4783	276.85	0.0002	-1.3458	-1.414
4	133->152	0.63206	4.4805	276.72	0.0001	-0.554	-0.6132
5	142->155	-0.28124	4.5503	272.48	0.0009	4.8769	1.8505
	150->155	0.46854					
	150->156	-0.21723					
6	146->151	0.61381	4.6689	265.55	0.0616	9.2544	7.6896
7	145->152	0.59268	4.6788	264.99	0.0308	24.7313	22.6466
8	149->151	0.50185	4.7125	263.10	0.3519	-184.4638	-185.8232
9	147->152	0.22036	4.7618	260.37	0.6092	120.9111	123.2126
	148->152	0.49164					
	149->152	-0.24429					
10	143->151	-0.22138	5.4545	227.31	0.0002	-4.8562	-2.4258
	143->159	0.22285					
11	143->158	0.21563	5.5476	223.49	0.0005	2.84	1.5036
	144->158	0.27596					
12	144->152	-0.22685	6.0042	206.50	0.0031	-3.7882	-3.7949
	147->152	-0.31725					
	150->152	0.4183					
13	147->151	0.43347	6.0186	206.00	0.0127	5.0169	5.1653
	150->151	0.36827					
14	147->152	0.30447	6.0621	204.52	0.0013	2.8643	3.234
	150->152	0.48736					

15	149->154	0.43355	6.1180	202.66	0.0130	4.7021	4.8914
16	136->152	0.21509	6.1303	202.25	0.0182	5.7115	5.2319
	148->153	0.42578					
17	143->151	0.24368	6.1720	200.88	0.0013	1.7944	2.1364
	149->151	-0.30672					
	150->151	0.3947					
18	148->152	0.23015	6.1858	200.43	0.0007	0.3017	0.3611
	149->152	0.62109					
19	144->151	-0.27677	6.2180	199.40	0.0049	-2.0562	-1.0243
	147->151	0.3412					
	150->151	-0.33237					
20	148->151	0.58673	6.3407	195.54	0.0001	-0.1411	-0.1445
21	143->152	-0.2387	6.4348	192.68	0.0017	-0.362	-0.5549
	144->152	-0.39199					
	147->152	0.4273					
22	137->151	0.61405	6.5046	190.61	0.0817	8.5109	9.3902
23	136->152	-0.23078	6.5102	190.44	0.0273	-58.3231	-59.2103
	142->155	0.37553					
	150->155	0.29782					
24	136->152	0.57306	6.5137	190.34	0.2055	72.6779	72.2445
25	145->151	0.5634	6.5416	189.53	0.0022	2.2537	2.3355
	146->152	0.3322					
26	145->151	-0.34366	6.5419	189.52	0.0004	0.4429	0.478
	146->152	0.59318					
27	146->154	0.44086	6.5567	189.09	0.0785	-33.8905	-34.2372
	149->156	0.22958					
28	145->153	0.48153	6.5766	188.52	0.1566	50.321	51.7744
	148->157	-0.24689					
29	143->151	0.27721	6.6152	187.42	0.0070	5.3653	4.5754
	144->151	0.41955					
30	144->151	-0.24145	6.6647	186.03	0.0225	-1.5276	-1.2842
	147->155	0.2272					
	150->158	0.2543					
31	143->152	0.55839	6.6913	185.29	0.0103	1.3452	1.1749
	144->152	-0.33578					
32	127->151	0.24142	6.7567	183.50	0.1320	-22.1289	-22.9038
	132->151	-0.29013					
	138->151	0.34903					
33	142->152	0.60697	6.7764	182.97	0.0006	0.2776	0.2329
34	131->152	0.22337	6.8197	181.80	0.1895	-135.5941	-138.1267
35	146->154	0.2615	6.8485	181.04	0.5241	124.5131	125.5291
36	126->152	0.36427	6.8794	180.23	0.0015	-1.6705	-1.7461
	135->152	-0.2652					

[a]Number of the excited states; [b]Only transitions with contribution over 10.0% were listed; [c]Configuration-interaction coefficient; [d]Excitation energy; [e]Wavelength; [f]Oscillator strength; [g]Rotatory strength in velocity form (10^{-40} cgs); [h]Rotatory strength in length form (10^{-40} cgs).

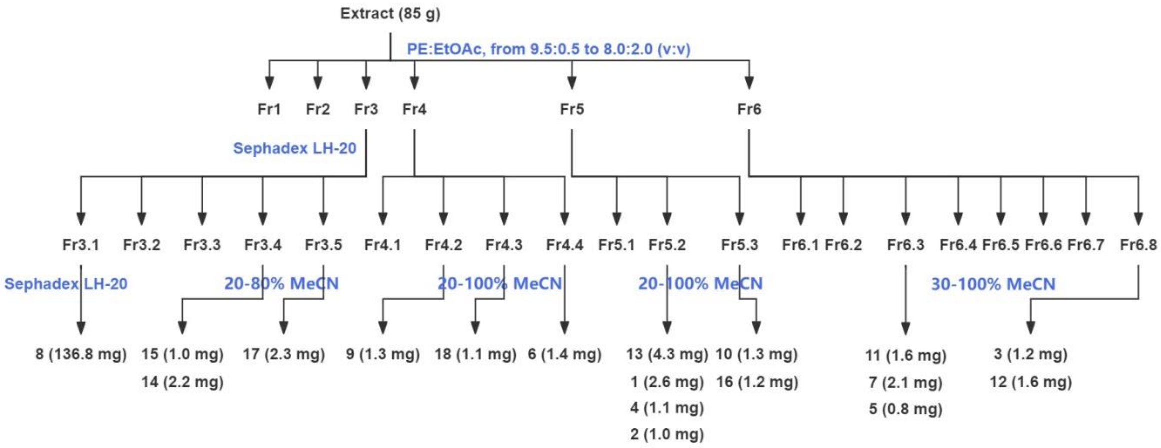

Figure S26. Fractionaion Tree

General experimental procedures

High-resolution electrospray ionization mass spectrometry (HRESIMS) analyses were conducted using a Waters Xevo G2 Q-TOF mass spectrometer. NMR data were obtained via Bruker Avance spectrometers operating at 600 MHz. ECD spectra were recorded on a JASCO Corporation J-715 spectropolarimeter. UV spectra acquisition was performed with a Shimadzu UV-1800 spectrophotometer. Optical rotations were measured using an Anton Paar MCP 500 polarimeter. Various materials were utilized for column chromatography (CC), including silica gel (100-200 and 200-300 mesh from Yantai), Sephadex LH 20 (GE Healthcare Bio-Sciences AB), and RP-C18 (ODS-A, 50 μM, YMC). Preparative high-performance liquid chromatography (HPLC) was executed with a CXTH P3000 pump and a UV3000 ultraviolet-visible detector, employing a preparative RP-C18 column (5 μM, 20 mm × 250 mm, Part Number: AA12S05-2520WT, Ser.No.: 129XA80138, YMC). The .fid data for compounds 1-3 was submitted to ScienceDB, accessible at https://www.scidb.cn/en. Researchers can visit and download the data using the following link: https://www.scidb.cn/en/anonymous/VnZRbnl1.
